# Supplementary material for: Salmonella manipulates macrophage migration via SteC-mediated myosin light chain activation to penetrate the gut-vascular barrier
Source: EMBO J. 2024 Mar 25;43(8):1499–518. doi: 10.1038/s44318-024-00076-7 (PMC11021425; doi:10.1038/s44318-024-00076-7)
Supplement: Supplementary file 1 — Appendix [file 44318_2024_76_MOESM1_ESM.pdf]

Supplemental Information

Salmonella manipulates macrophage migration via SteC-mediated myosin light chain activation to penetrate the gut-vascular barrier

Yuanji Dai, Min Zhang, Xiaoyu Liu, Ting Sun, Wenqi Qi, Wei Ding, Zhe Chen, Ping Zhang, Ruirui Liu, Huimin Chen, Siyan Chen, Yuzhen Wang, Yingying Yue, Nannan Song, Weiwei Wang, Haihong Jia, Zhongrui Ma, Cuiling Li, Qixin Chen and Bingqing Li

Appendix Fig S1–7 pages 2-8

Appendix Table S1-5 pages 9-17

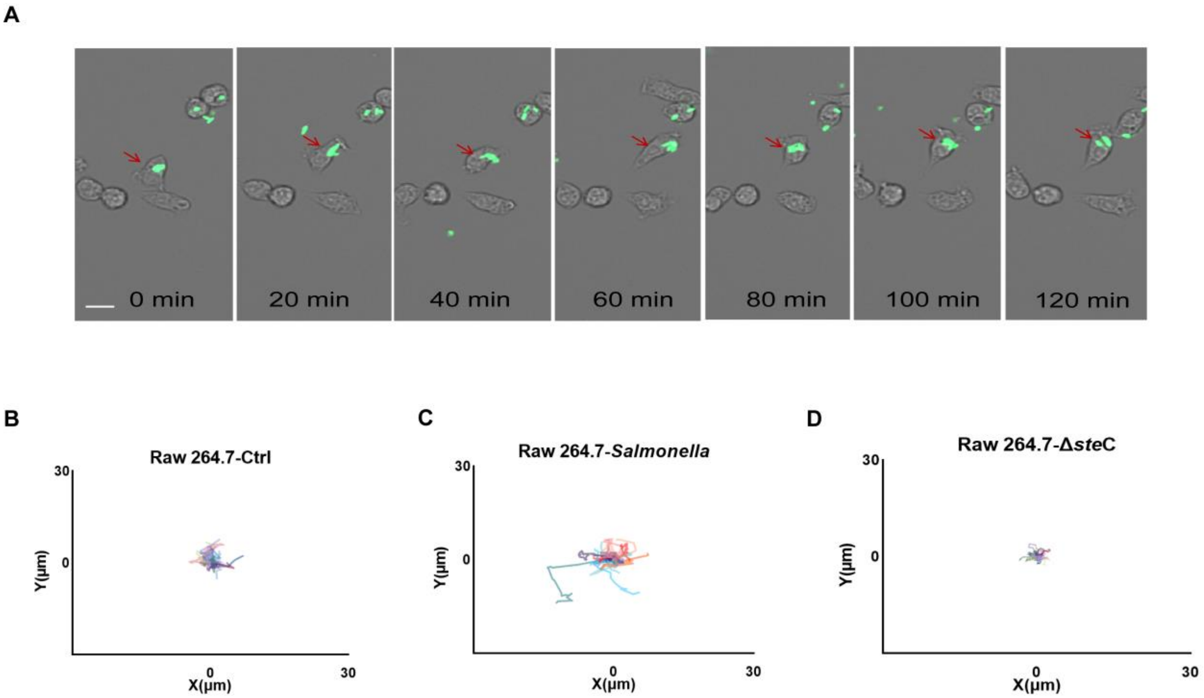

Appendix Fig.S1. ***Salmonella*** **infection stimulates the migratory capacities of RAW264.7 cells, related to Fig.1.**

(**A**) Representative micrograph of RAW264.7 macrophages infected with wild-type *Salmonella* expressing GFP. Confocal time lapse sequences were captured every 20 min. Scale bar, 20 μm. The arrow indicates a *Salmonella*-containing motile cell. (**B**-**D**) The actual movement trajectory of RAW264.7 cells challenged by wild-type or Δ*steC*. The uninfected RAW264.7 cells were used as controls. More than 30 individual cells for each group were calculated.

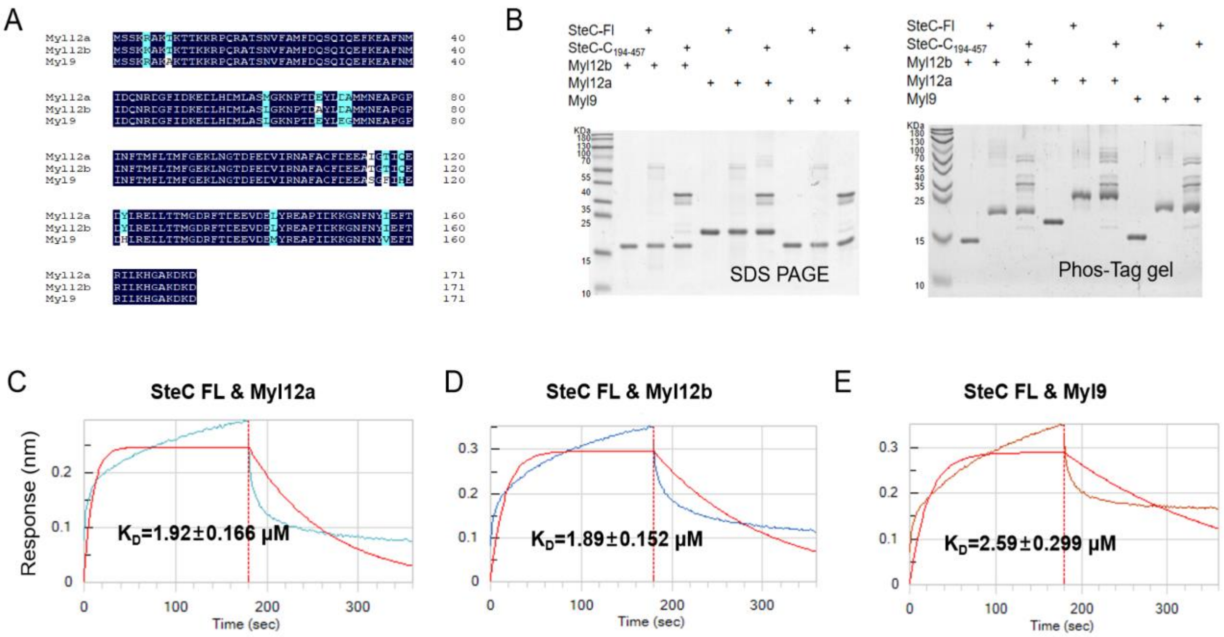

Appendix Fig.S2. SteC binds and phosporylates Myl12a homologous proteins Myl12b and Myl9, related to Fig.4 and Fig.5.

(**A**) Sequence alignment of Myl12a, Myl12b and Myl9. (**B**) *In vitro* phosphorylation assays of Myl12a, Myl12b, and Myl9 catalyzed by SteC and assayed Phos-tag gel. (**C-E**) The binding affinities of SteC FL and Myl12a, Myl12b, or Myl9 were measured by BLI, respectively. Ligand: SteC FL, Analyte: Myl12a, Myl12b, or Myl9.

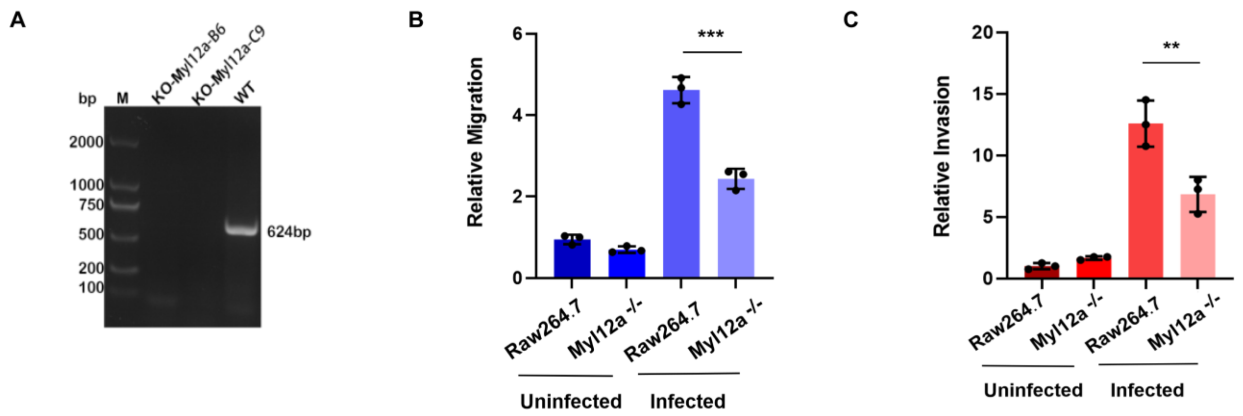

Appendix Fig.S3. The *Salmonella*-induced motility and invasive phenotypes in *myl12a* knockout RAW264.7 cells, related to Fig.4 and Fig.5.

(**A**) PCR identification for positive clones of *myl12a* knockout cell clones. (**B-C**) The migration and invasion of wild-type and Myl12a knockout RAW264.7 cells challenged by wild-type *Salmonella*. MOI=10. The migration and invasion of uninfected wild-type RAW264.7 cells were set as 1. An unpaired t test was used to determine statistical significance between two groups. **P<0.01, ***P < 0.001.

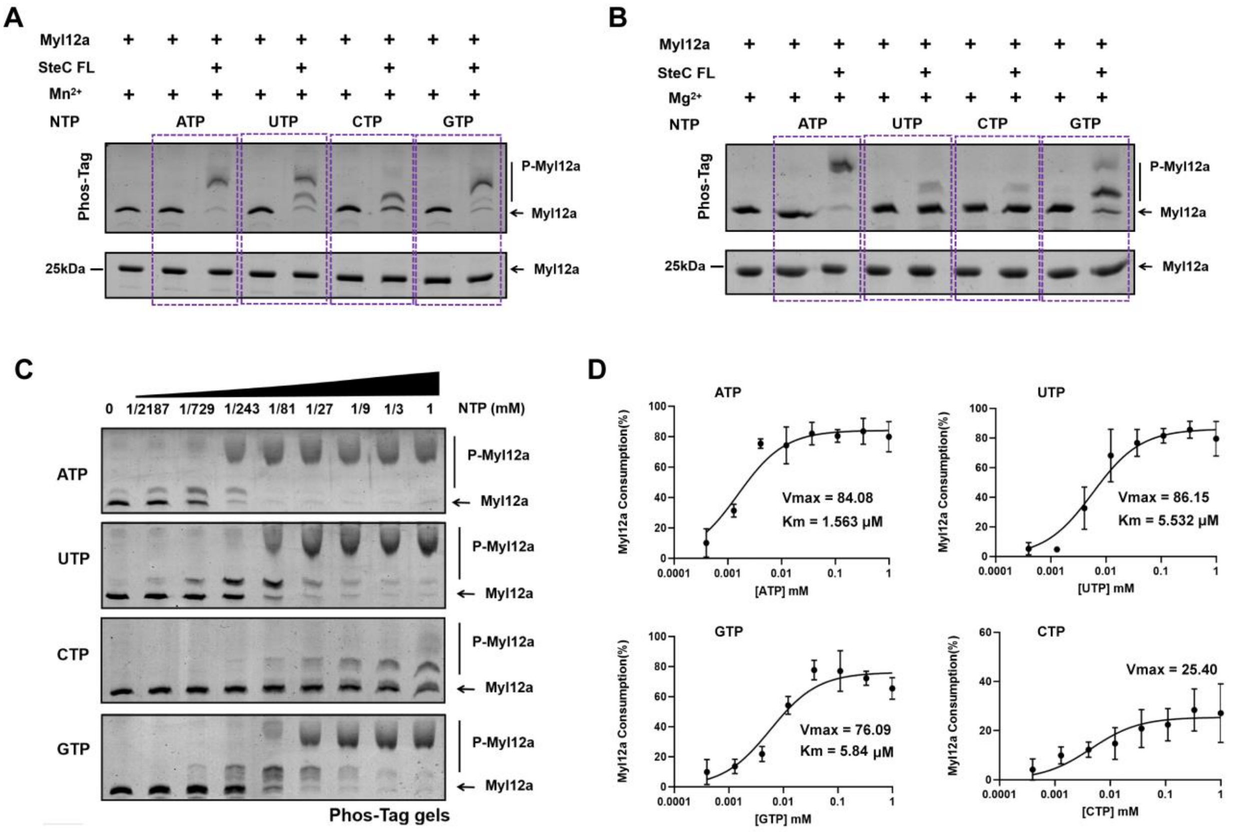

Appendix Fig.S4. SteC phosphorylates Myl12a using ATP, UTP, CTP, and GTP as the phosphate donors, related to Fig.5.

(**A-B**) *In vitro* phosphorylation assays were performed with the indicated nucleoside triphosphate and metal ion. The samples were analyzed using Phos-tag gel. P-Myl12a: Phosphorylated Myl12a. (**C**) Representative results of *in vitro* phosphorylation assays using NTP at the indicated concentration and 10 mM $MnCl_2$. The samples were analyzed using Phos-tag gels. P-Myl12a: Phosphorylated Myl12a. (**D**) Kinetic analysis of Myl12a phosphorylation using the indicated NTPs. The kinetic parameters Km were determined by the rate of Myl12a consumption. Each experiment was repeated at least three times.

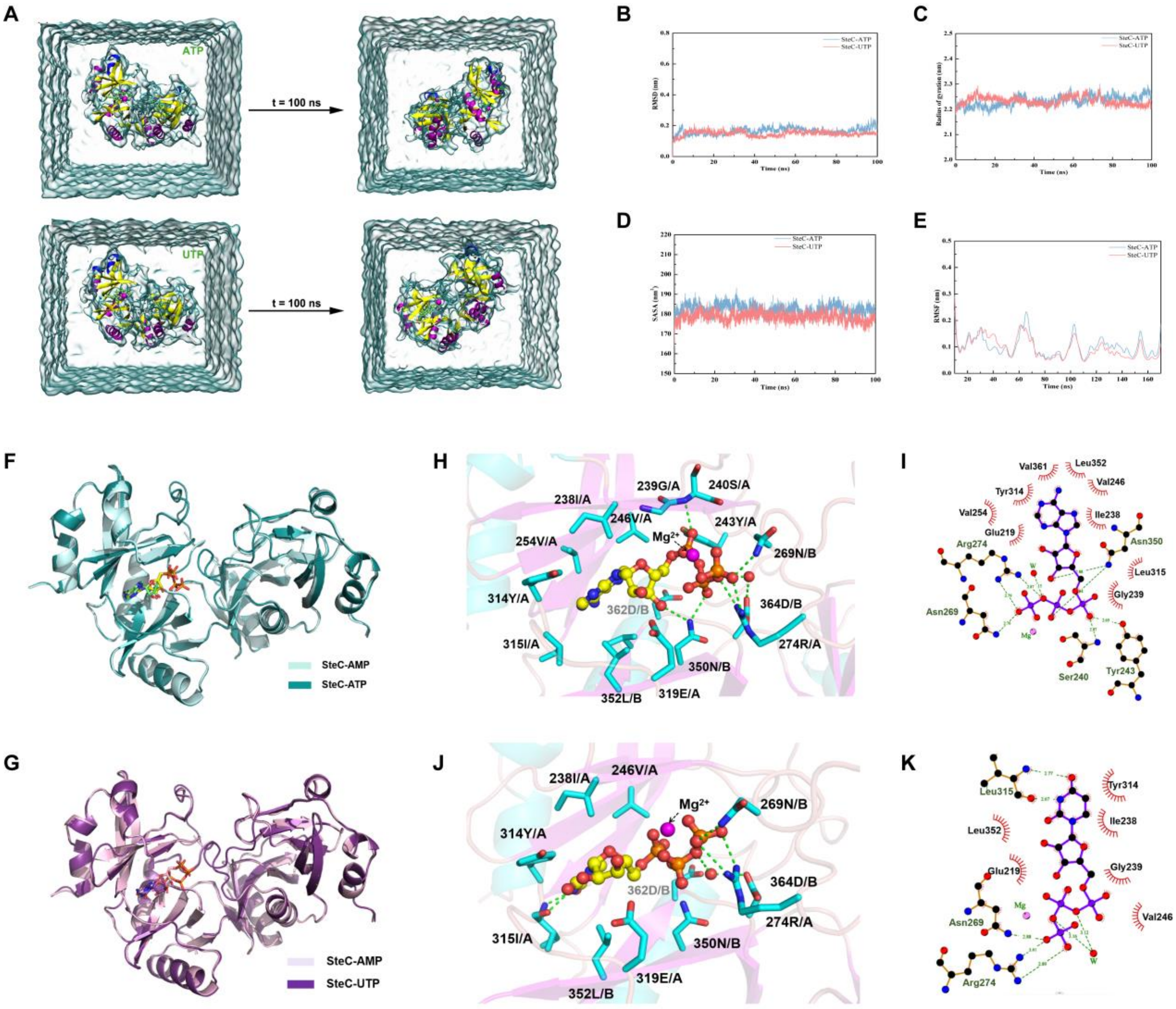

Appendix Fig.S5. Models of SteC-ATP and SteC-UTP complexes obtained through molecular dynamics simulations, related to Fig.6.

(**A**) The results of molecular dynamics simulations of SteC-ATP and SteC-UTP. (**B**-**C**) Changes in RMSD values (B) and radius of gyration (C) in SteC-ATP and SteC-UTP systems during simulation. (**D**) Changes in the solvent accessible surface area in SteC-ATP and SteC-UTP systems during simulation. (**E**) Distribution of RMSF in SteC-ATP and SteC-UTP systems during simulation. (**F**-**G**) Superposition models of SteC-ATP and SteC-UTP to SteC-AMP. (**H**-**I**) The interaction network between SteC and ATP. (**J**-**K**) The interaction network between SteC and UTP.

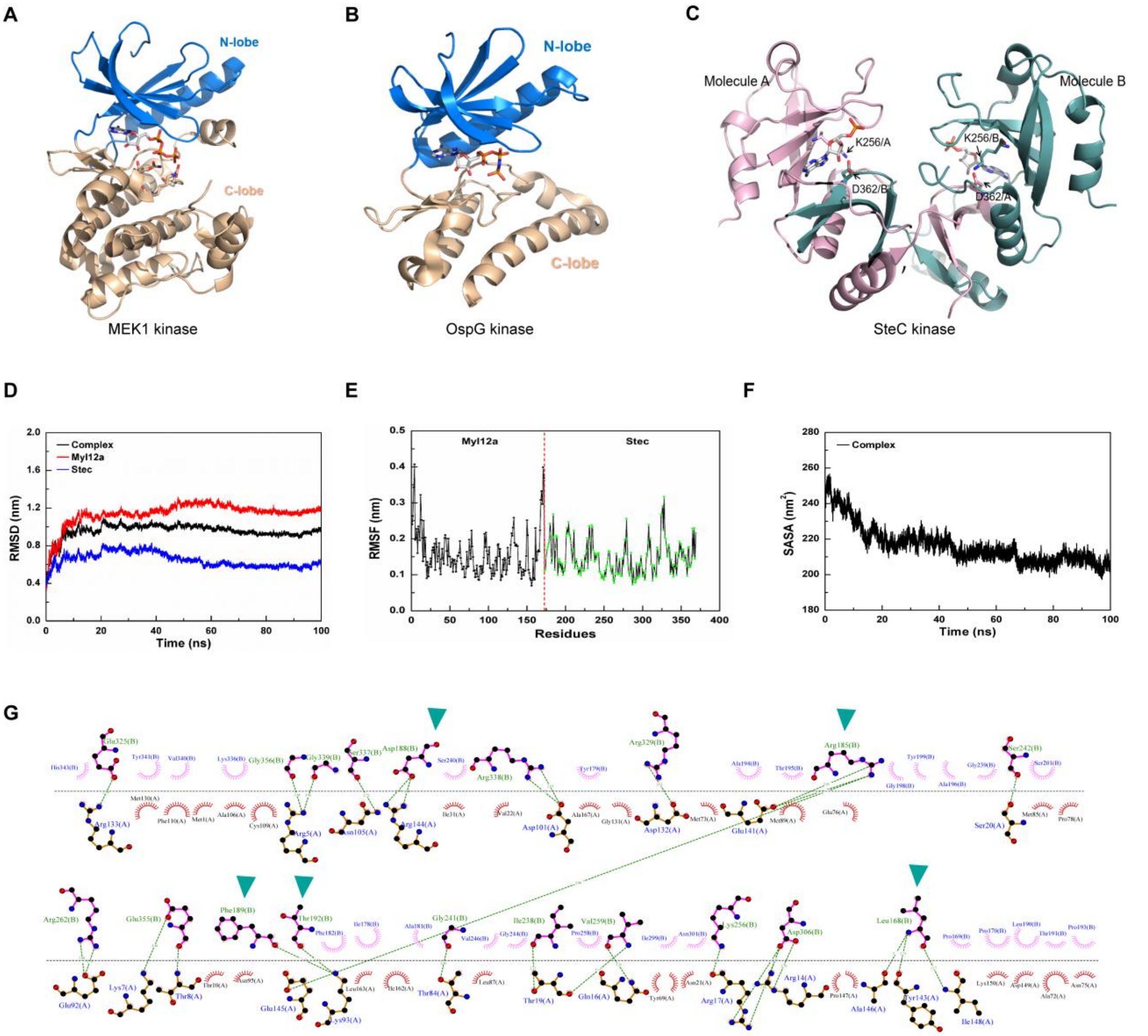

Appendix Fig.S6. Comparison of SteC with traditional kinase and SteC-Myl12a complex model obtained through molecular dynamics simulations, related to Fig.6.

(**A**) The structure of MEK1 kinase (PDBid:3DY7). MEK1 kinase is shown as a cartoon diagram and the ATP ligand is shown in stick model. (**B**) The structure of OspG kinase (PDBid:4Q5H), an example of a traditional kinase. OspG kinase is shown as a cartoon diagram and the ATP ligand is shown in stick model. (**C**) The structure of SteC kinase (PDBid:8JBI). SteC dimer is shown as a cartoon diagram and the AMP ligand and indicated residues are shown in stick model. (**D**-**F**) Changes in RMSD values (D), radius of gyration (E) and solvent accessible surface area (F) in SteC-Myl12a model during simulation. (**G**) The interaction network between SteC and Myl12a. The residues of the M-helix are indicated by purple triangles.

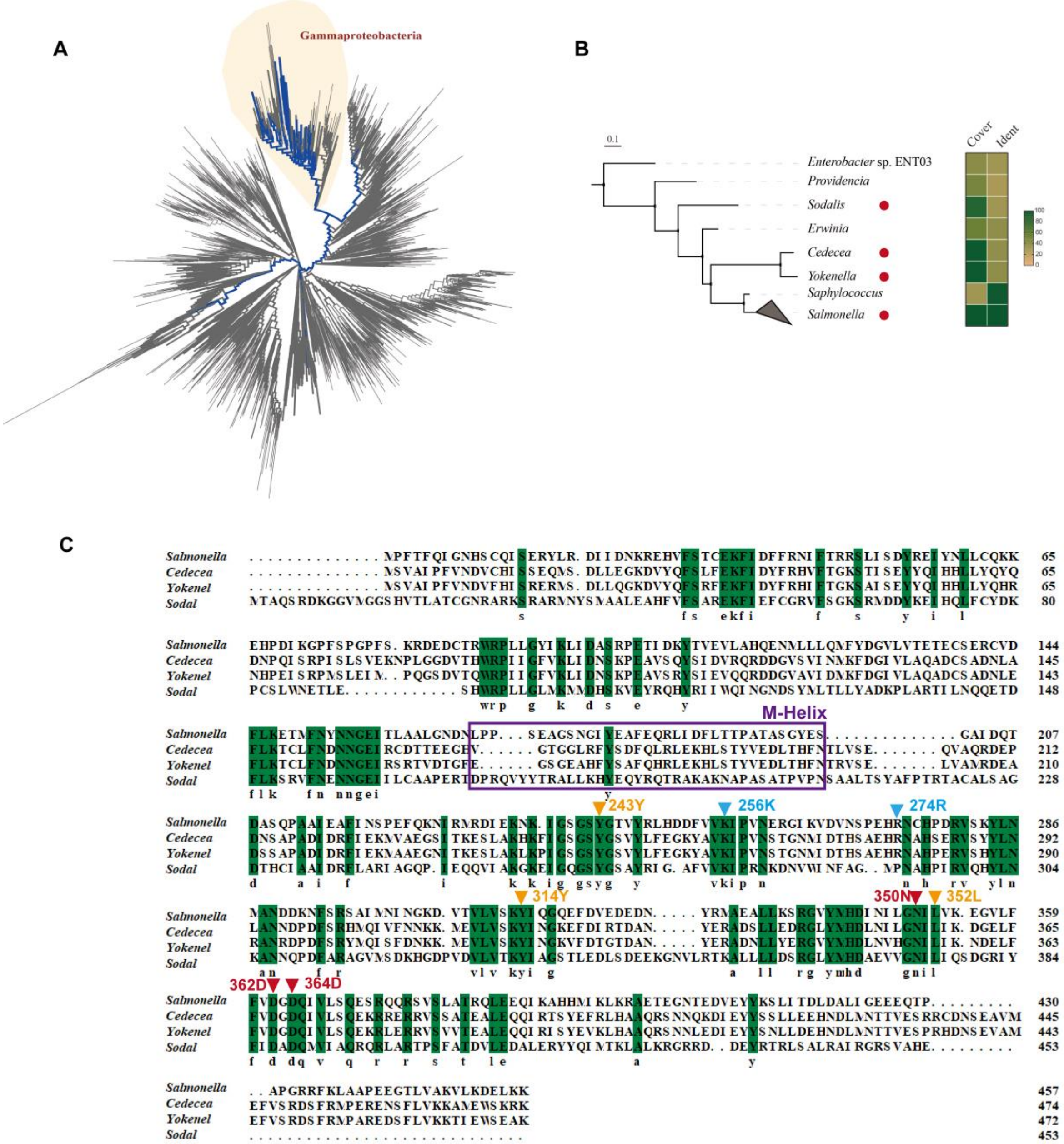

Appendix Fig.S7. Amino acid comparison and evolutionary tree of SteC orthologs, related to Fig.6.

(**A**) Evolutionary trees of SteC-like proteins (n=1000). (**B**) The relationship and evolutionary history of SteC orthologs from the indicated species. (**C**) Multispecies alignments of SteC orthologs identified in B. *Salmonella typhimurium*: Q8ZP57; *Cedecea sp. P7760*: A0A7Y7YR13; *Yokenella regensburgei*: G9Z956; and *Sodalis glossinidius*: A0A193QJ96. The essential catalytic residues and M-Helix region of SteC are highlighted.

Appendix Table S1. Proteomic analysis of differentially phosphorylated proteins in pSteC and pGFP.

Protein accession	Protein name	Position	Amino acid	pSteC/pGFP Ratio	pSteC/pGFP P value
Q8BLR5	Psd4	442	S	11.62019497	1.44162E-05
Q9Z248	Aebp2	15	S	8.83576436	0.036819613
Q62093	Srsf2	101	S	6.895244481	0.034088694
A2ADZ8	Iqcc	85	S	5.958783463	0.009977106
**Q3THE2**	**Myl12b**	**19**	**T**	**5.896250435**	**0.000184723**
Q05D44	Eif5b	191	S	5.850350812	0.000526092
B2RR83	Ythdc2	1236	S	5.747771704	4.82923E-06
Q58A65	Spag9	185	S	4.954041472	0.027271261
Q80U72	Scrib	1561	S	4.766144839	0.004389589
E9QAG8	Znf431	155	T	4.608031399	0.02491324
Q9EQG3	Scel	171	S	4.530822373	0.016527362
Q8JZQ9	Eif3b	75	S	4.457051376	0.008766207
Q5SUF2	Luc7l3	425	S	4.43262367	0.003892525
Q9D083	Spc24	181	S	4.364998867	0.007206814
Q80YV2	Zc3hc1	337	S	4.342203248	0.004693756
Q9CWT6	Ddx28	102	S	4.014091838	0.002809422
Q9WTP2	Spry4	126	S	4.012051588	1.51131E-06
B2RXS4	Plxnb2	1248	S	3.946215755	0.001172954
Q3UW53	Niban1	583	T	3.927623993	0.044768345
Q8BGN6	Prrg4	202	S	3.813195492	0.042337822
P52651	Rhox5	22	S	3.724578042	0.000513536
Q91Z46	Dusp7	372	S	3.695009719	0.025265556
Q9DBB1	Dusp6	331	S	3.695009719	0.025265556
Q9Z277	Baz1b	382	S	3.684630971	0.001418709
P23242	Gja1	255	S	3.583073376	0.005763592
Q60876	Eif4ebp1	64	S	3.465284061	0.004711116
O88939	Zbtb7a	399	T	3.454452582	0.003345055
Q80U72	Scrib	1563	S	3.449828414	0.004132204
Q9CWK8	Snx2	97	S	3.398149151	0.006621556
Q8BG81	Poldip3	206	S	3.325173989	0.001319751
Q8C8R3	Ank2	3764	S	3.153124535	0.012013042
P49312	Hnrnpa1	316	S	3.147505221	0.00027871
Q8K4P0	Wdr33	7	S	3.044580238	0.000678647
O08796	Eef2k	469	S	2.99950824	0.025230332
P15702	Spn	347	S	2.969909533	3.36759E-05
Q8C142	Ldlrap1	16	S	2.965462836	0.000305603
Q8R4H2	Arhgef12	608	S	2.950313461	0.046155911
Q9JKZ2	Slc5a3	559	S	2.9431509	0.015802589
Q62018	Ctr9	941	S	2.884909506	0.02722459

Q62018	Ctr9	943	S	2.884909506	0.02722459
Q61043	Nin	269	S	2.87996052	0.014488222
Q3U2S4	Otud5	64	S	2.811613323	8.61026E-07
P06537	Nr3c1	243	S	2.789169005	0.001228573
Q9CXW4	Rpl11	140	S	2.780754944	0.000610599
Q9DBR7	Ppp1r12a	542	S	2.738629677	0.001381875
Q80YV2	Zc3hc1	340	S	2.711256134	0.025180093
P60469	Ppfia3	1164	S	2.701790996	0.001643841
Q9Z2H5	Epb41l1	510	S	2.650206211	0.011378952
O55137	Acot1	56	S	2.594367733	0.001018264
Q8BL74	Gtf3c2	214	S	2.575946288	0.02759618
Q3U2S4	Otud5	503	S	2.520651727	0.000833556
Q9CX86	Hnrnpa0	282	S	2.501457434	1.60316E-05
Q7TNV0	Dek	76	S	2.491686594	0.001816989
Q8BHE0	Prr11	315	S	2.484996033	0.032480824
Q6GQX2	Nckap5l	494	S	2.465934472	9.2005E-05
Q60749	Khdrbs1	18	S	2.455718021	0.023539467
Q91VE6	Nifk	144	S	2.427727701	0.002003779
Q02242	Pdcd1	267	S	2.427606559	0.001569276
Q8CJF7	Ahctf1	1138	S	2.411922583	0.042061926
P18608	Hmgn1	24	S	2.397023327	0.003428868
Q99K95	Rtf2	288	S	2.386807611	0.000159303
P62869	Elob	84	T	2.383973539	0.027199119
Q9D1J3	Sarnp	165	S	2.38070134	0.042088616
A2AWP8	Arhgef10l	279	S	2.338980512	0.002453395
Q14AX6	Cdk12	384	S	2.328923878	0.049689953
Q9Z1T5	Deaf1	48	S	2.288578973	0.006840273
Q9Z1T5	Deaf1	54	S	2.288578973	0.006840273
Q8BG95	Ppp1r12b	833	S	2.285448021	0.008267241
Q9Z0G0	Gipc1	225	S	2.273503622	0.005830284
Q62441	Tle4	292	S	2.262903755	0.024667369
Q61937	Npm1	10	S	2.250015637	0.023179092
P53564	Cux1	1496	S	2.248289861	0.004377886
Q7M6Y3	Picalm	16	S	2.236901079	7.20662E-05
O08796	Eef2k	365	S	2.235034936	3.84814E-05
Q3UHX0	Nol8	819	S	2.218456947	9.45811E-06
Q3UHX0	Nol8	820	S	2.218456947	9.45811E-06
Q3UHX0	Nol8	825	S	2.218456947	9.45811E-06
Q00560	Il6st	665	S	2.206604173	0.019515846
O35857	Timm44	80	S	2.197543086	0.003932601
Q3UZA1	Rcsd1	105	S	2.194775599	0.048797691
Q3UZA1	Rcsd1	108	S	2.194775599	0.048797691
Q80YR9	Rbm12b1	667	S	2.17879278	0.006181034
B2RRD7	Brpf1	237	S	2.177903079	0.03449444

A2AWP8	Arhgef10l	152	Y	2.158579271	0.003337592
Q6GQX2	Nckap5l	436	S	2.155875242	0.008091657
Q64337	Sqstm1	207	S	2.151727716	0.023039073
Q80Y44	Ddx10	587	T	2.143984739	0.045810905
P58462	Foxp1	318	S	2.141526102	0.004447971
Q8CFH6	Sik2	343	S	2.1359283	0.008406106
Q9JKZ2	Slc5a3	557	S	2.131394592	0.000258937
Q9EST3	Eif4enif1	76	S	2.130911398	0.010825315
Q9EQG3	Scel	343	S	2.125981238	0.000182054
Q9D0E1	Hnrnpm	636	S	2.123439478	0.000502162
Q9JIY0	Plekho1	270	S	2.118682739	0.000193368
P31324	Prkar2b	112	S	2.110363328	0.000429277
P80317	Cct6a	205	S	2.106191357	0.004036275
Q8BRB7	Kat6b	1136	S	2.095177715	0.000307041
Q8BRB7	Kat6b	1140	S	2.095177715	0.000307041
Q8K3A9	Mepce	126	S	2.091423065	0.000791597
P55200	Kmt2a	3510	S	2.090255398	0.006168661
O08648	Map3k4	451	T	2.084457039	0.005511585
Q9ERK4	Cse1l	931	S	2.073053348	0.037528459
A2AWP8	Arhgef10l	268	S	2.072180483	2.94172E-05
P97287	Mcl1	81	S	2.072130177	0.022182252
Q925J9	Med1	774	S	2.069567759	0.011245097
Q80X82	Sympk	494	S	2.068879261	0.02198712
Q8BRB7	Kat6b	1302	S	2.066697992	0.007618723
P32067	Ssb	342	S	2.064857451	1.89523E-05
Q6GQX2	Nckap5l	168	T	2.058611197	0.038005884
Q3TFK5	Gpatch4	236	S	2.057784819	0.000708468
Q8K273	Mmgt1	120	S	2.057771087	0.040718575
Q9Z2S7	Tsc22d3	125	T	2.053965374	0.040323634
Q569Z5	Ddx46	804	S	2.046303674	0.03173367
Q8BJS4	Sun2	281	S	2.044882845	9.5642E-05
P49312	Hnrnpa1	95	S	2.039282944	0.000100133
O88502	Pde8a	452	S	2.037524157	0.001265828
P20491	Fcer1g	75	T	2.028347533	0.011741969
E9Q6B2	Ccdc85c	247	S	2.021338084	0.020316005
Q3TUF7	Yeats2	406	T	2.018038984	0.002246964
Q7TSH3	Znf516	972	S	2.017986809	0.036339087
Q9R210	Tfeb	113	S	2.014086755	0.033276465
A2A7S8	Kiaa1522	838	S	2.006705135	0.034440136
Q01063	Pde4d	654	S	0.497980432	0.016503527
Q5SSZ5	Tns3	1149	S	0.493975282	0.00836096
Q6ZQ58	Larp1	743	S	0.492118881	8.76197E-05
Q6ZQH8	Nup188	69	S	0.48894193	0.029766563
Q9D281	Fam114a1	197	T	0.484047582	0.000138632
Q3TWF6	Wdr70	641	S	0.480004566	0.00030109

Q9CXV0	Isl2	279	S	0.479236486	0.006333151
Q6PNC0	Dmxl1	1965	S	0.478497907	0.003065915
Q6ZQ58	Larp1	302	S	0.478056704	0.023608594
Q8CHG7	Rapgef2	1224	S	0.476307691	0.016787658
Q6Y5D8	Arhgap10	715	S	0.473052636	0.014449891
Q91VI7	Rnh1	2	S	0.468336256	0.00216588
Q6ZQ58	Larp1	498	S	0.467861922	2.9619E-06
Q9QY06	Myo9b	1218	S	0.464117702	0.043366638
E9Q612	Ptpro	875	S	0.460543399	0.003499687
P13405	Rb1	814	T	0.460050934	0.008004239
Q6Y5D8	Arhgap10	591	S	0.458688466	1.32782E-07
Q925J9	Med1	1530	S	0.457378051	0.006715842
Q6ZQ58	Larp1	503	T	0.447943083	0.000346879
Q99N13	Hdac9	22	S	0.44552615	0.004082074
Q02053	Uba1	377	S	0.444516741	0.00263805
P19973	Lsp1	243	S	0.438967272	4.6343E-05
O35601	Fyb1	561	S	0.435782404	0.027776312
Q99LJ0	Cttnbp2nl	481	S	0.434436246	0.035846116
Q9QUN3	Blnk	129	S	0.43220057	0.000555587
Q8BGD9	Eif4b	597	S	0.409044687	0.025033162
O70439	Stx7	45	S	0.406264094	0.003062715
P49769	Psen1	365	S	0.394956644	0.03036395
Q8BS90	Bora	137	S	0.393168496	0.004080259
Q6PDK2	Kmt2d	1822	T	0.369098897	0.021148143
O35984	Pbx2	330	S	0.367628612	0.00854749
Q6ZWR6	Syne1	8308	S	0.359892599	0.002021782
Q8C1S0	Med19	194	S	0.346732682	0.046116956
Q5SF07	Igf2bp2	164	S	0.346303248	0.015981612
Q78ZA7	Nap1l4	51	T	0.339297534	0.005710209
Q8BH50	--	66	S	0.338423564	0.002333946
Q571I4	Prag1	671	S	0.335324233	0.01970229
Q640M1	Utp14a	182	T	0.333557185	0.009982496
O35623	Bet1	50	S	0.327270511	0.001713138
Q62407	Speg	2114	S	0.326654839	0.01319819
Q80W04	Tmcc2	435	S	0.3039847	0.00125231
Q8R310	Tmcc3	216	S	0.3039847	0.00125231
Q6PDL0	Dync1li2	194	S	0.303530033	0.000568077
P56959	Fus	333	S	0.284415488	0.049158291
Q5DU09	Znf652	203	S	0.280168684	0.010103545
Q9DBC3	Cmtr1	52	S	0.277336175	0.00022265
O54957	Lat	199	S	0.270400958	3.2869E-06
O54957	Lat	87	S	0.250135931	5.16415E-06
Q6PDK2	Kmt2d	2318	S	0.232587482	0.00095161
Q6A065	Cep170	872	S	0.22192198	0.033969649

Q9EPA7	Nmnat1	137	S	0.201774107	4.82137E-05
P00920	Ca2	2	S	0.19500511	7.17544E-05
O88351	Ikbkb	697	S	0.172935899	0.013743158
Q6PR54	Rif1	1540	S	0.160609095	0.002037598

Appendix Table S2. Data collection and refinement statistics

Stucture	Se-Met-SteC-AMP-Mg^{2+}
PDB ID	8JBI
Space group	*P1 2_1 1*
a, *b*, *c* (Å)	57.00, 92.88, 88.84
α, β, γ (º)	90.0, 107.82, 90.0
Wavelength (Å)	0.9791
Resolution (Å)	40.40-2.36(2.43-2.36)*
⟨ *I*/σ(*I*)⟩	24.3(2.9)
Completeness (%)	96.6(99.1)
Redundancy	11.4(8.4)
$CC_{1/2}$	0.999（0.896）
R_{pim}	0.022（0.246）
No. reflections	35432
R_{work} / R_{free} (%)	24.2/27.0
Protein	5158
Ion	5 Mg^{2+}
Ligands	4 AMP
Water	167
Bond lengths (Å)	0.01
Bond angles (°)	1.33

*values in parentheses are for highest-resolution shell.

Appendix Table S3.Hydrogen bonds identified in SteC-Myl12a model

No.	SteC	Myl12a	Distance(nm)
1	Glu325-OE1	Arg133-NH2	0.278
2	Gly356-O	Arg5-NH2	0.284
3	Gly339-O	Arg5-NH2	0.306
4	Ser337-O	Asn105-ND2	0.272
5	**Asp188-OD1**	Arg144-NH1	0.290
6	**Asp188-OD1**	Arg144-NH2	0.289
7	Arg388-NH1	Asp101-OD2	0.263
8	Arg388-NH2	Asp101-OD2	0.277
9	Arg329-NH2	Asp132-OD1	0.263
10	**Arg185-NH1**	Glu141-OE1	0.283
11	**Arg185-NH2**	Glu141-OE1	0.277
12	**Arg185-NH2**	Glu145-OE2	0.288
13	Ser242-O	Ser20-O	0.265
14	Arg262-NH1	Glu92-OE2	0.272
15	Arg262-NH2	Glu92-OE2	0.288
16	Glu355-OE2	Lys7-NZ	0.284
17	Glu355-O	Thr8-N	0.277
18	**Phe189-O**	Lys93-NZ	0.269
19	**Thr192-O**	Lys93-NZ	0.273
20	Gly241-O	Thr84-OG1	0.286
21	Ile238-O	Thr19-OG1	0.273
22	Val259-N	Thr19-O	0.305
23	Val259-O	Gln16-NE2	0.320
24	Lys256-NZ	Arg17-O	0.276
25	Asp306-OD2	Arg17-NH1	0.282
26	Asp306-OD2	Arg17-NH2	0.283
27	Asp306-OD1	Arg17-NE	0.276
28	**Leu168-N**	Ala146-O	0.281
29	**Leu168-N**	Tyr143-O	0.274
30	**Leu168-O**	Ile148-N	0.317

Structure-based molecular dynamics simulation was performed of SteC 168aa-364aa binding with Myl12a. The detailed information of the hydrogen bonds interaction were list. The residues from M-helix of SteC (168aa-202aa) were highlighted in blue color.

Appendix Table S4. Primers used in this study

No	Name	Sequence
1	SteC-1AA-BamHI-5	ATAGGATCCATHCCGTTTACATTTCAGATCG
2	SteC-194AA-BamHI-5	ATAGGATCCGCCACGGCGTCTGGTTATG
3	SteC-202AA-BamHI-5	ATAGGATCCGGCGCTATTGATCAGACGGAT
4	SteC-211AA-BamHI-5	ATAGGATCCCAACCAGCGGCAATA
5	SteC-364AA-XhoI-3T	ATACTCGAGTTAATCGCCATCCACAAAGAAAAG
6	SteC-375AA-XhoI-3T	ATACTCGAGTTACTGTTGCCGTGATTCTTGTGA
7	SteC-457AA-XhoI-3T	ATACTCGAGTTATTTTTTAATTCATCCTTTAATACC
8	SteC-168AA-BamHI-5	ATACATATGCTACCGCCGAGCGAGGCA
9	SteC-1AA-BamHI-5	ATAGGATCCATHCCGTTTACATTTCAGATCG
10	SteC-194AA-BamHI-5	ATAGGATCCGCCACGGCGTCTGGTTATG
11	JD-*steC*-F1	GCGTGTAGTAATGCGCGAAT
12	JD-*steC*-R1	ATTCTTCTACGCGGCCTTTC
13	JD-*ssaV*-F1	GAAACTGGAGCGCAGACAG
14	JD-*ssaV*-R1	CGCCAACGGCTCATTCATATC
15	JD-*ssaV*-F2	CAGCAGGAAAGCCGCTTTC
16	JD-*ssaV*-R2	GGTAACGTCTGGCTGTCAC
17	JD-*sseI*-F1	GTATATAGGTCCACACGGACAC
18	JD-*sseI*-R1	CGTGAAGAATATCTGGCATATCTG
19	JD-*sseI*-F2	GGGTTTGTAAATATAGAATGGCAAC
20	JD-*sseI*-R2	CAAATCTGCTGACCACATCCTC
21	SteC168-364aa I178A/Y179A/E180A-F	GCGGTATTGTTATCAGTCATGGATCCATGCTACCGCCGAGCG AGGCA
22	SteC168-364aa I178A/Y179A/E180A-R	ATGCGCACGAAGGCAAATAACTCGAGCTAATCGCCATCCACA AAGAAAAG
23	SteC168-364aa I178A/Y179A/E180A-MutF:	AGCGAGGCAGGTAGTAATGGAGCAGCTGCCGCTTTTGA
24	SteC168-364aa I178A/Y179A/E180A-MutR:	TCTATCAAACGTTGCTCAAAAGCGGCAGCTGCTCCATT
25	SteC168-364aa F182A/E183A/Q184A/R185A -F	GCGGTATTGTTATCAGTCATGGATCCATGCTACCGCCGAGCG AGGCA
26	SteC168-364aa F182A/E183A/Q184A/R185A -R	ATGCGCACGAAGGCAAATAACTCGAGCTAATCGCCATCCACA AAGAAAAG
27	SteC168-364aa F182A/E183A/Q184A/R185A -MutF	GTAGTAATGGAATCTATGAGGCTGCAGCTGCCGCGTTGAT
28	SteC168-364aa F182A/E183A/Q184A/R185A -MutR	GTGGTCAGGAAGTCTATCAACGCGGCAGCTGCAGCCT
29	SteC168-364aa L186A/I187A/D188A-F	GCGGTATTGTTATCAGTCATGGATCCATGCTACCGCCGAGCG AGGCA
30	SteC168-364aa L186A/I187A/D188A-R	ATGCGCACGAAGGCAAATAACTCGAGCTAATCGCCATCCACA AAGAAAAG
31	SteC168-364aa L186A/I187A/D188A-MutF	TCTATGAGGCTTTTGAGCAACGTGCAGCTGCCTTCCTG

32	SteC168-364aa L186A/I187A/D188A-MutR	CCGTGGCGGGCGTGGTCAGGAAGGCAGCTGCACGTTGC
33	SteC168-364aa D188A/F189A/L190A-F	GCGGTATTGTTATCAGTCATGGATCCATGCTACCGCCGAGCG AGGCA
34	SteC168-364aa D188A/F189A/L190A-R	ATGCGCACGAAGGCAAATAACTCGAGCTAATCGCCATCCACA AAGAAAAG
35	SteC168-364aa D188A/F189A/L190A-MutF	GAGGCTTTTGAGCAACGTTTGATAGCAGCTGCCACCACG
36	SteC168-364aa D188A/F189A/L190A-MutR	ACCAGACGCCGTGGCGGGCGTGGTGGCAGCTGCTATCAAAC
37	stec pEGFP-C1F	TCGAGCTCAAGCTTCGAATTCTATGCCTTTCACCTTCCAGATT GGAAACCACAGCTGCC
38	stec pEGFP-C1R	TTATCTAGATCCGGTGGATCCTCATTTCTTCAGC
39	mMyl12a pmCherry-C1F	F: AGTCCGGACTCAGATCTCGAGCTatgtctagcaaaagggcaaag
40	mMyl12a pmCherry-C1R	R: TTATCTAGATCCGGTGGATCCttagtcatctttgtctttcgc
41	Myl12a-F	GCGGTATTGTTATCAGTCATGGATCCATGAGCAGTAAACGTG CCAAAAC
42	Myl12a-R	ATGCGCACGAAGGCAAATAACTCGAGTTAATCATCTTTATCTT TTGCACC
43	Myl12b-F	GCGGTATTGTTATCAGTCATGGATCCATGAGCAGCAAAAAAG CAAAAAC
44	Myl12b-R	R:ATGCGCACGAAGGCAAATAACTCGAGTTAATCATCTTTATC TTTGGCGCC
45	Myl9-F	F:GCGGTATTGTTATCAGTCATGGATCCATGAGTAGTAAGCGT GCCAAAG
46	Myl9-R	R:ATGCGCACGAAGGCAAATAACTCGAGTTAATCATCTTTATC TTTGGCACC
47	Myl12a-KO-F	AGCCGATCCTCTGTCAGAGGAGG
48	Myl12a-KO-R	AACCGGTGAGTGATGAGCACAGG
49	Myl12a-KO-VF	AGCCAACCTGCGGAGAGTC
50	Myl12a-KO-VR	ACCCAACTGACGAATACCTGG

Appendix Table S5. Plasmids used in this study

No	Plasmids	Application	Source
1	SteC1-457aa/pGL01	Plasmids for protein expression	This study
2	SteC194-457aa/pGL01	Plasmids for protein expression	This study
3	SteC202-457aa/pGL01	Plasmids for protein expression	This study
4	SteC202-375aa/pGL01	Plasmids for protein expression	This study
5	SteC202-364aa/pGL01	Plasmids for protein expression	This study
6	SteC211-364aa/pGL01	Plasmids for protein expression	This study
7	SteC194-364aa/pGL01	Plasmids for protein expression	This study
8	SteC168-364aa/pGL01	Plasmids for protein expression	This study
9	SteC1-457aa K256H /pGL01	Plasmids for protein expression	This study
10	SteC194-457aa N350A/pGL01	Plasmids for protein expression	This study
11	SteC194-457aa D362A/pGL01	Plasmids for protein expression	This study
12	SteC194-457aa D364A/pGL01	Plasmids for protein expression	This study
13	SteC168-364aa I178A,Y179A,E180A /pGL01	Plasmids for protein expression	This study
14	SteC168-364aa F182A,E183A,Q184A,R185A /pGL01	Plasmids for protein expression	This study
15	SteC168-364aa L186A, I187A, D188A /pGL01	Plasmids for protein expression	This study
16	SteC168-364aa D188A, F189A, L190A /pGL01	Plasmids for protein expression	This study
17	Murine Myl12a /pGL01	Plasmids for protein expression	This study
18	Murine Myl12b /pGL01	Plasmids for protein expression	This study
19	Murine Myl9/pGL01	Plasmids for protein expression	This study
20	Murine Myl12a T19A, S20A /pGL01	Plasmids for protein expression	This study
21	steC/YOE-LV001	Plasmids for RAW264.6 expression	This study
22	steC K256H/YOE-LV001	Plasmids for RAW264.6 expression	This study
23	GFP/YOE-LV001	Plasmids for RAW264.6 expression	Lab strain
24	SteC/pGBKT7	Plasmids for B2H	This study
25	Myl12a/pGADT7	Plasmids for B2H	This study
26	pGBKT7-53	Positive control for B2H	Lab strain
27	pGADT7-T	Positive control for B2H	Lab strain
28	pGADT7-Lam	Negative control for B2H	Lab strain
29	pKD3	Template plasmid for frt-flanked cat cassette	Lab strain
30	pKD46	Red recombinase expression plasmid	Lab strain
31	pCP20	Thermal induction of FLP synthesis plasmid	Lab strain
32	SteC/pEGFP-C1	Plasmids for co-localization	This study
33	Myl12a/pmCherry-C1	Plasmids for co-localization	This study
34	pFPV25.1(GFP)	Plasmids for imaging	Lab strain
35	pFPV25.1(Luc)	Plasmids for imaging	Lab strain
