# Supplementary material for: Salmonella manipulates macrophage migration via SteC-mediated myosin light chain activation to penetrate the gut-vascular barrier
Source: EMBO J. 2024 Mar 25;43(8):1499–518. doi: 10.1038/s44318-024-00076-7 (PMC11021425; doi:10.1038/s44318-024-00076-7)
Supplement: Supplementary file 2 — Movie EV1 [file 44318_2024_76_MOESM2_ESM.zip › Moive EV1/Movie EV1 Legend.docx]

Movie EV1. Video recordings of RAW264.7 infected with wild-type *Salmonella*, related to Fig.2.

Migration ability of RAW 264.7 macrophages infected with GFP-labeled wild-type *Salmonella* 2 hours post infection. Time-lapse images were captured using confocal microscopy, with a frame taken every minute.
