# Supplementary material for: Salmonella manipulates macrophage migration via SteC-mediated myosin light chain activation to penetrate the gut-vascular barrier
Source: EMBO J. 2024 Mar 25;43(8):1499–518. doi: 10.1038/s44318-024-00076-7 (PMC11021425; doi:10.1038/s44318-024-00076-7)
Supplement: Supplementary file 4 — Movie EV3 [file 44318_2024_76_MOESM4_ESM.zip › Moive EV3/Movie EV3.docx]

Movie EV3. Video recordings of pSteC crossing the vascular epithelial cell barrier, related to Fig.7.

SteC-expressing RAW264.7 cells (GFP-labeled) were subsequently seeded into the Huvec cell-fixed chambers and placed onto a confocal dish for imaging with a confocal microscope. The cells inside the chamber were real-time imaged using a Zeiss LSM 980 confocal microscope.
