# Supplementary figures and images for: Salmonella manipulates macrophage migration via SteC-mediated myosin light chain activation to penetrate the gut-vascular barrier
Source: EMBO J. 2024 Mar 25;43(8):1499–518. doi: 10.1038/s44318-024-00076-7 (PMC11021425; doi:10.1038/s44318-024-00076-7)

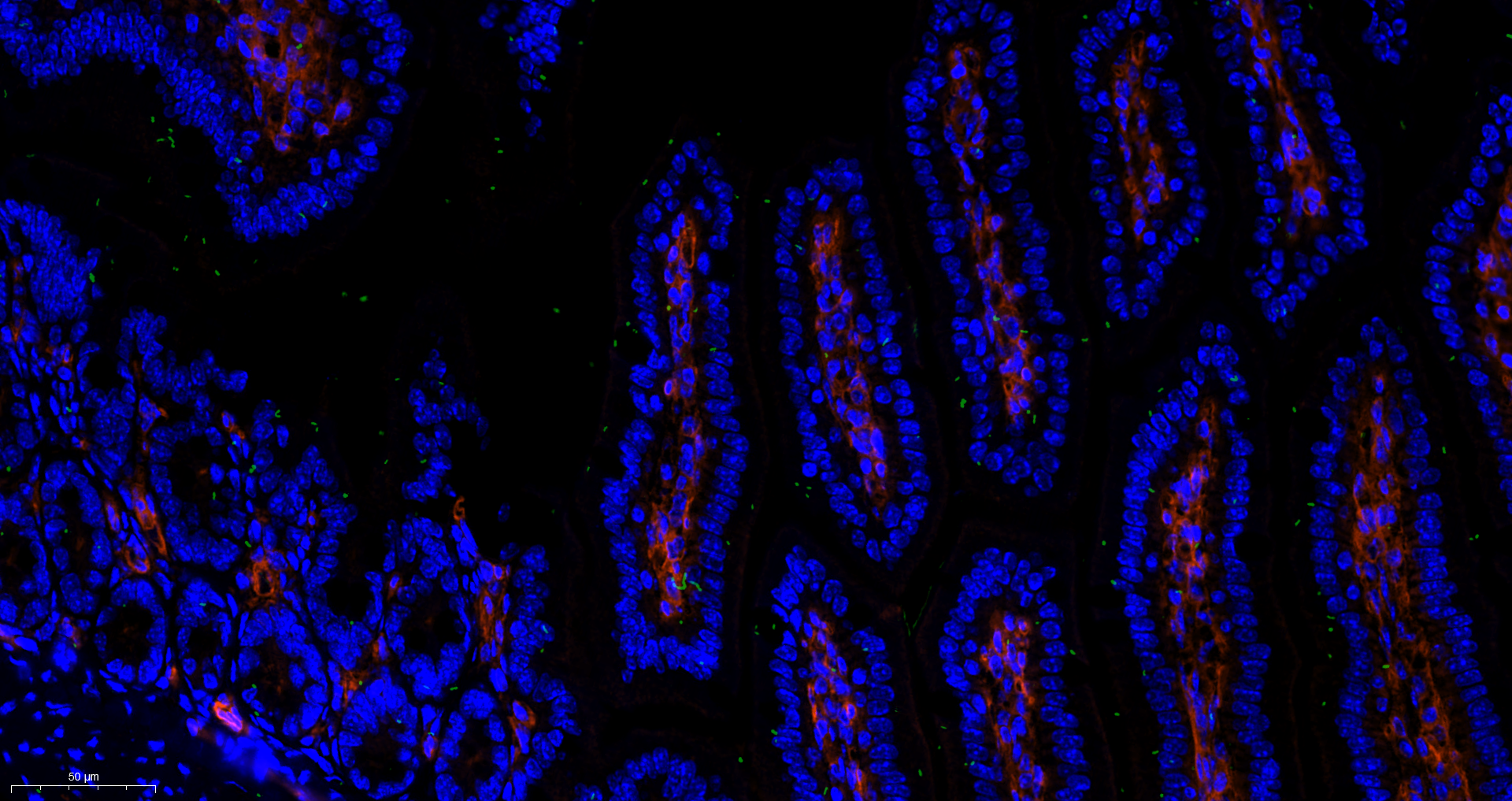

Supplement: Supplementary file 5 — Source data Fig. 1 [file 44318_2024_76_MOESM5_ESM.zip › Figure1/1A/F1A WT3-0.5h PV-1 red+Salmonella green 36.5x.tif]

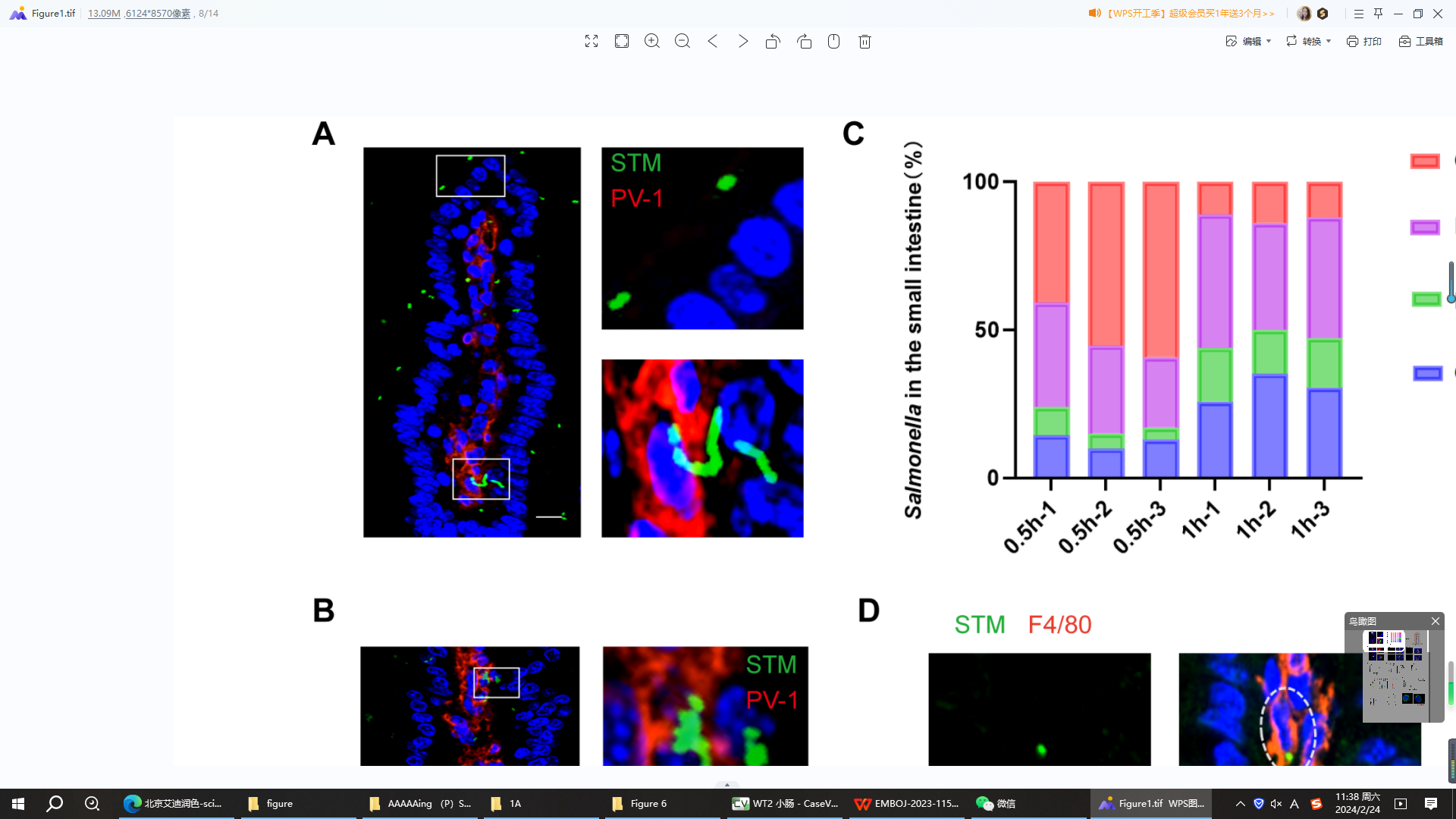


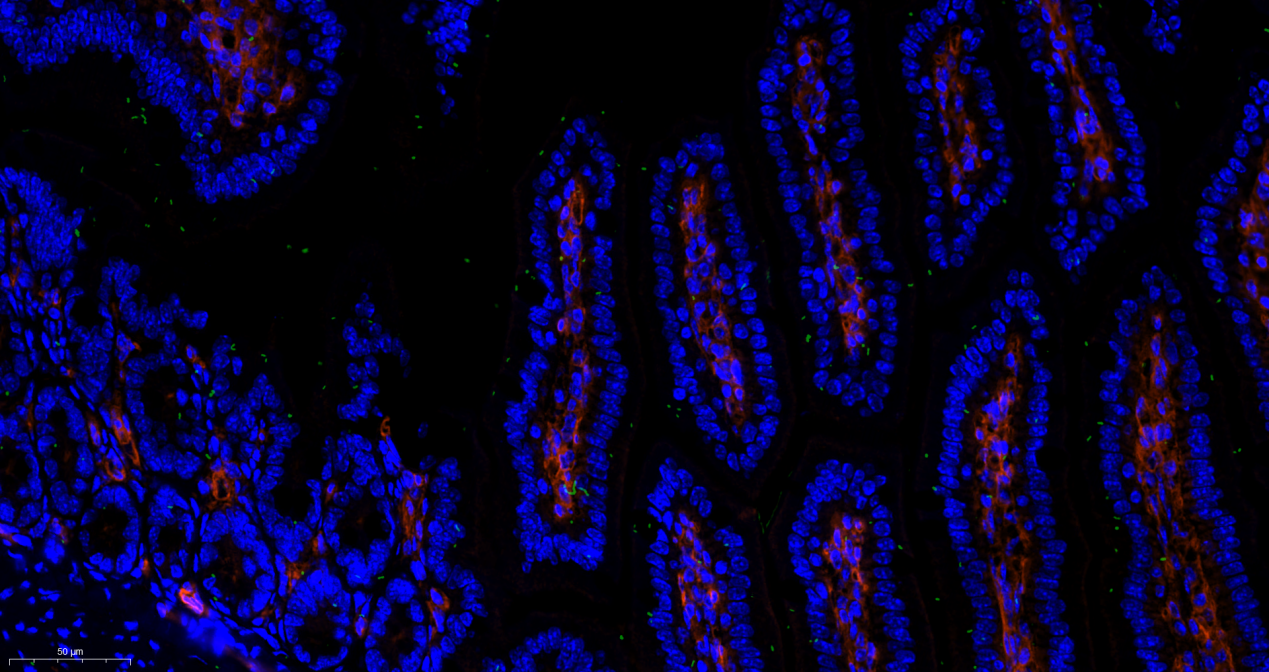

Supplement: Supplementary file 5 — Source data Fig. 1 [file 44318_2024_76_MOESM5_ESM.zip › Figure1/1A/READ ME.docx]

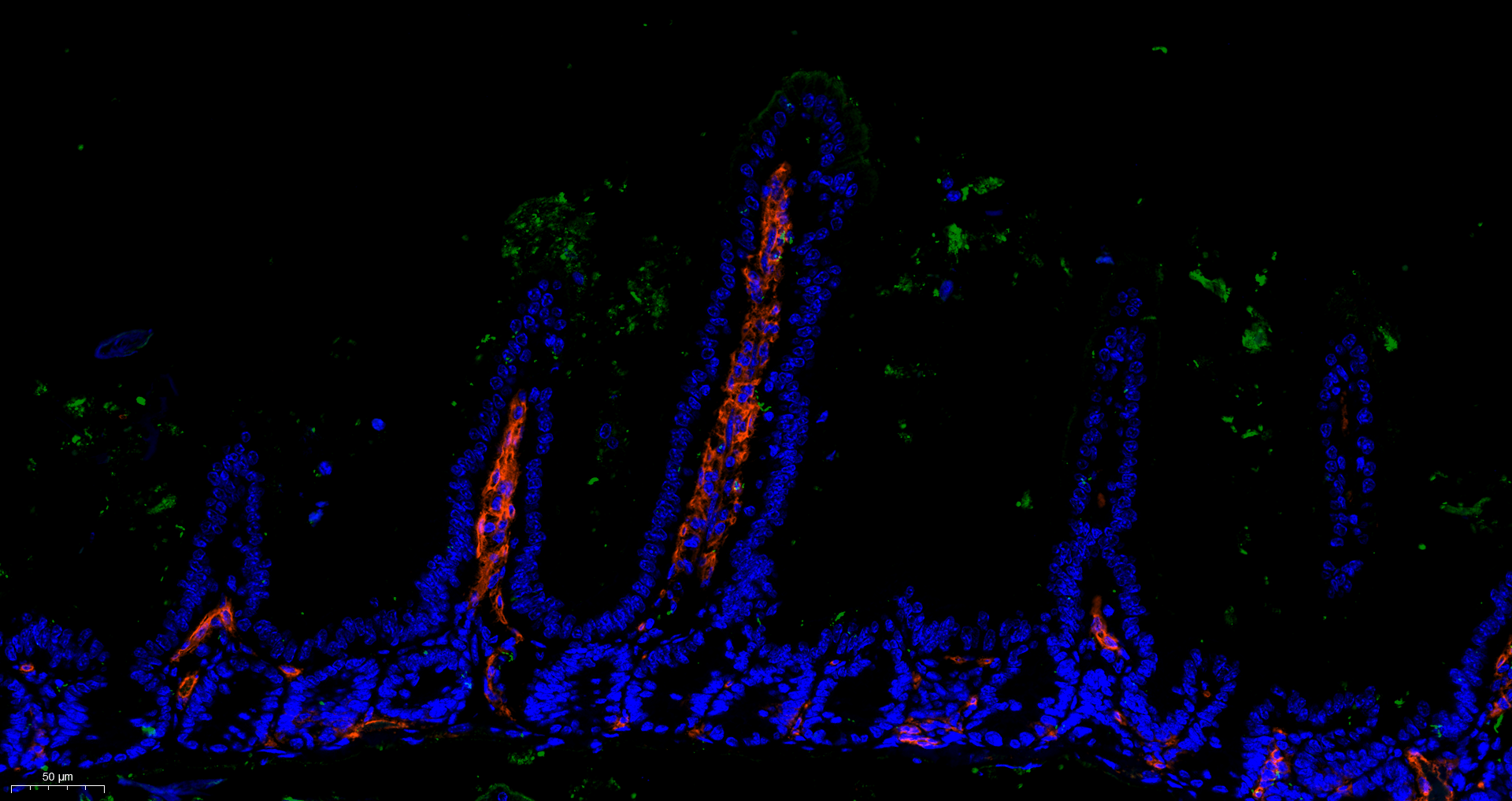

Supplement: Supplementary file 5 — Source data Fig. 1 [file 44318_2024_76_MOESM5_ESM.zip › Figure1/1B/F1B WT2 1h PV-1red+salmonella Green_23.6x.tif]

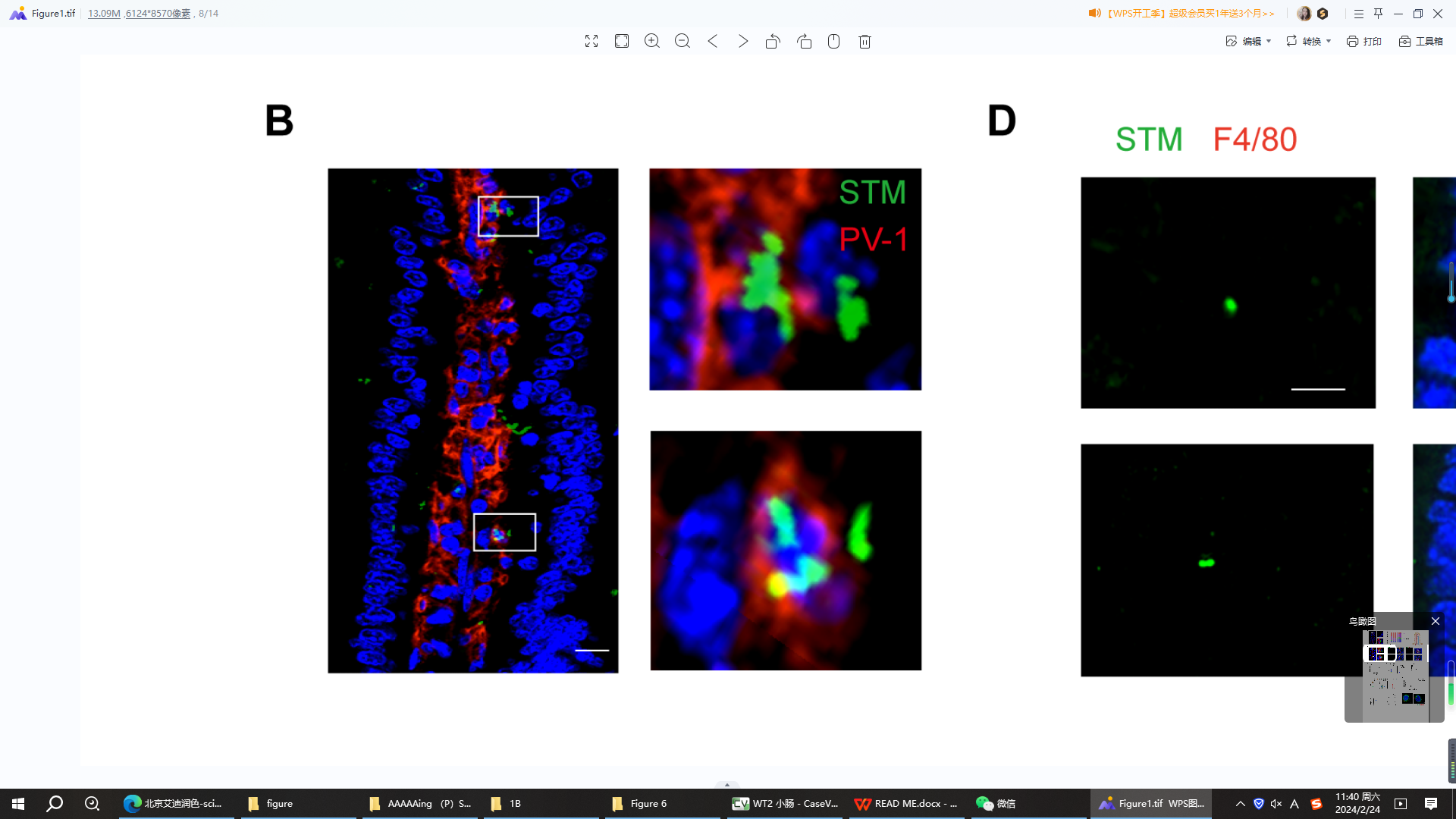


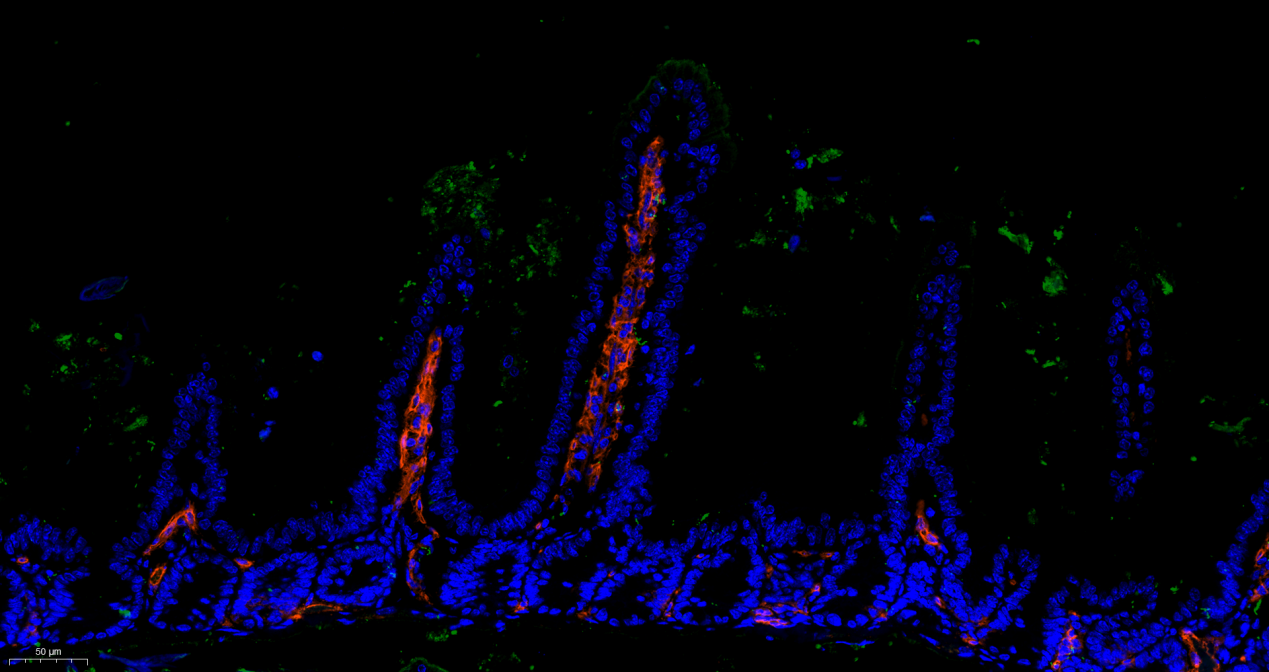

Supplement: Supplementary file 5 — Source data Fig. 1 [file 44318_2024_76_MOESM5_ESM.zip › Figure1/1B/READ ME.docx]

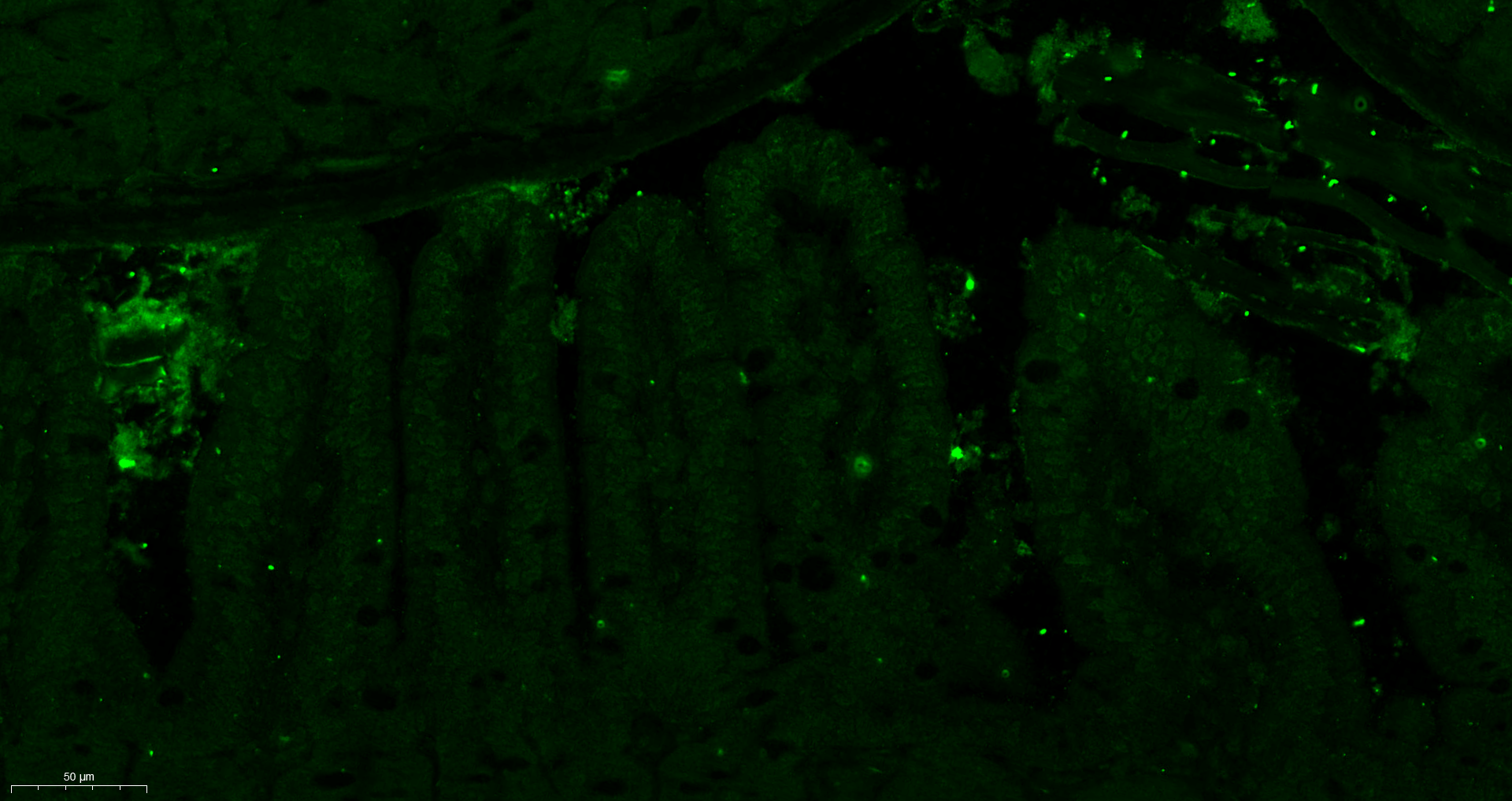

Supplement: Supplementary file 5 — Source data Fig. 1 [file 44318_2024_76_MOESM5_ESM.zip › Figure1/1D/1D-1 GREEN.tif]

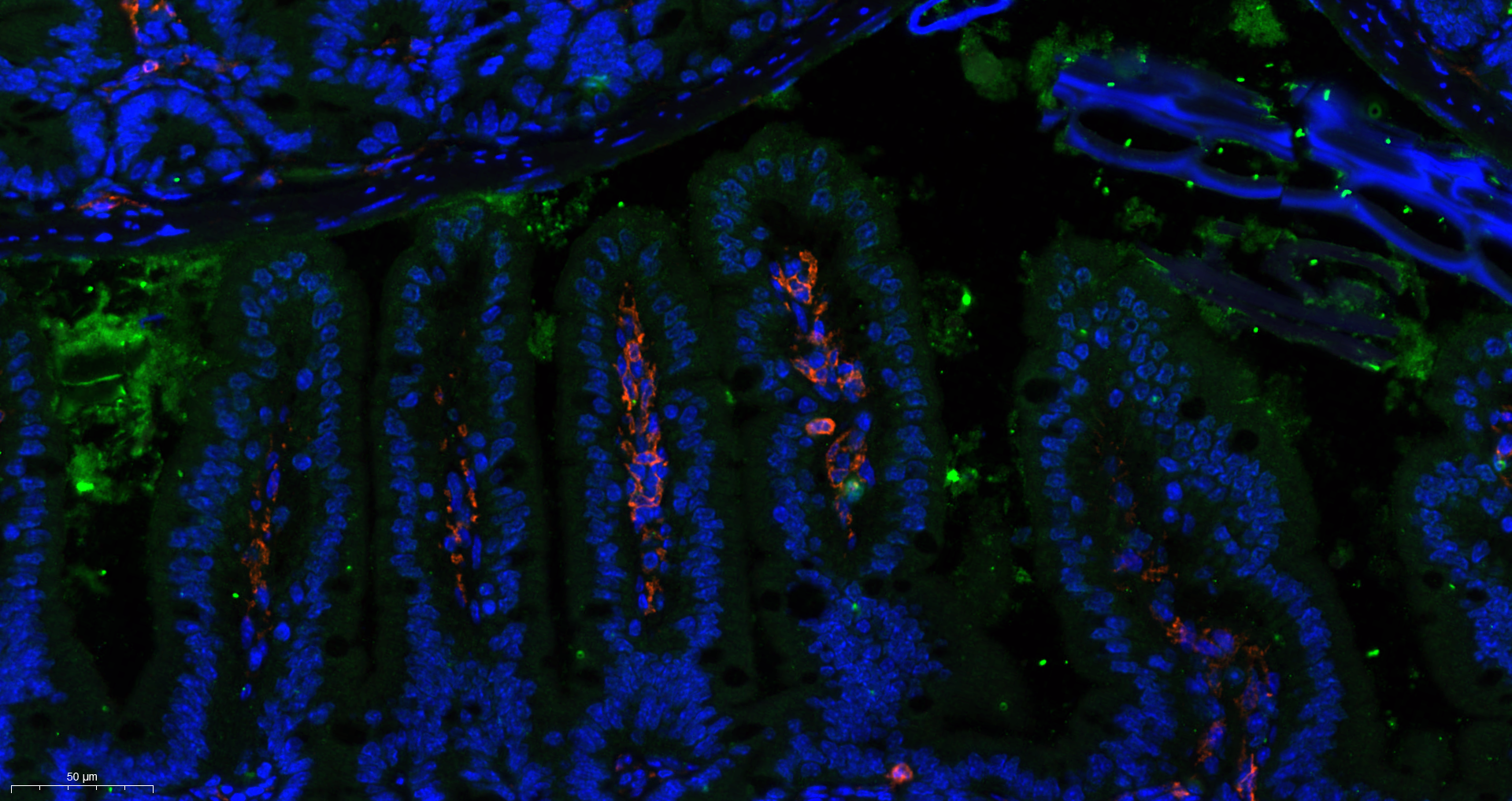

Supplement: Supplementary file 5 — Source data Fig. 1 [file 44318_2024_76_MOESM5_ESM.zip › Figure1/1D/1D-1.tif]

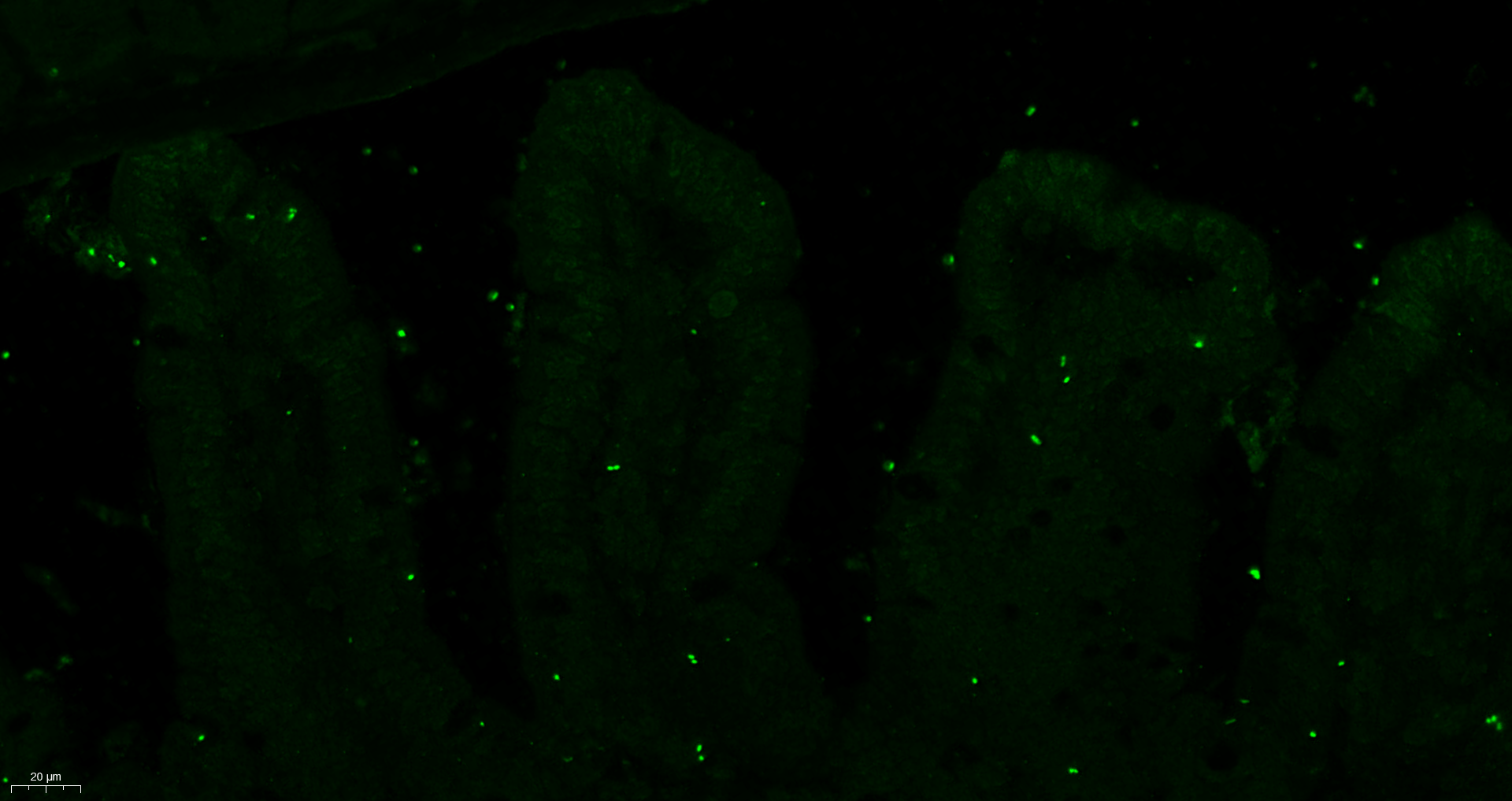

Supplement: Supplementary file 5 — Source data Fig. 1 [file 44318_2024_76_MOESM5_ESM.zip › Figure1/1D/1D-2 GREEN.tif]

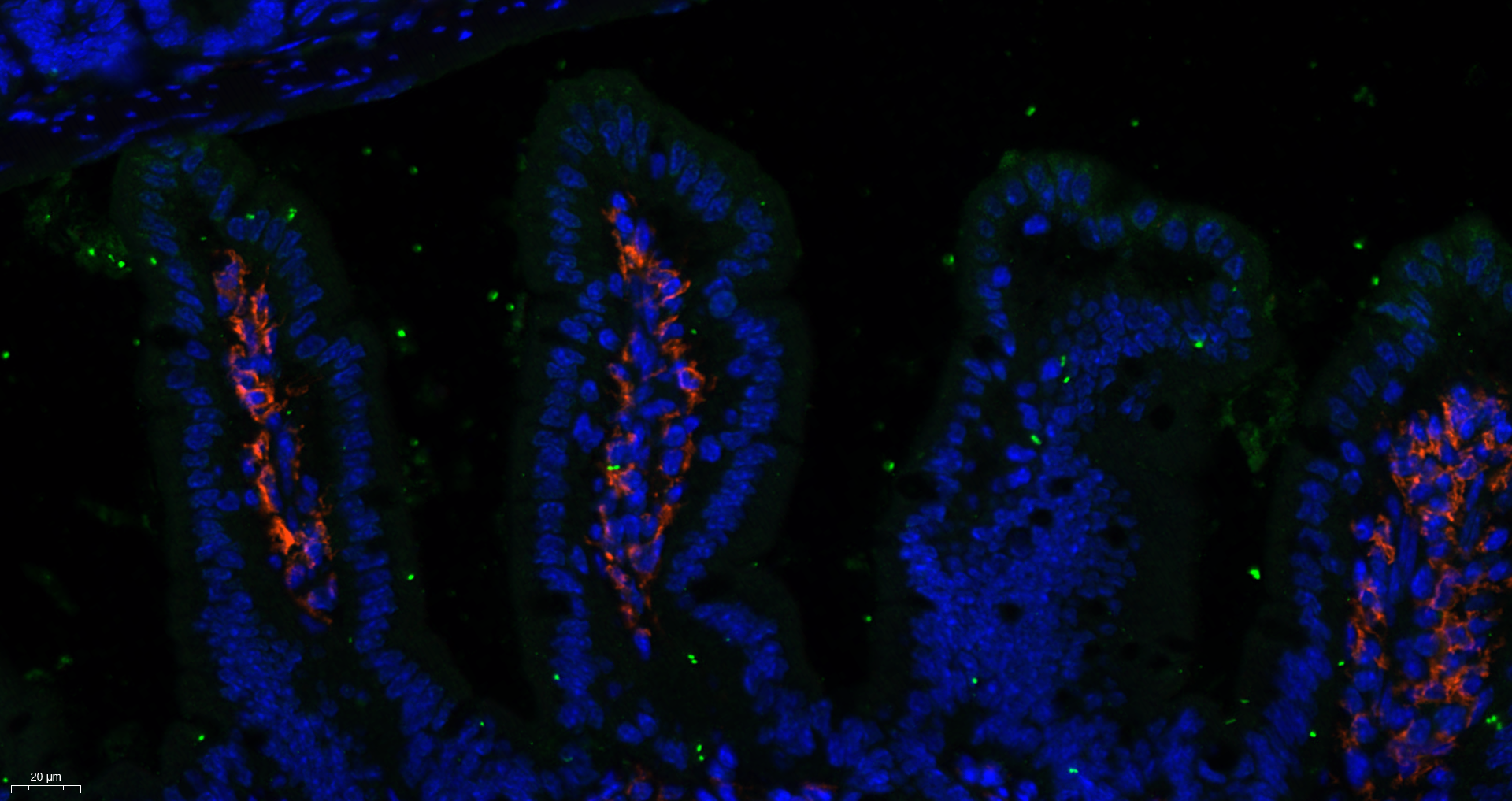

Supplement: Supplementary file 5 — Source data Fig. 1 [file 44318_2024_76_MOESM5_ESM.zip › Figure1/1D/1D-2.tif]

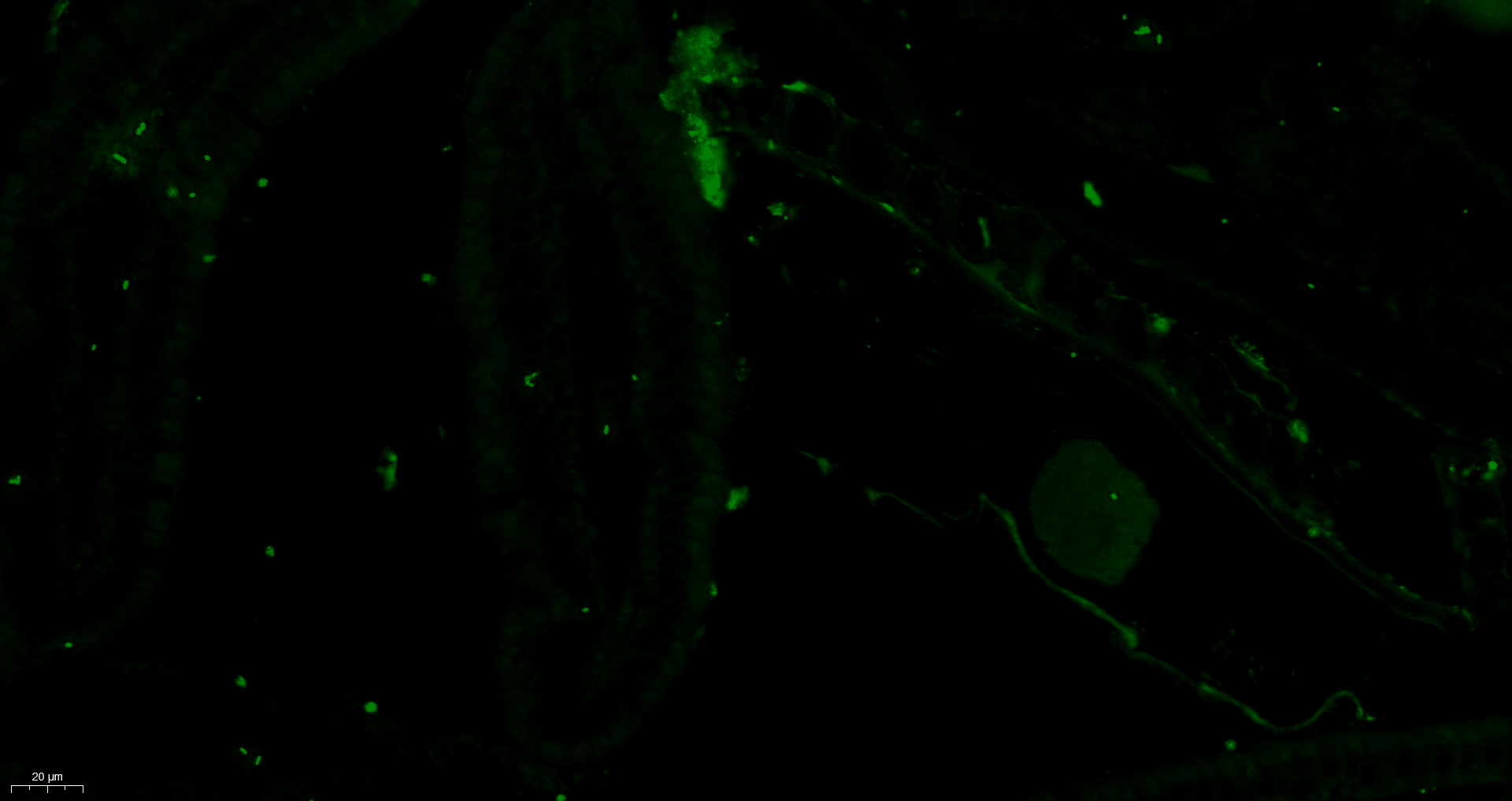

Supplement: Supplementary file 5 — Source data Fig. 1 [file 44318_2024_76_MOESM5_ESM.zip › Figure1/1D/1D-3 GREEN.jpg]

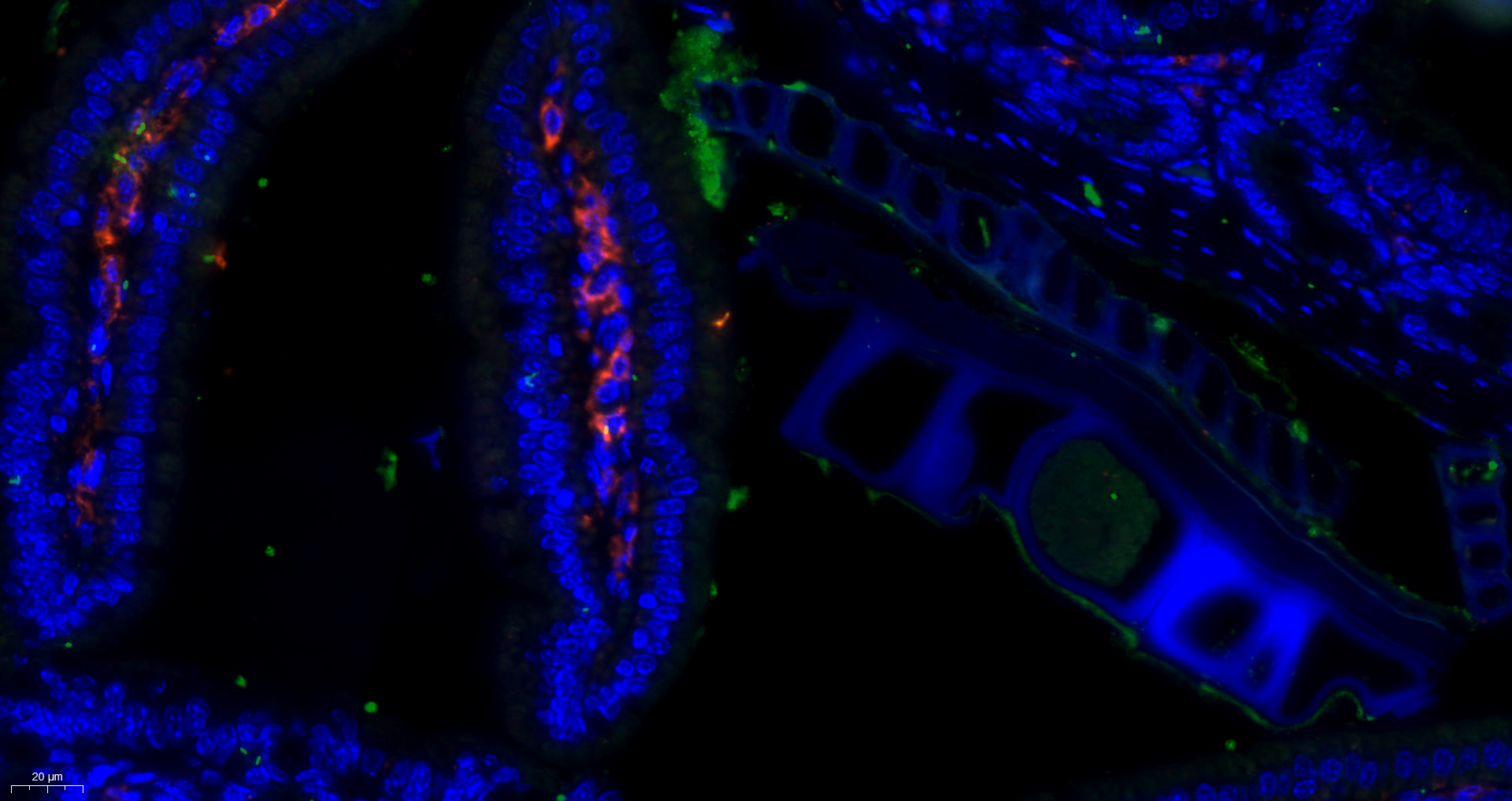

Supplement: Supplementary file 5 — Source data Fig. 1 [file 44318_2024_76_MOESM5_ESM.zip › Figure1/1D/1D-3.tif]

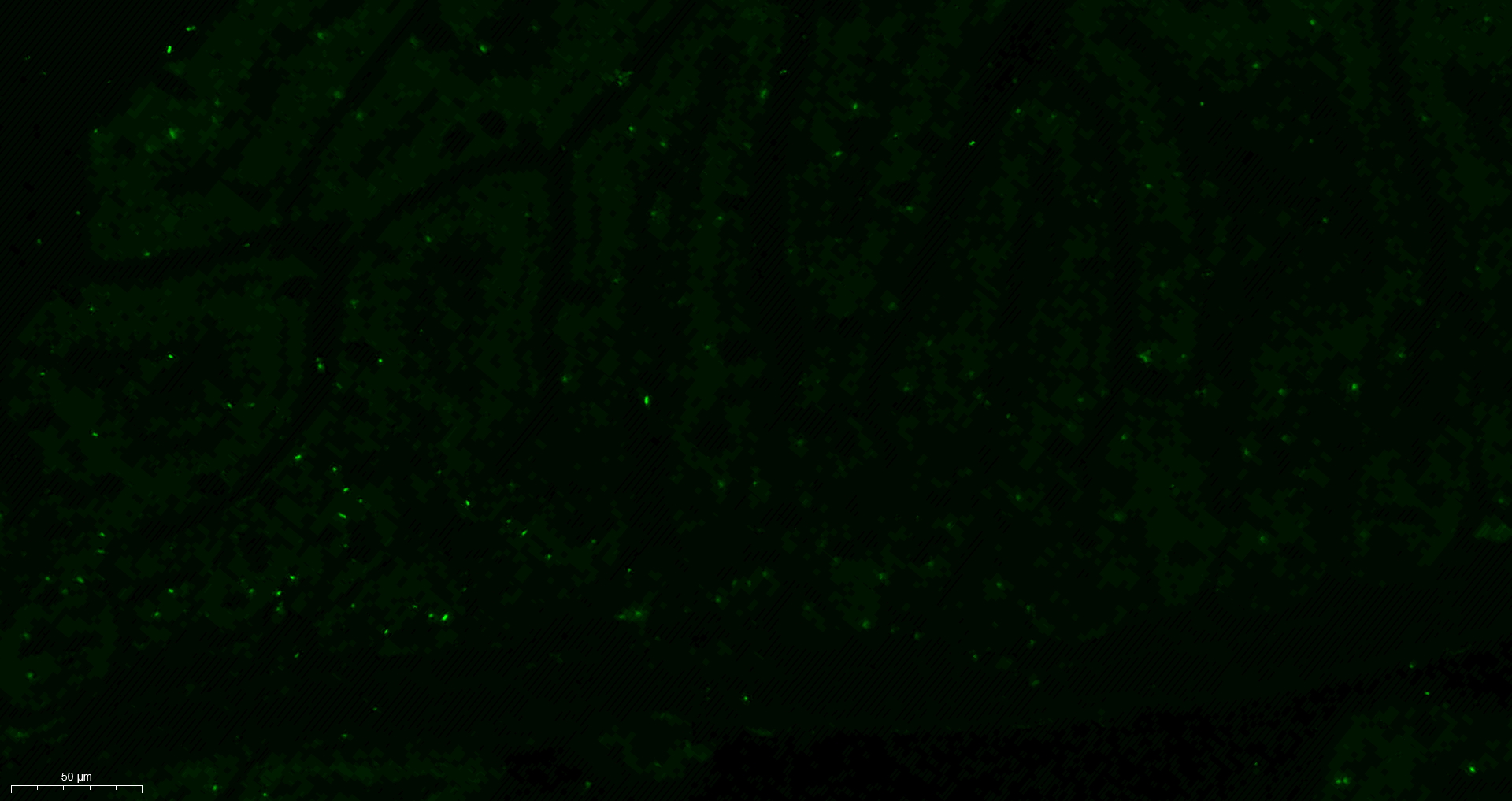

Supplement: Supplementary file 5 — Source data Fig. 1 [file 44318_2024_76_MOESM5_ESM.zip › Figure1/1D/1d-4 green.jpg]

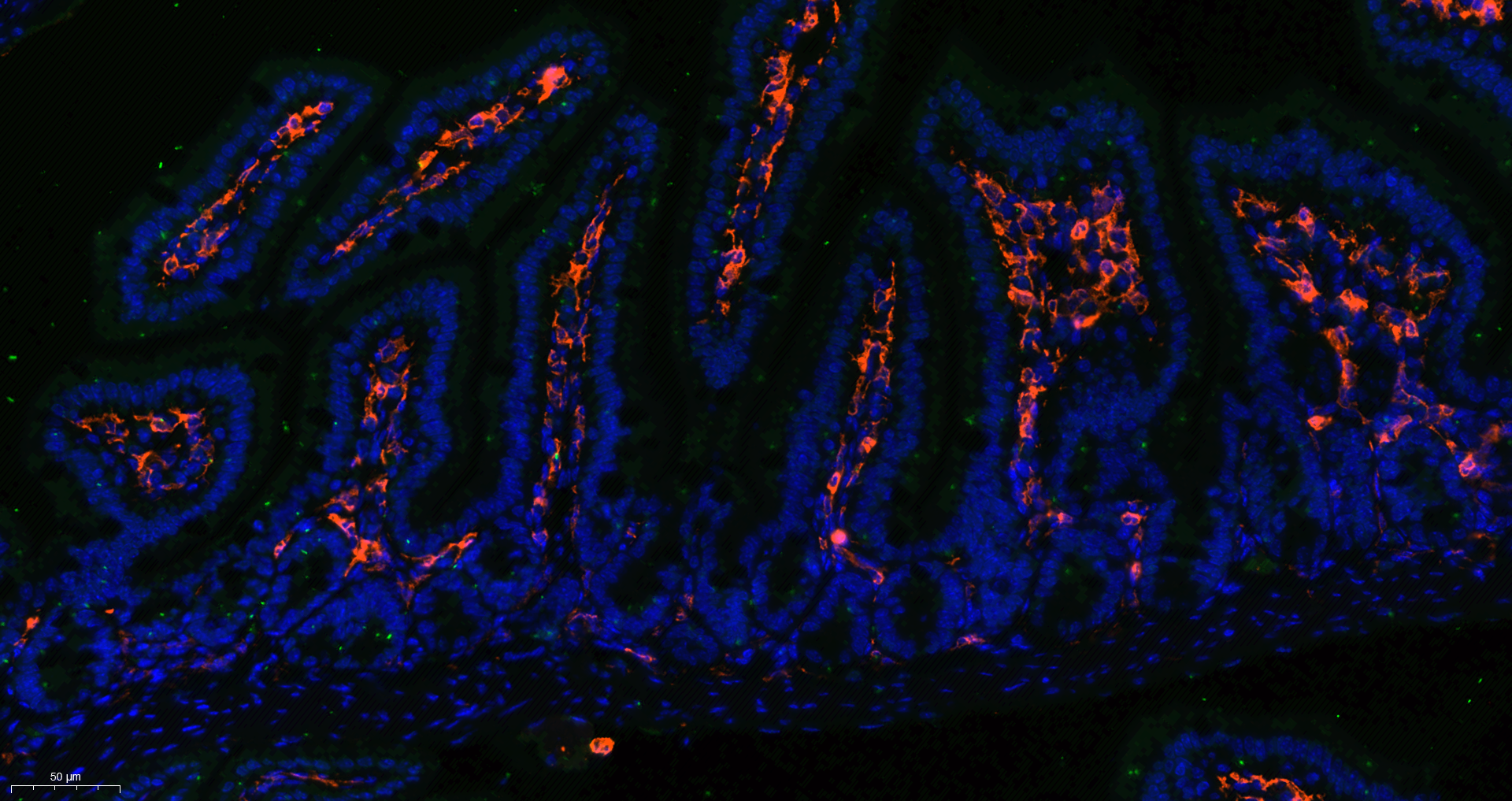

Supplement: Supplementary file 5 — Source data Fig. 1 [file 44318_2024_76_MOESM5_ESM.zip › Figure1/1D/1d-4.tif]

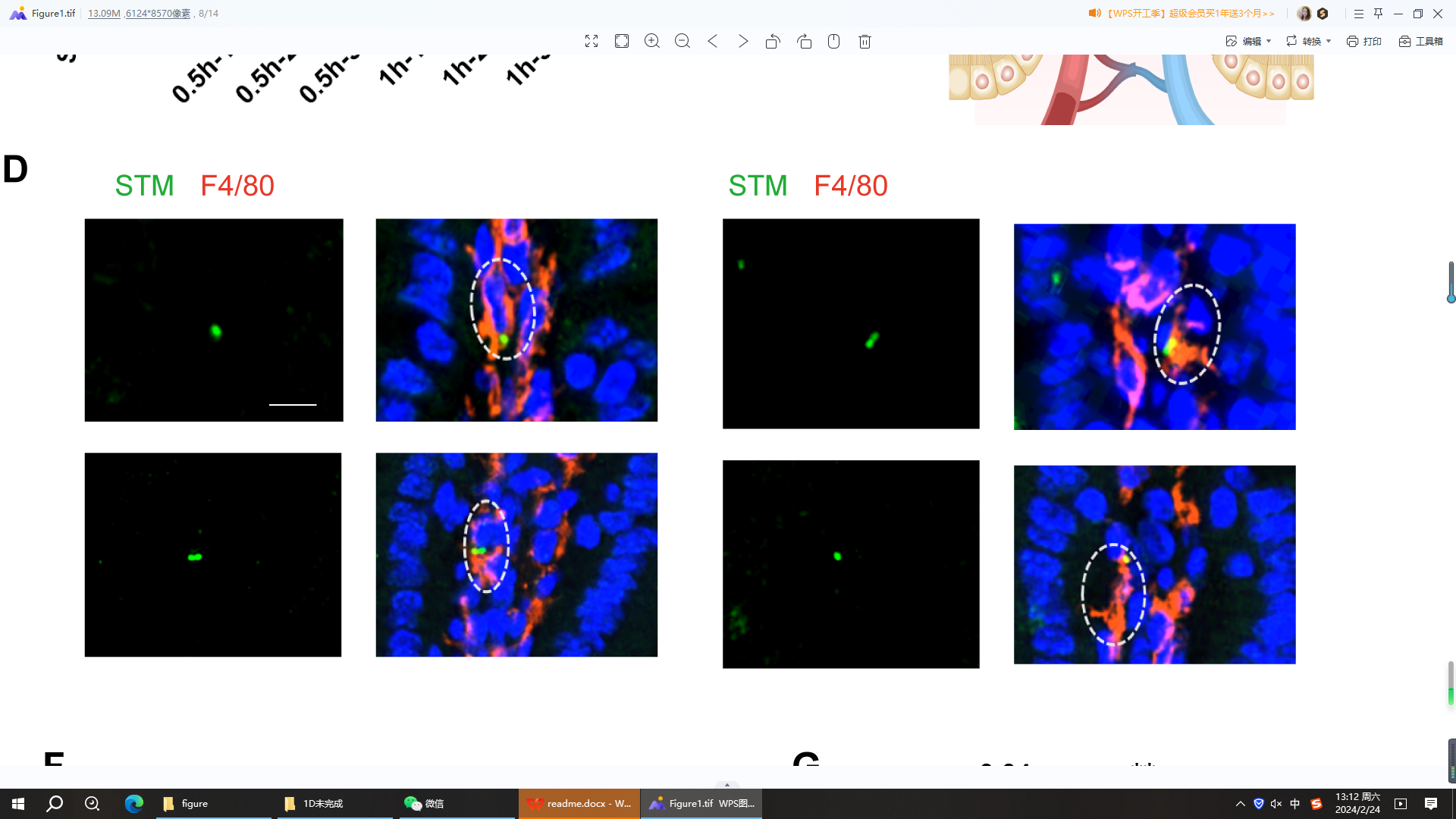


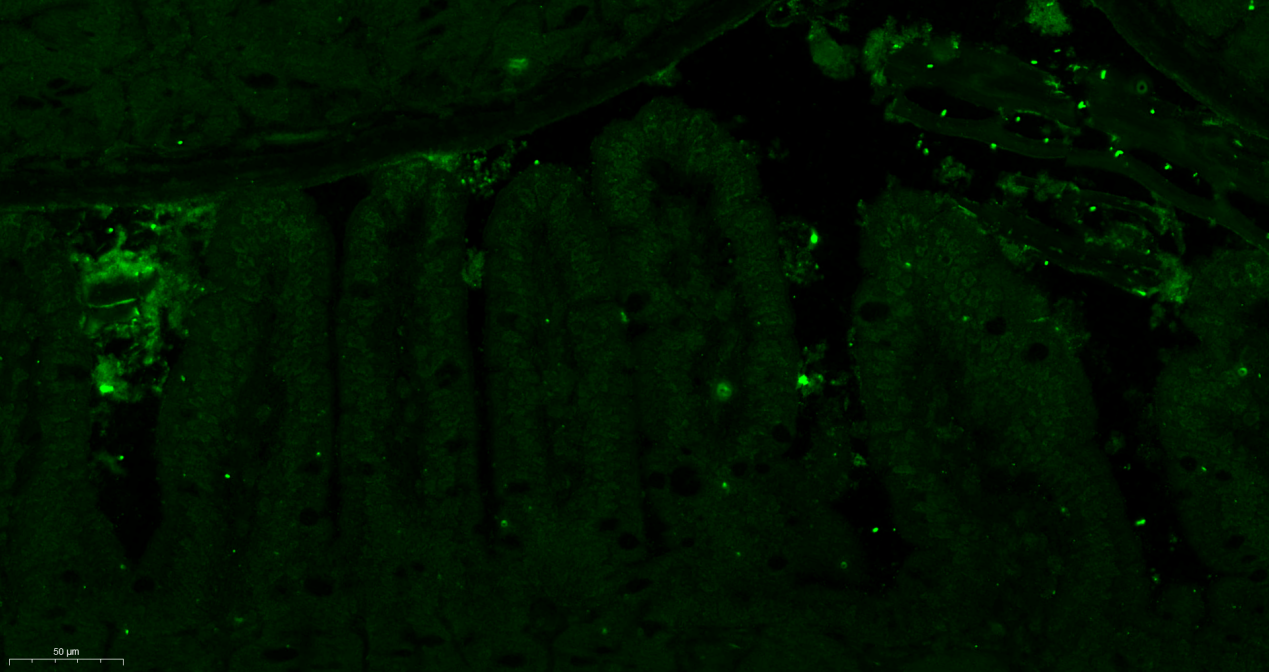


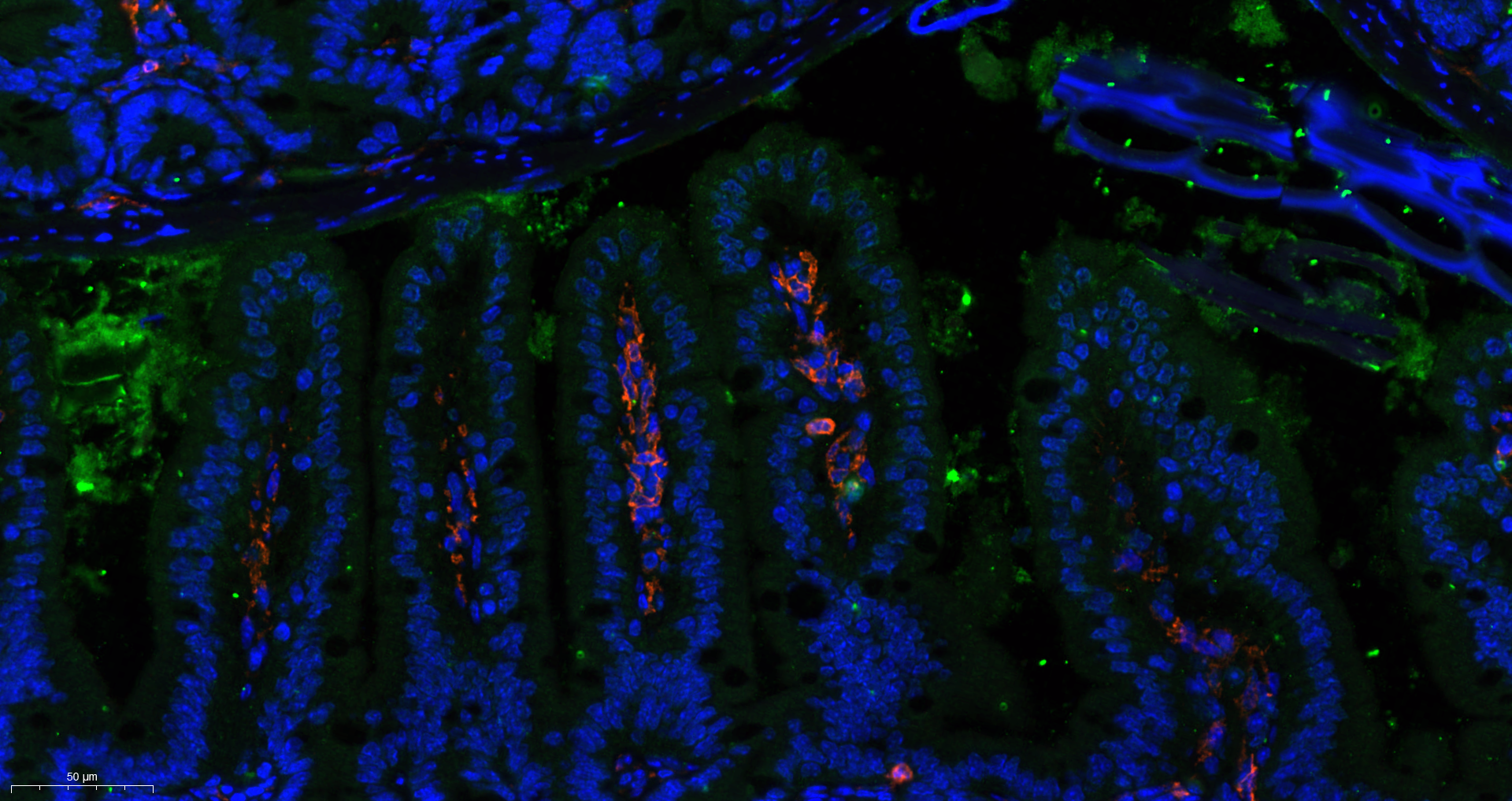


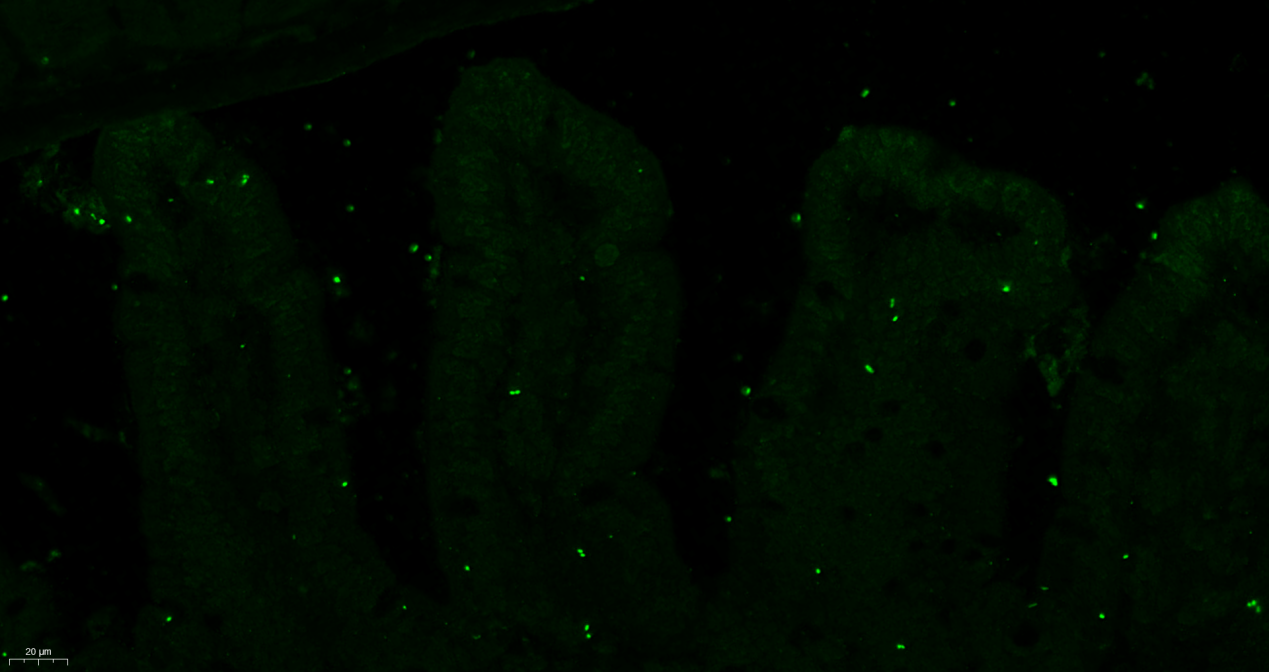


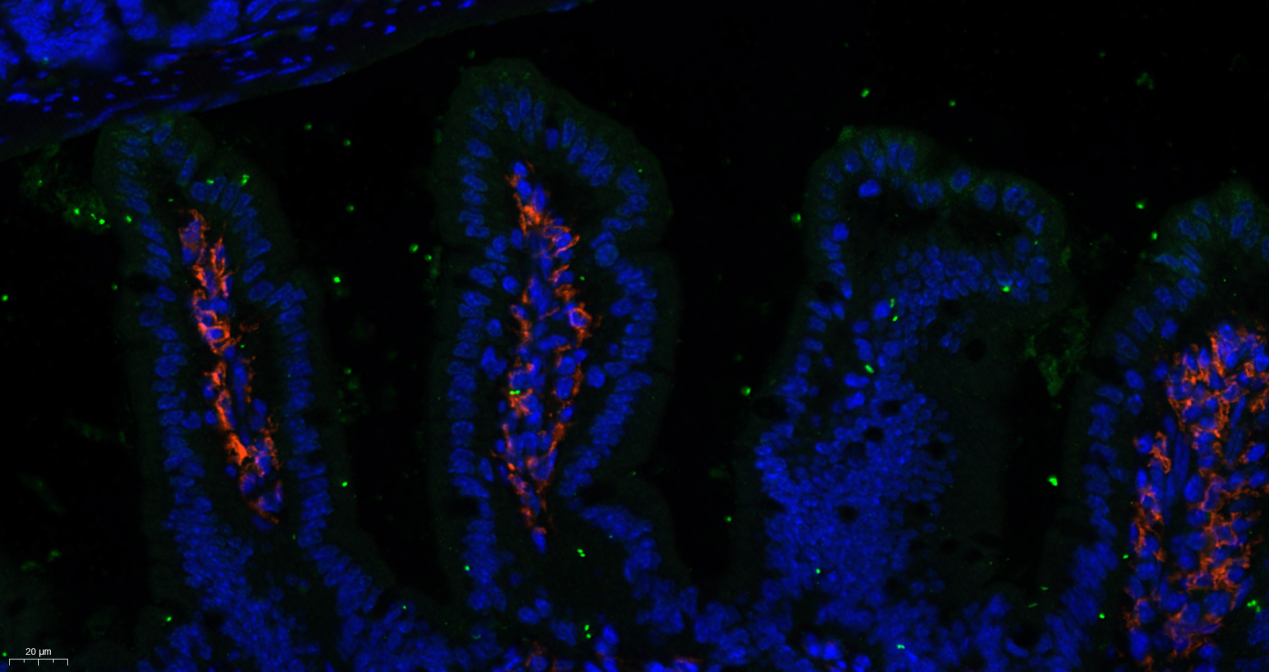


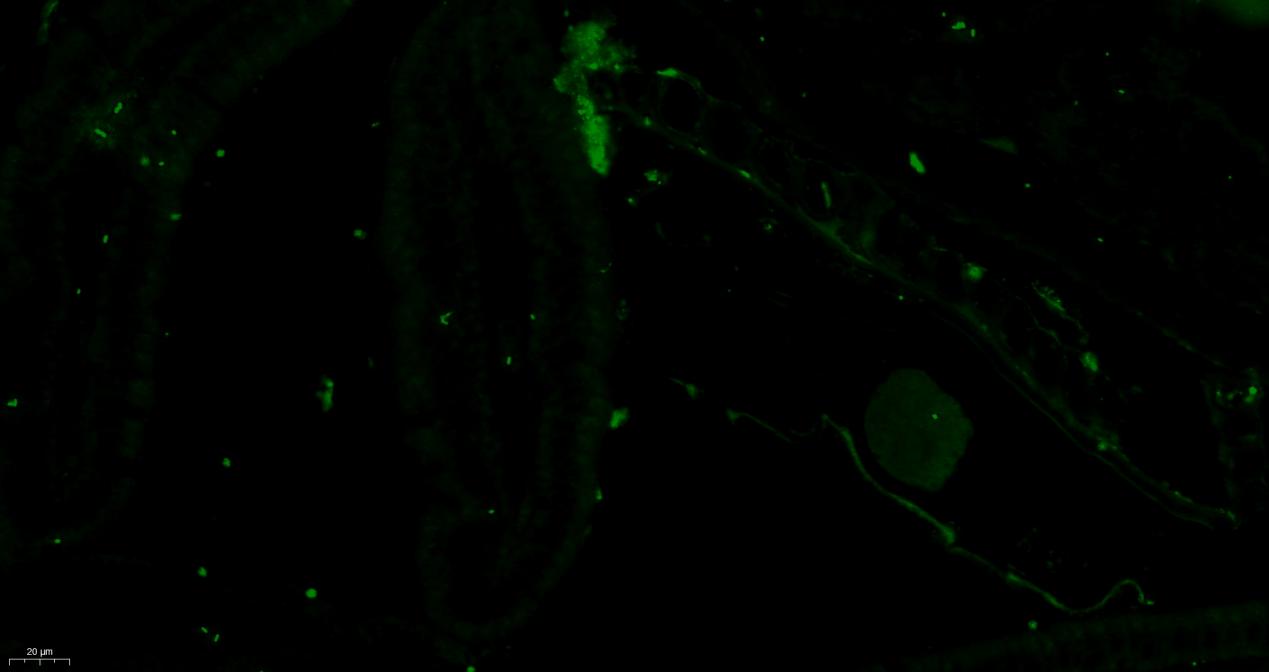


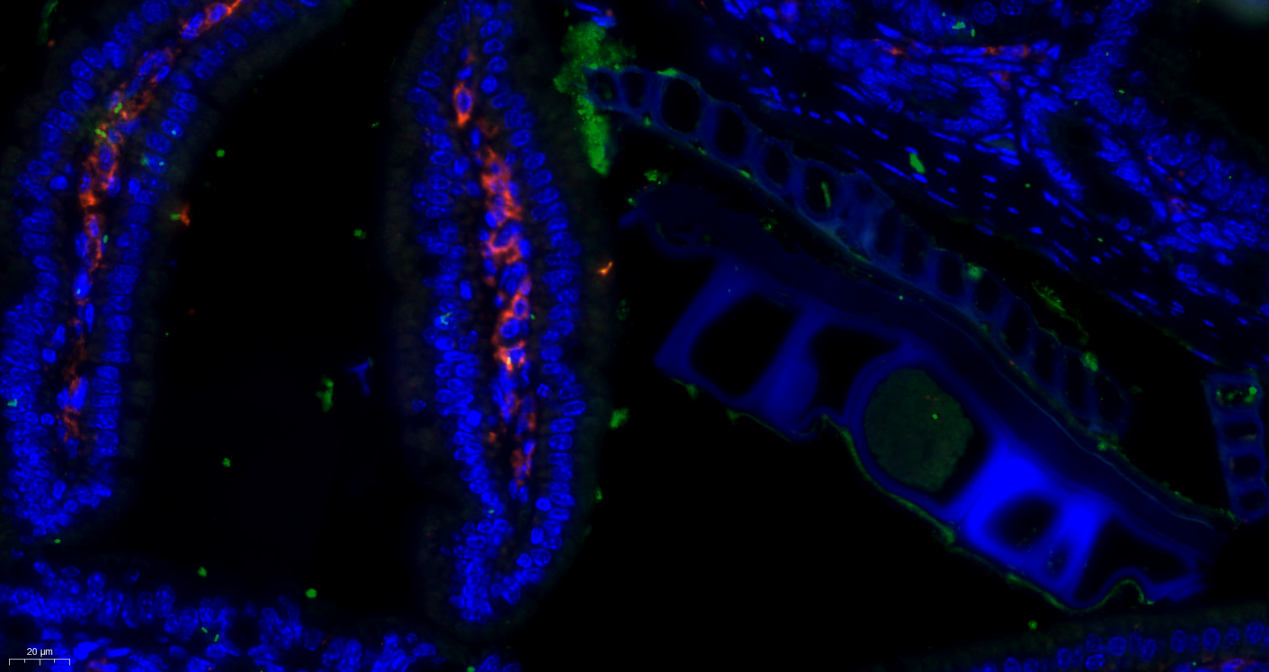


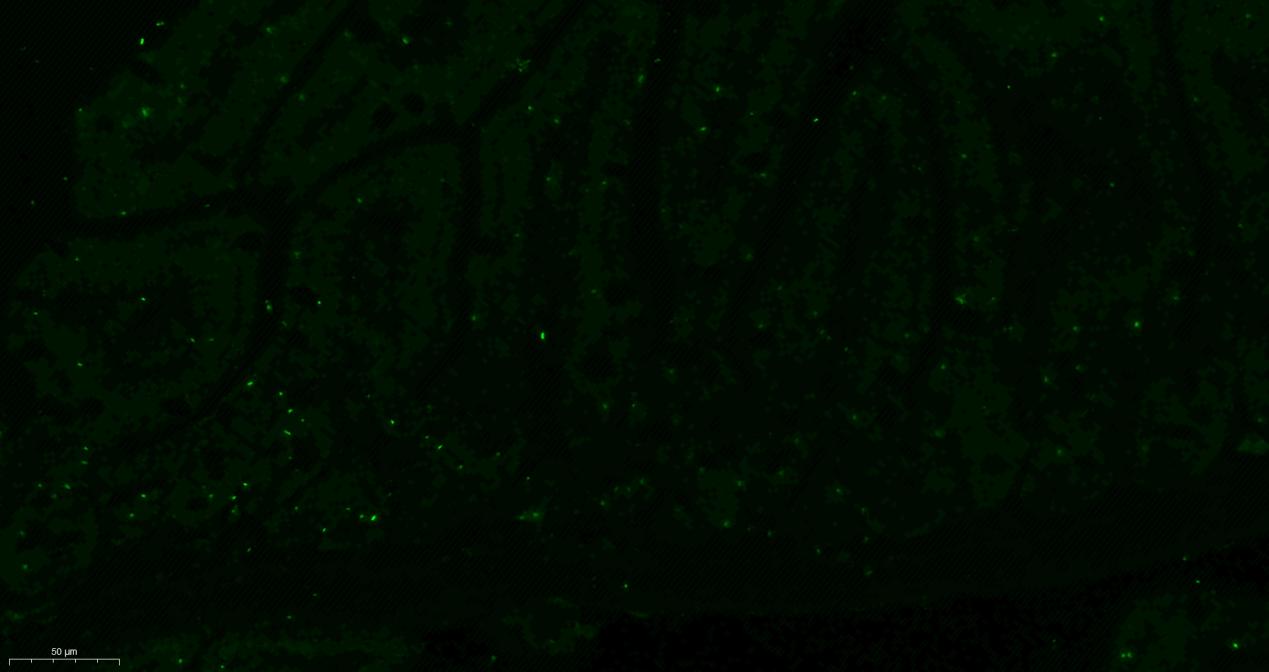


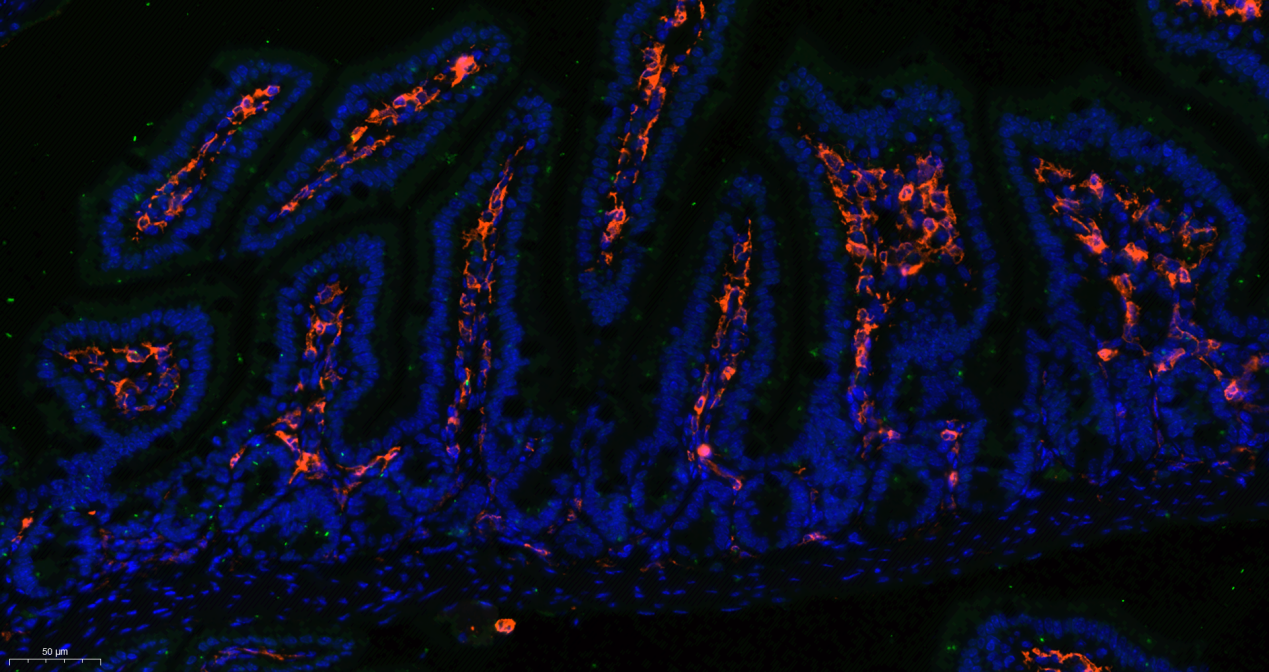

Supplement: Supplementary file 5 — Source data Fig. 1 [file 44318_2024_76_MOESM5_ESM.zip › Figure1/1D/readme.docx]

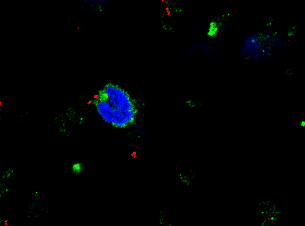

Supplement: Supplementary file 5 — Source data Fig. 1 [file 44318_2024_76_MOESM5_ESM.zip › Figure1/1L/Fig1L-2.tif]

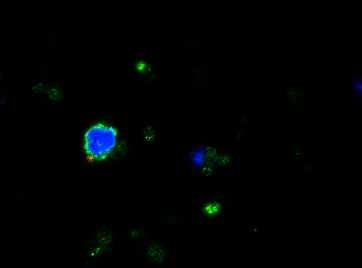

Supplement: Supplementary file 5 — Source data Fig. 1 [file 44318_2024_76_MOESM5_ESM.zip › Figure1/1L/Fih1L-1.tif]

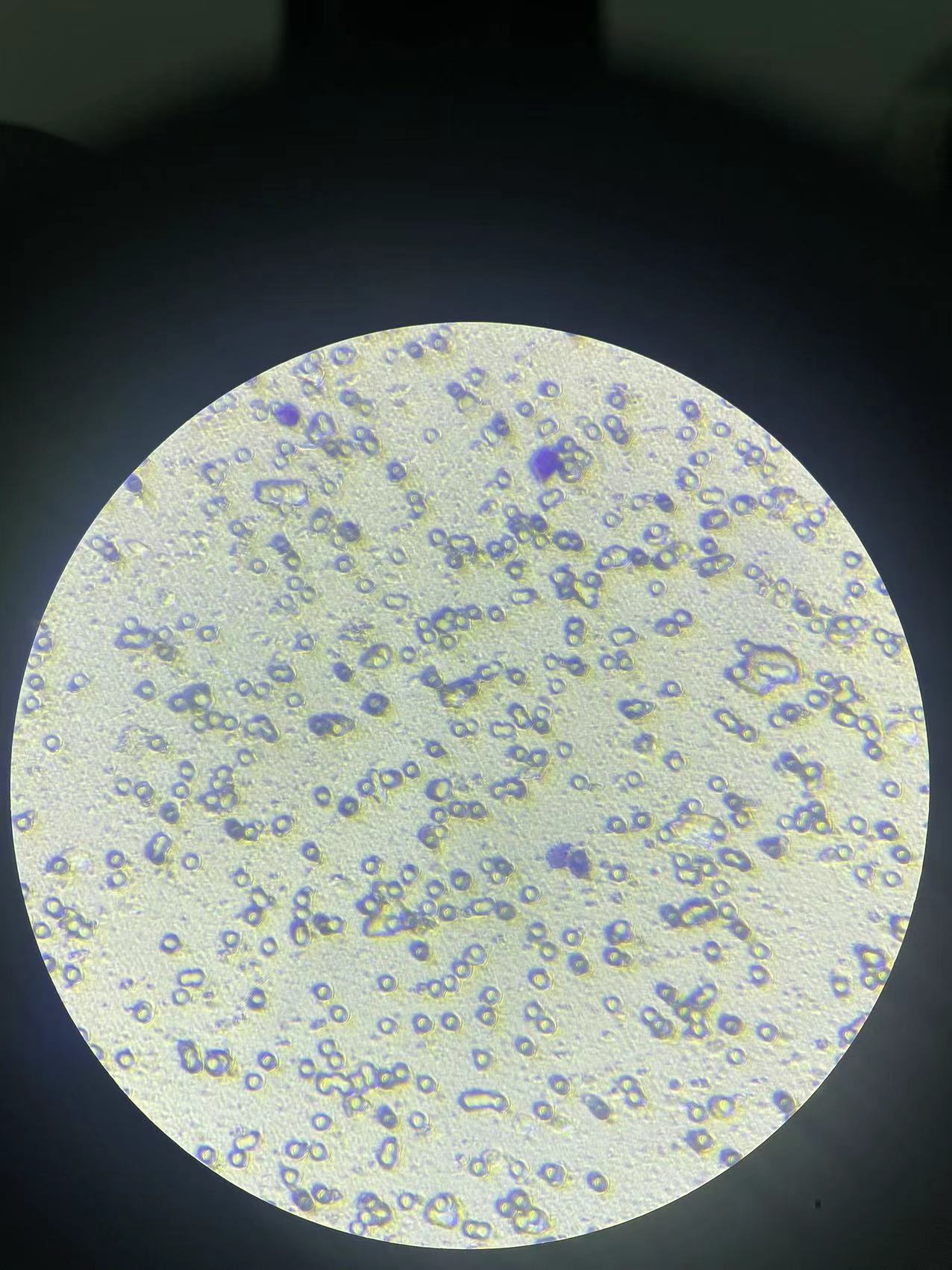

Supplement: Supplementary file 6 — Source data Fig. 2 [file 44318_2024_76_MOESM6_ESM.zip › Figure2/2A/Invasion/Ctrl.jpg]

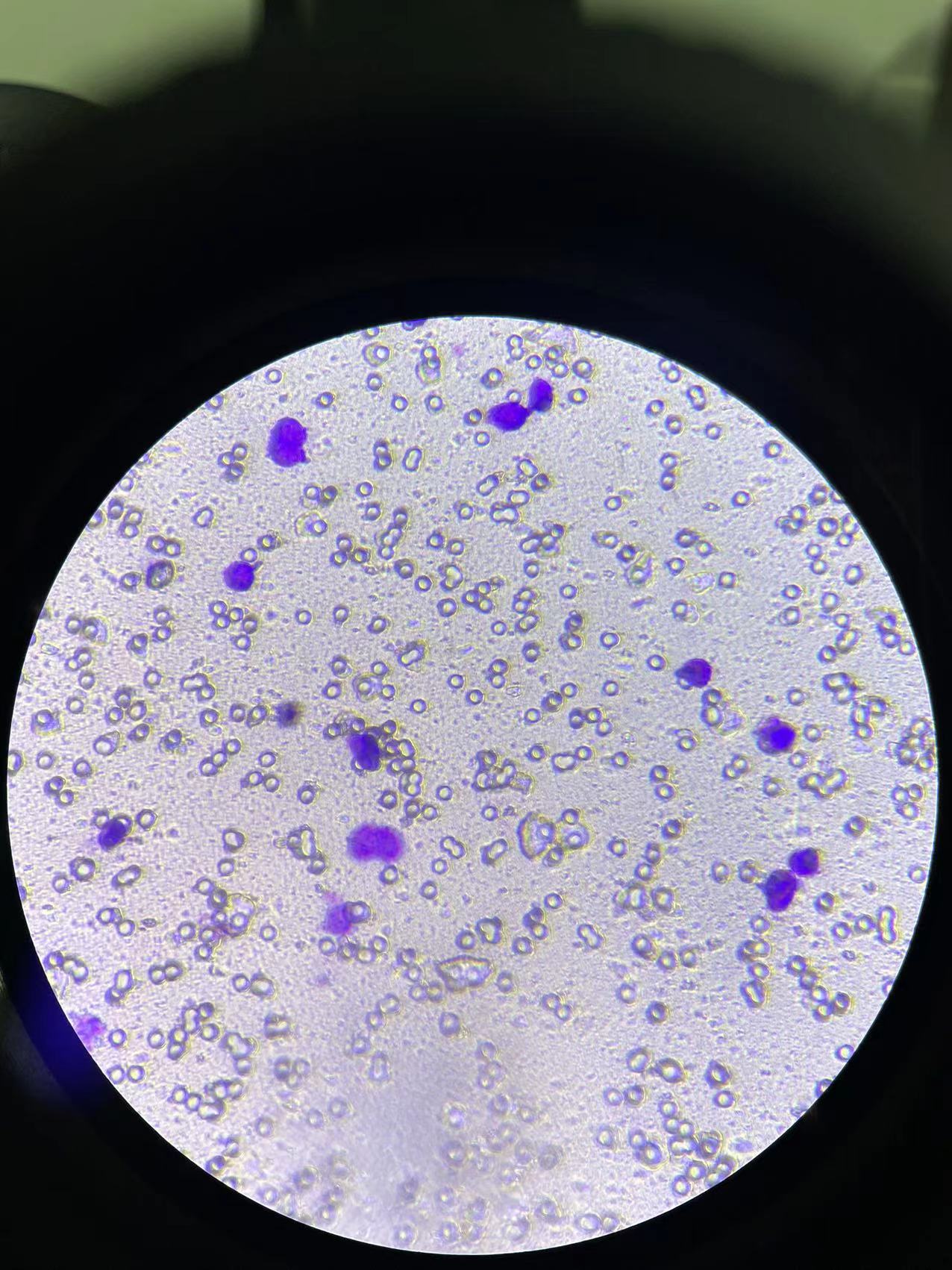

Supplement: Supplementary file 6 — Source data Fig. 2 [file 44318_2024_76_MOESM6_ESM.zip › Figure2/2A/Invasion/moi1.jpg]

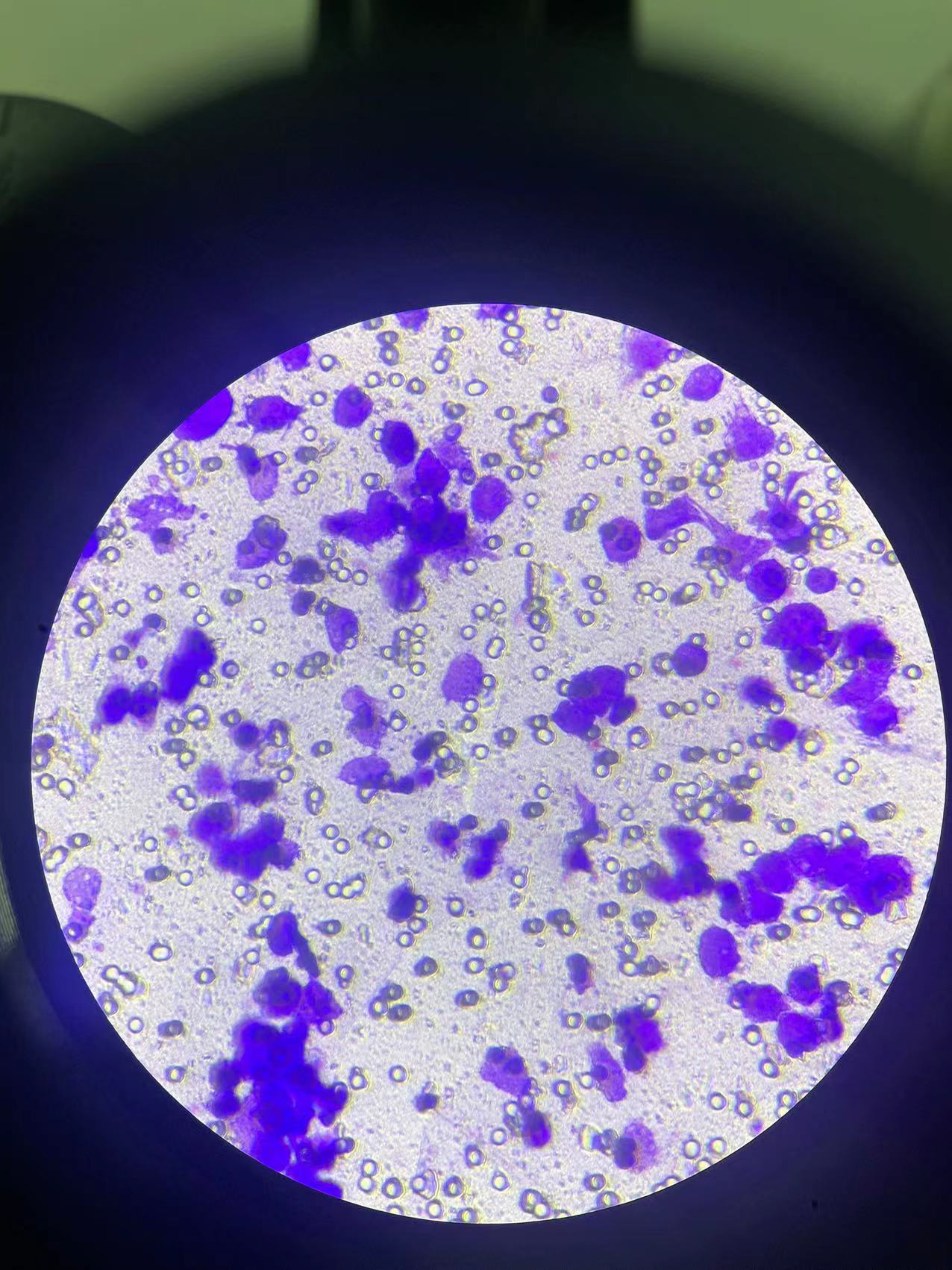

Supplement: Supplementary file 6 — Source data Fig. 2 [file 44318_2024_76_MOESM6_ESM.zip › Figure2/2A/Invasion/moi10.jpg]

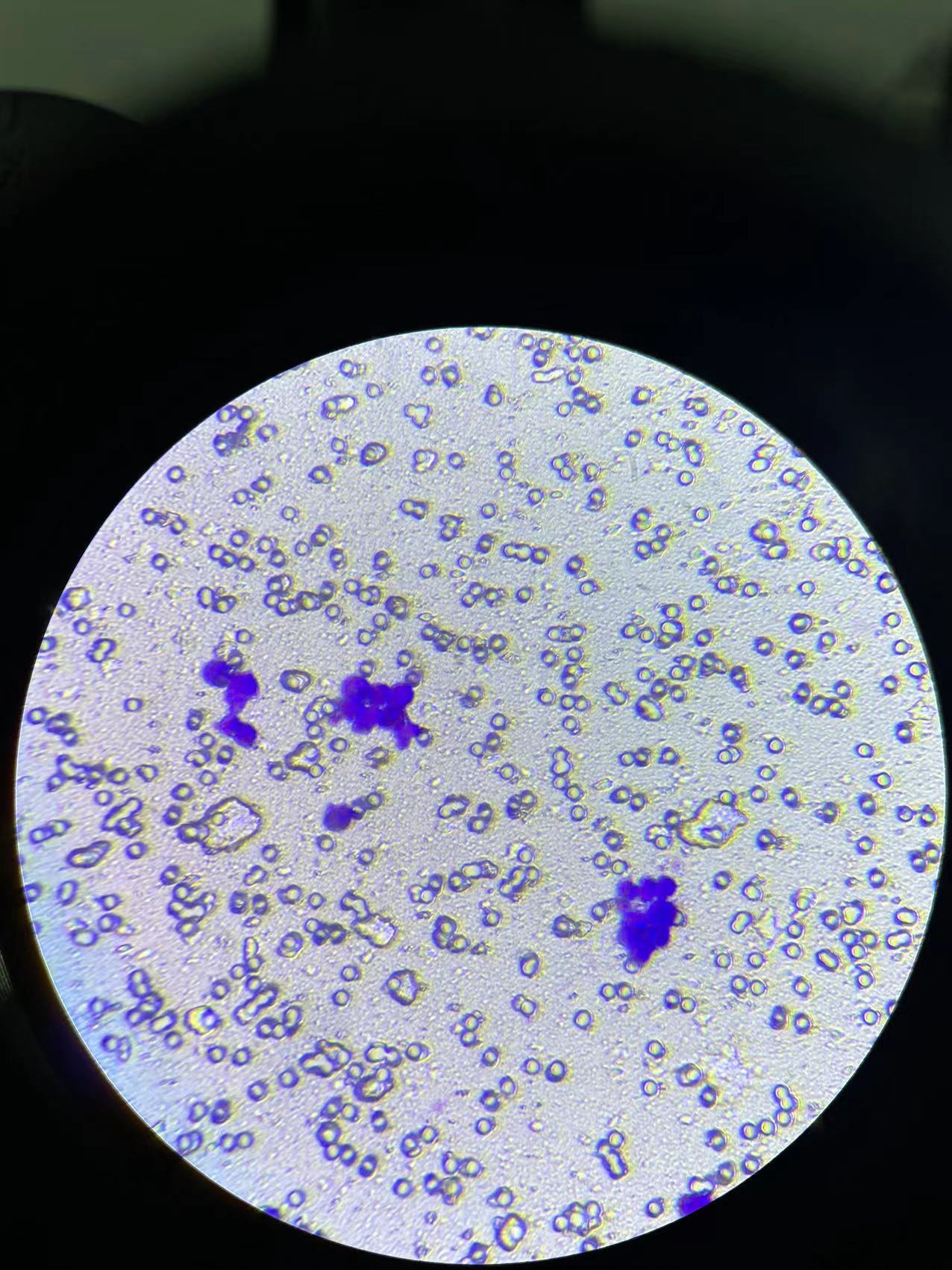

Supplement: Supplementary file 6 — Source data Fig. 2 [file 44318_2024_76_MOESM6_ESM.zip › Figure2/2A/Invasion/moi5.jpg]

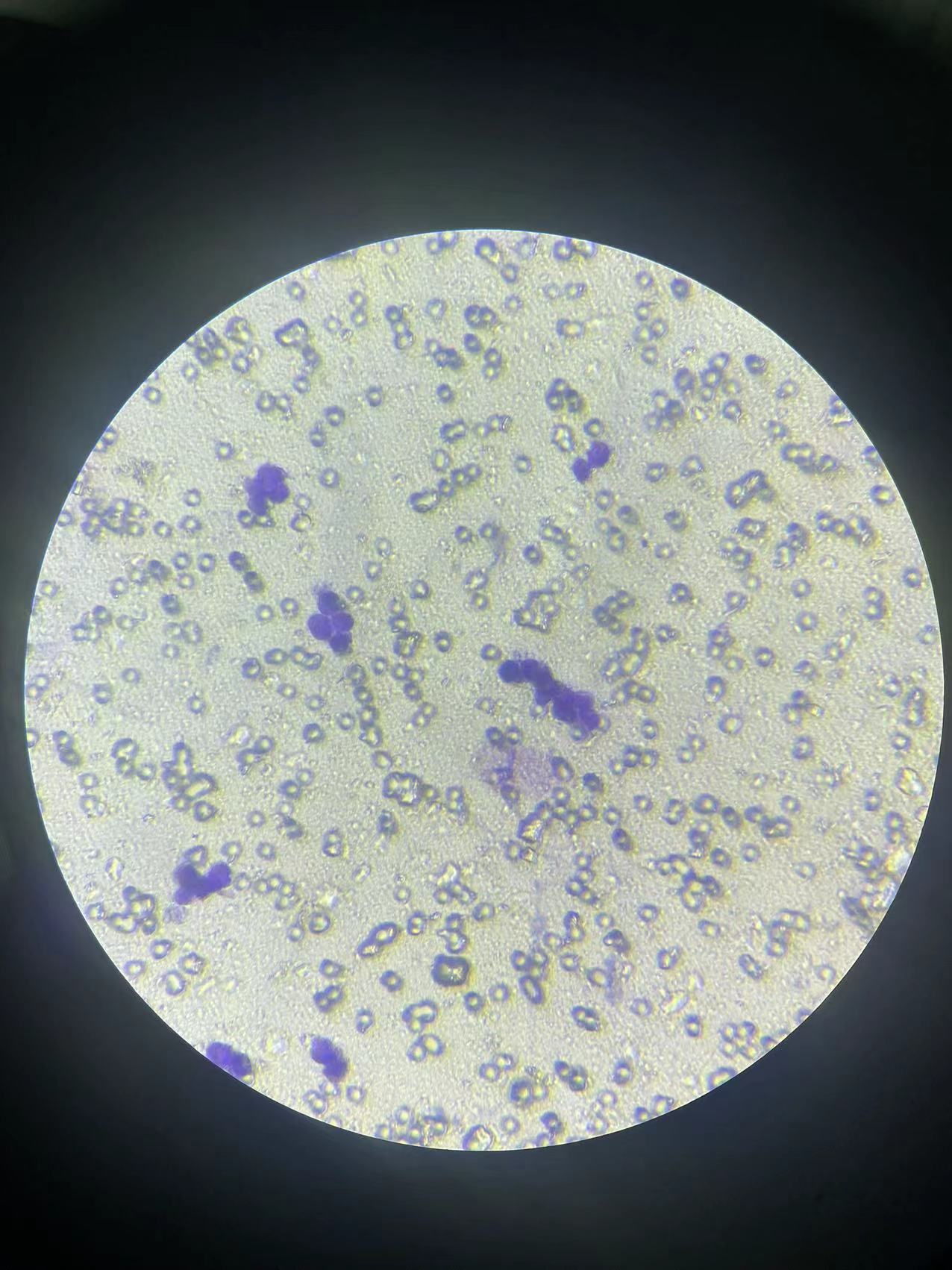

Supplement: Supplementary file 6 — Source data Fig. 2 [file 44318_2024_76_MOESM6_ESM.zip › Figure2/2A/Migration/CTRL.jpg]

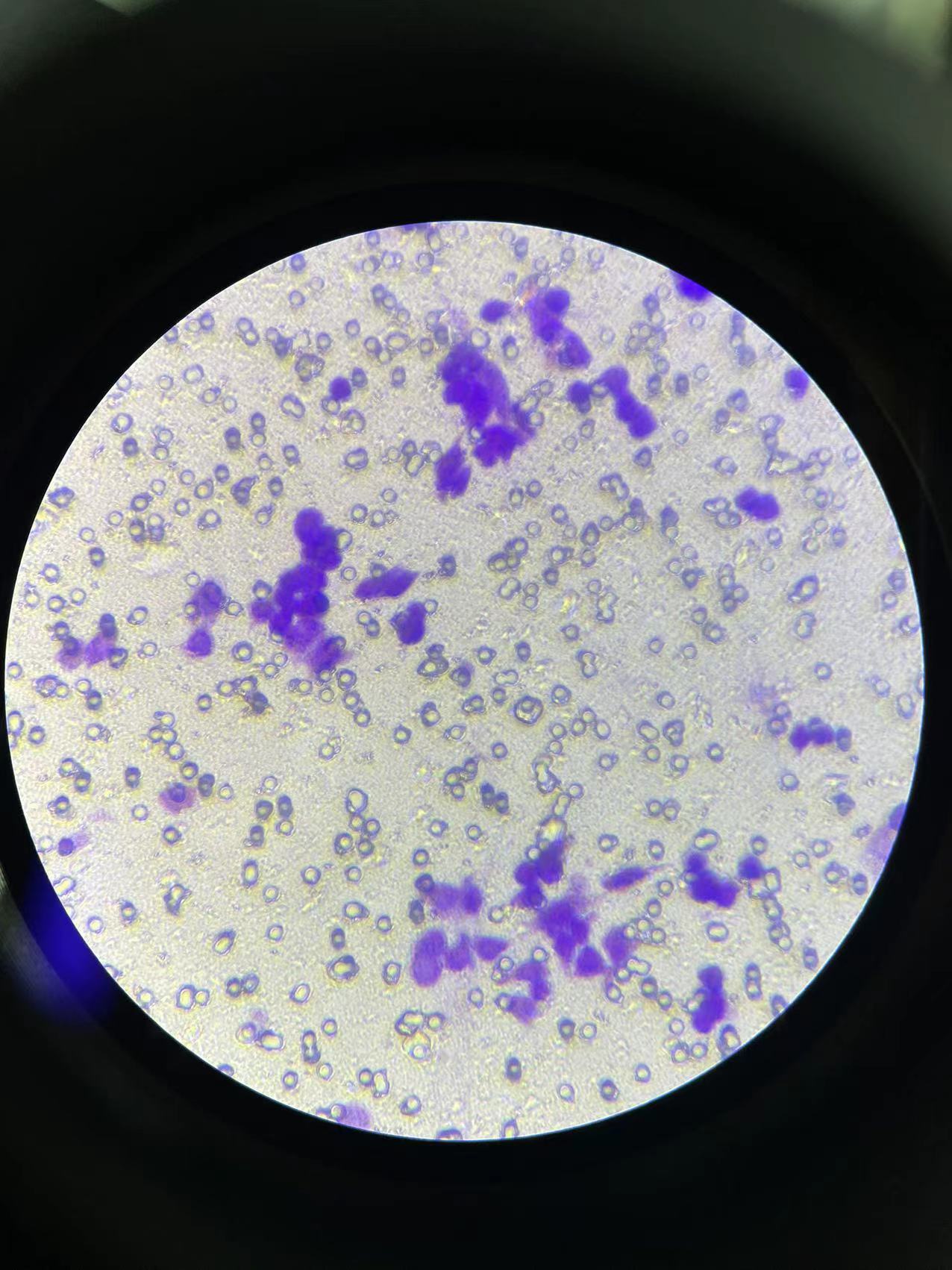

Supplement: Supplementary file 6 — Source data Fig. 2 [file 44318_2024_76_MOESM6_ESM.zip › Figure2/2A/Migration/MOI1.jpg]

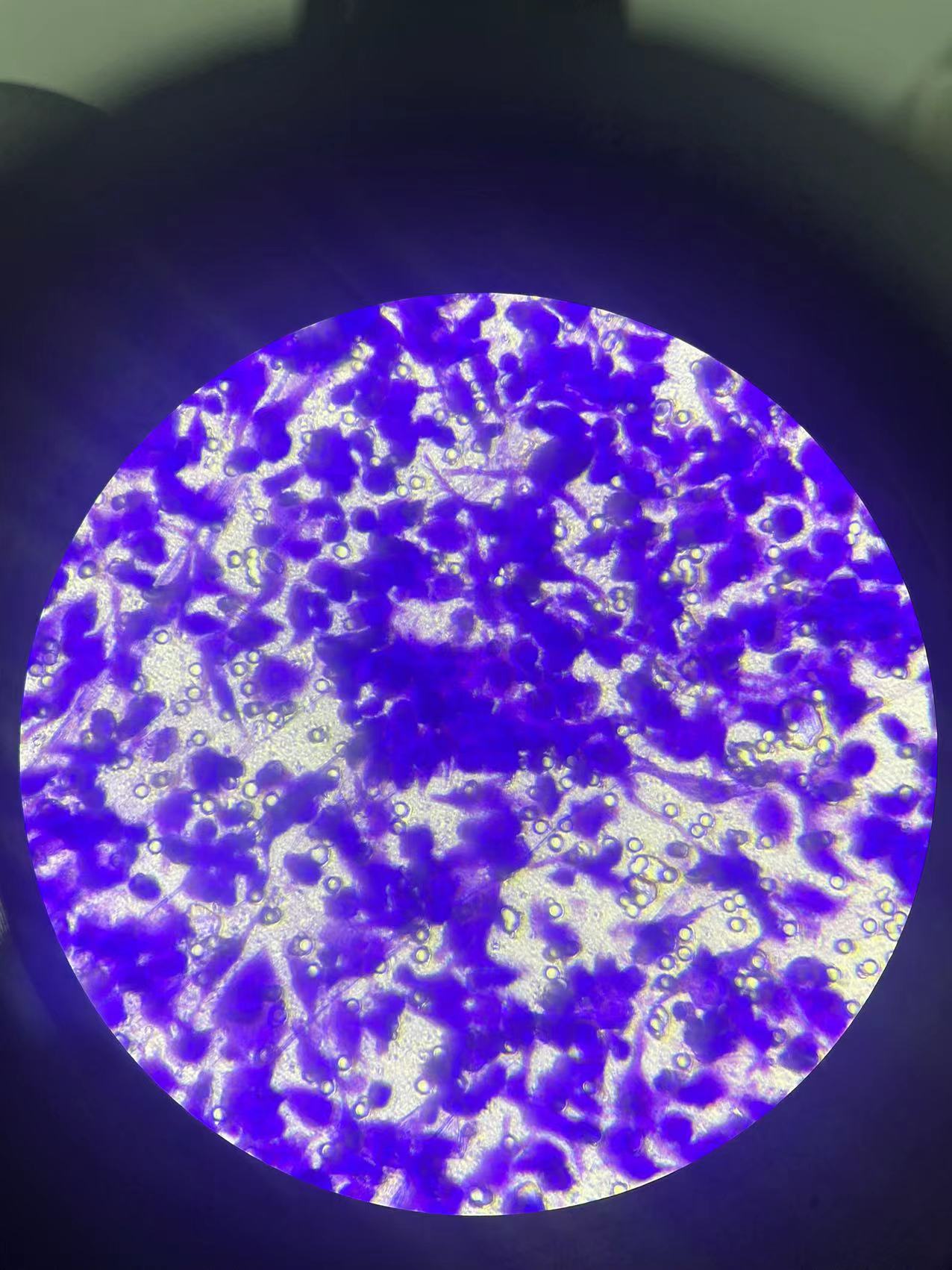

Supplement: Supplementary file 6 — Source data Fig. 2 [file 44318_2024_76_MOESM6_ESM.zip › Figure2/2A/Migration/MOI10.jpg]

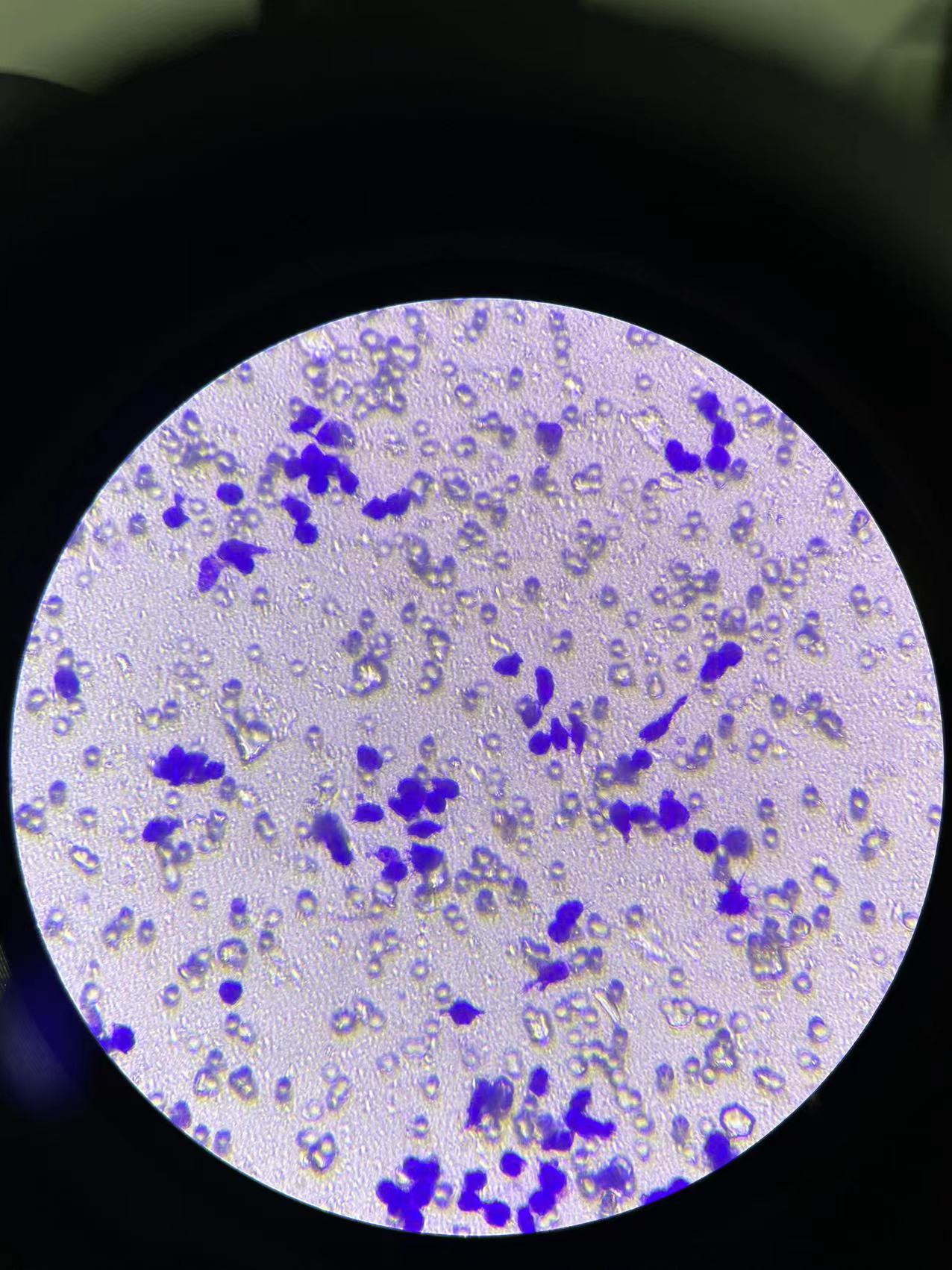

Supplement: Supplementary file 6 — Source data Fig. 2 [file 44318_2024_76_MOESM6_ESM.zip › Figure2/2A/Migration/MOI5.jpg]

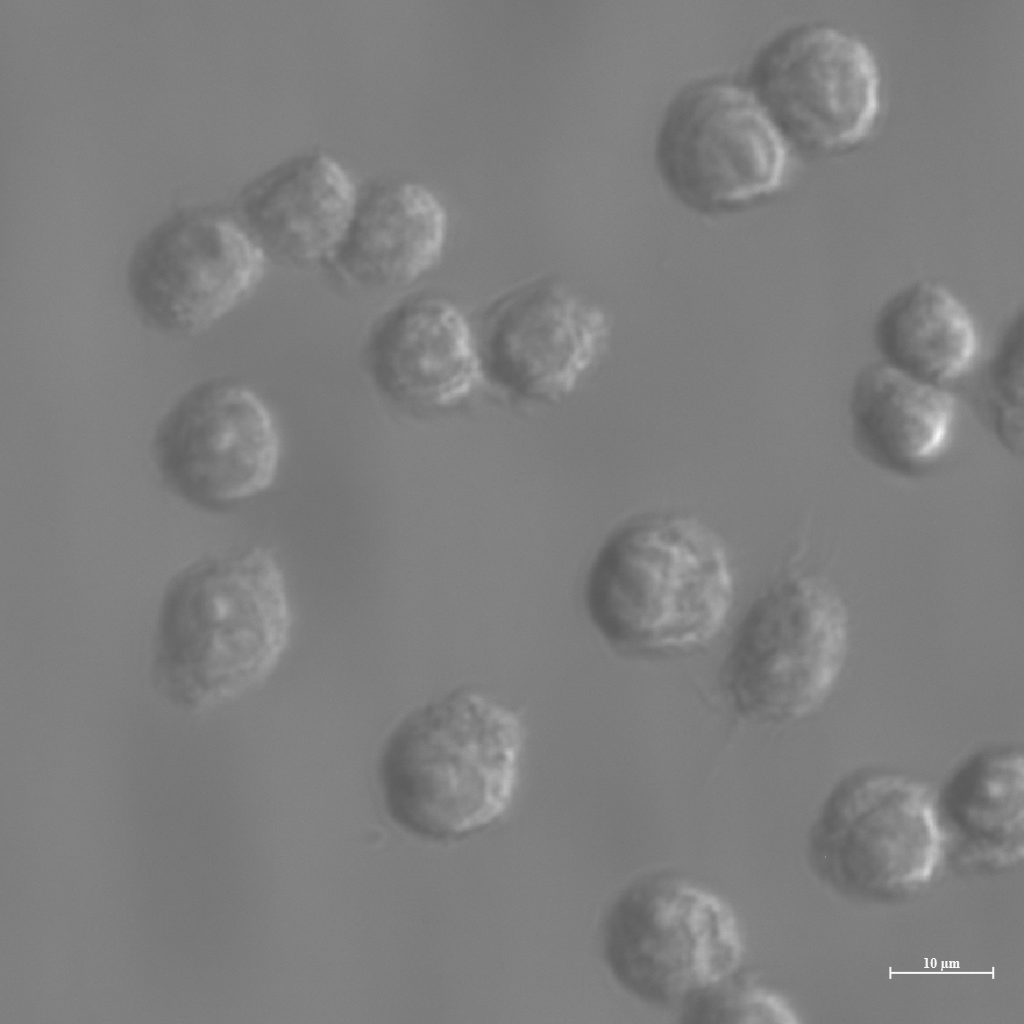

Supplement: Supplementary file 6 — Source data Fig. 2 [file 44318_2024_76_MOESM6_ESM.zip › Figure2/2G/RAW-1.tif]

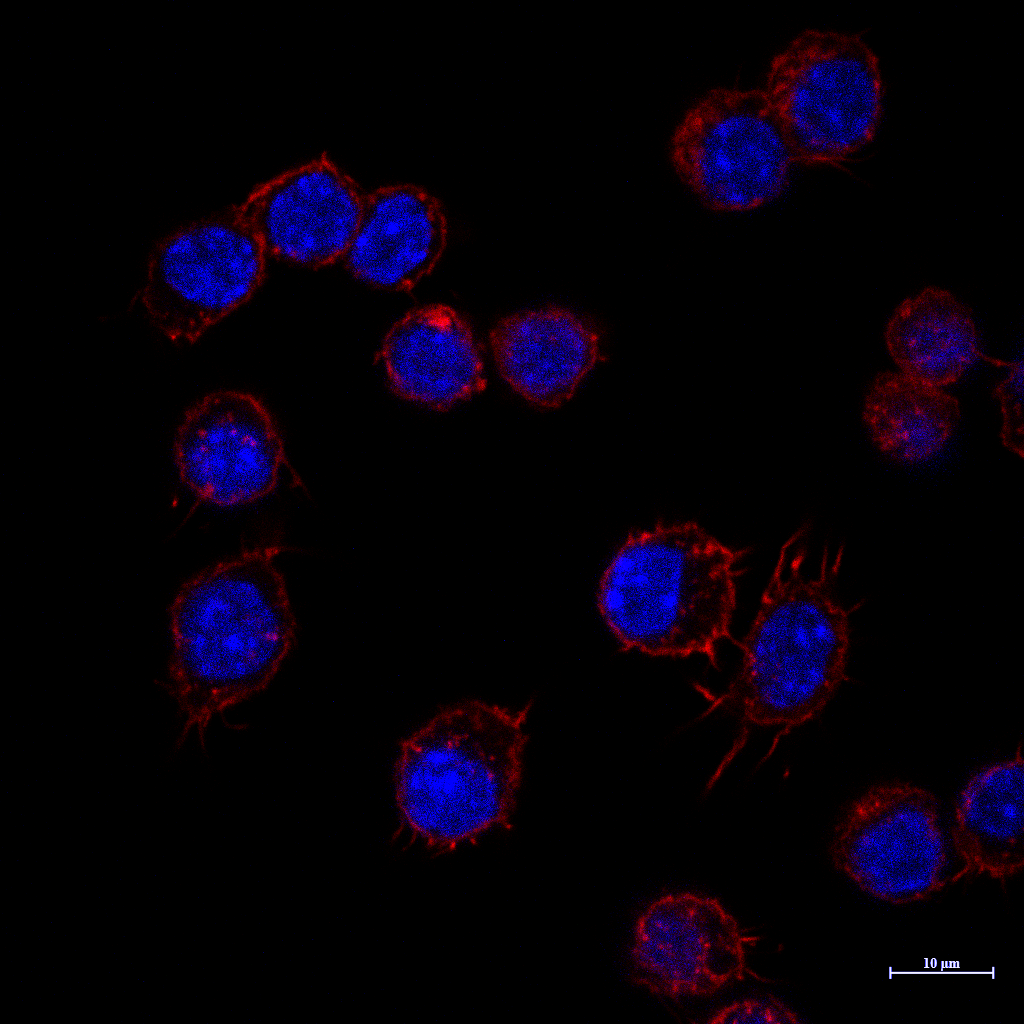

Supplement: Supplementary file 6 — Source data Fig. 2 [file 44318_2024_76_MOESM6_ESM.zip › Figure2/2G/RAW.tif]

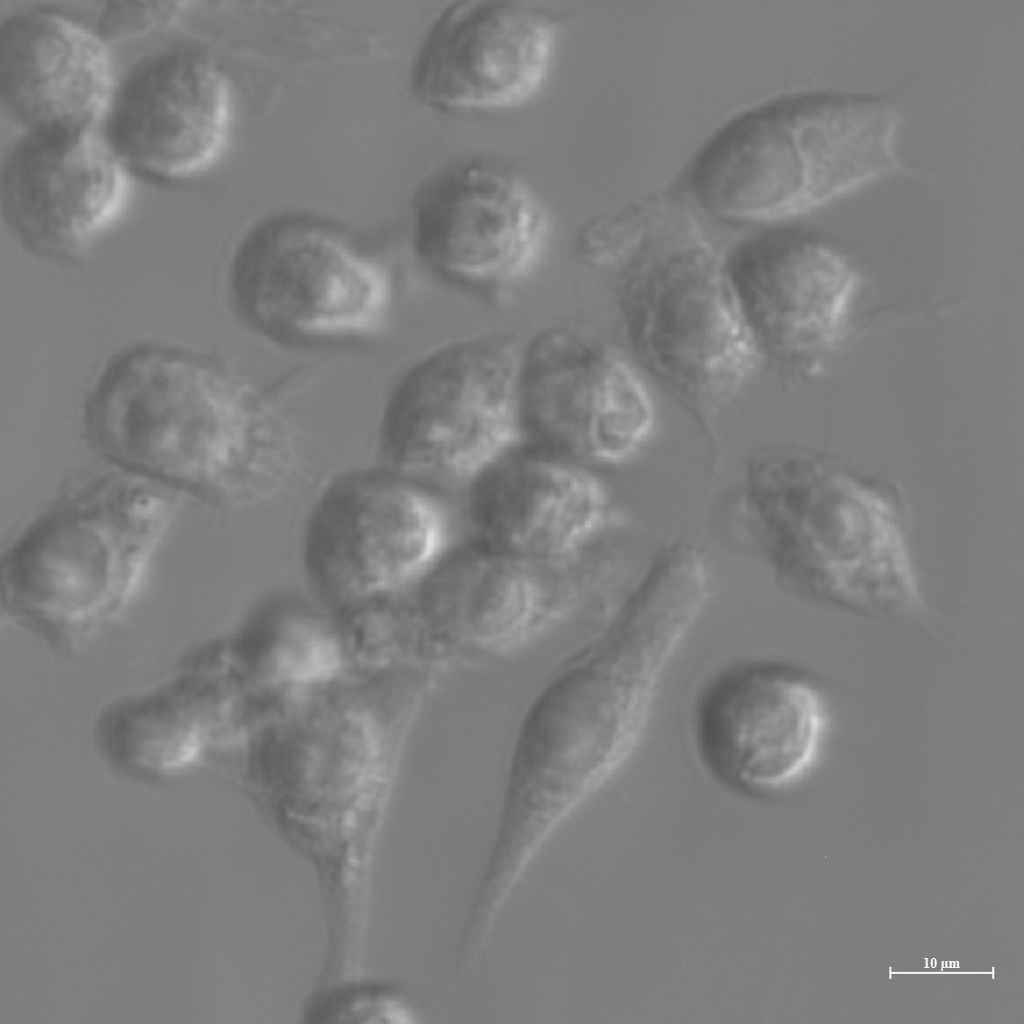

Supplement: Supplementary file 6 — Source data Fig. 2 [file 44318_2024_76_MOESM6_ESM.zip › Figure2/2G/WT-1.tif]

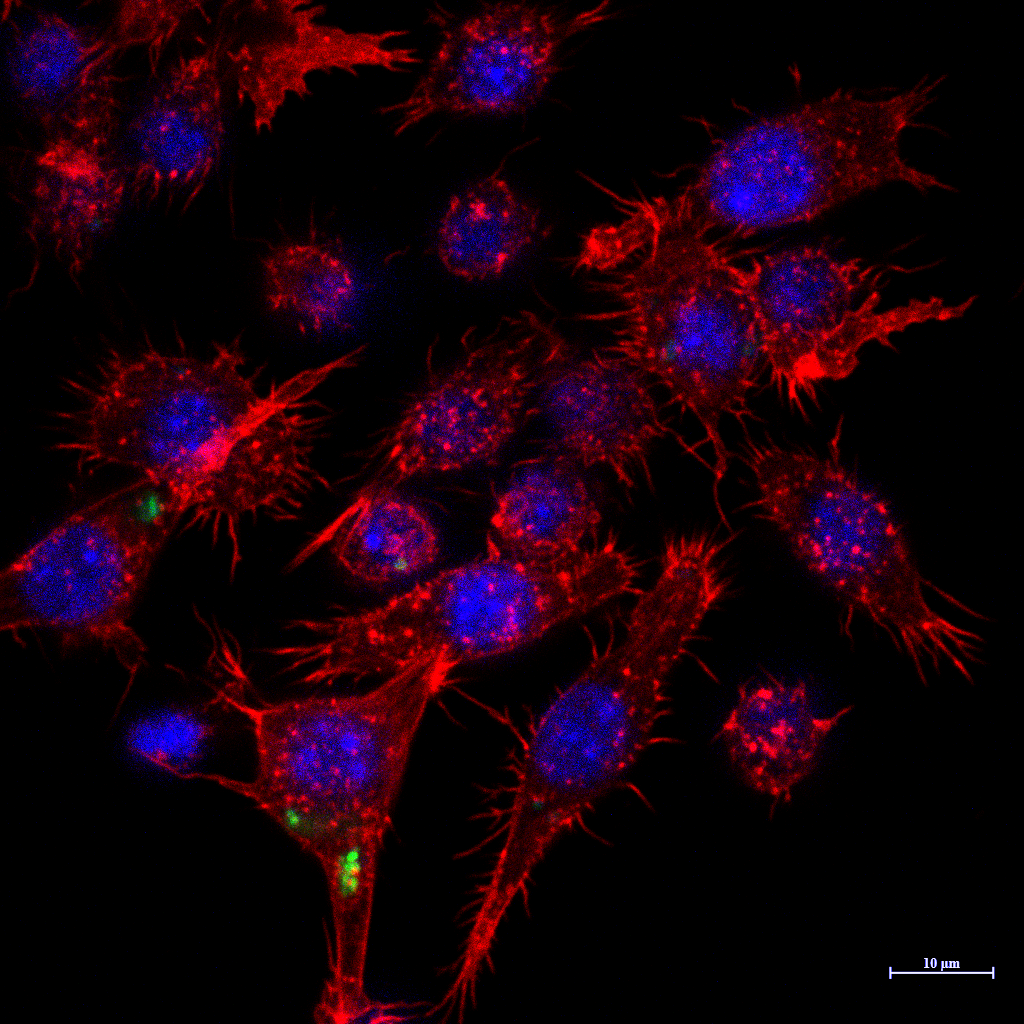

Supplement: Supplementary file 6 — Source data Fig. 2 [file 44318_2024_76_MOESM6_ESM.zip › Figure2/2G/WT.tif]

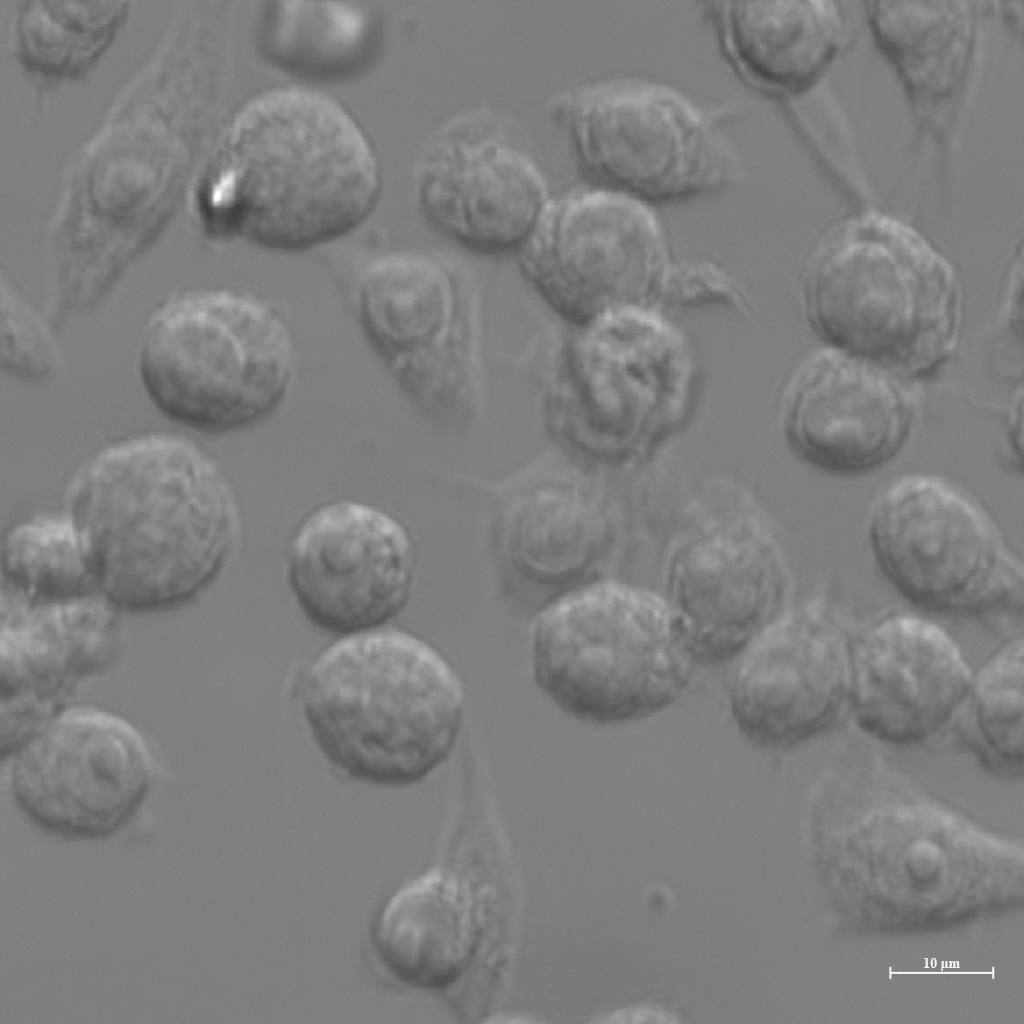

Supplement: Supplementary file 6 — Source data Fig. 2 [file 44318_2024_76_MOESM6_ESM.zip › Figure2/2G/ΔsteC-1.tif]

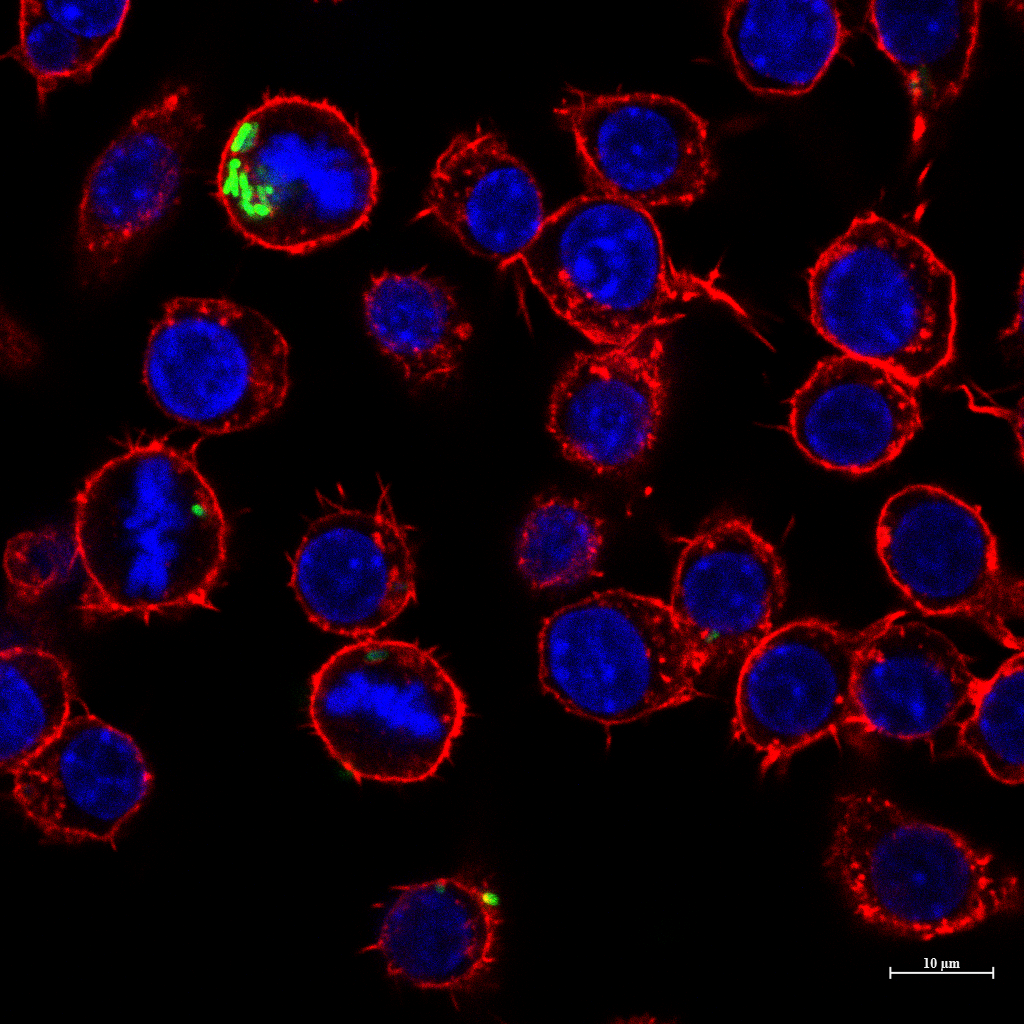

Supplement: Supplementary file 6 — Source data Fig. 2 [file 44318_2024_76_MOESM6_ESM.zip › Figure2/2G/ΔsteC.tif]

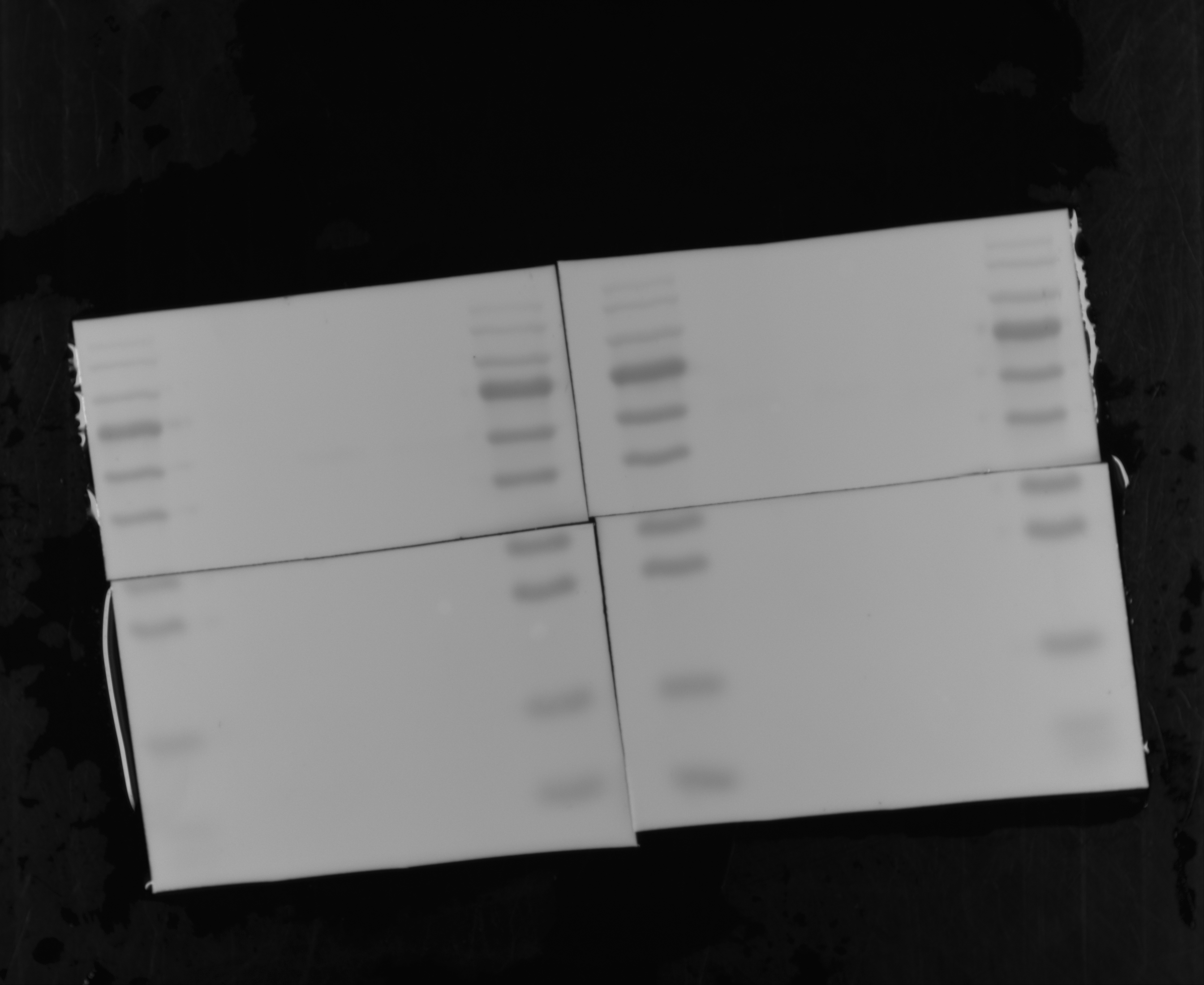

Supplement: Supplementary file 7 — Source data Fig. 3 [file 44318_2024_76_MOESM7_ESM.zip › Figure3/3A/mo.Tif]

Fig.3A

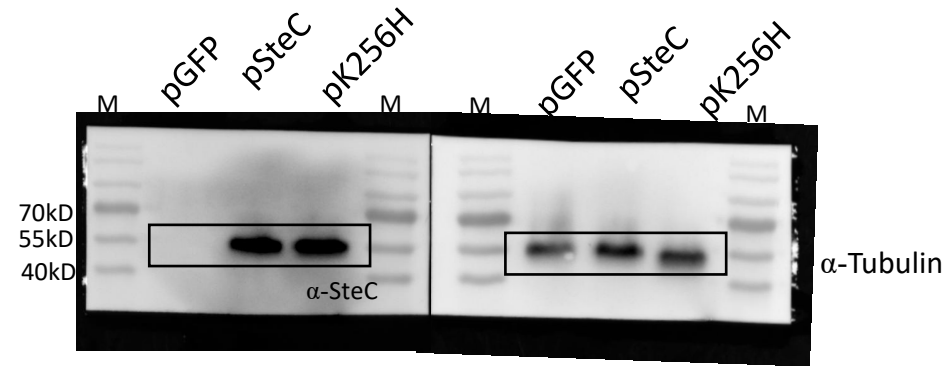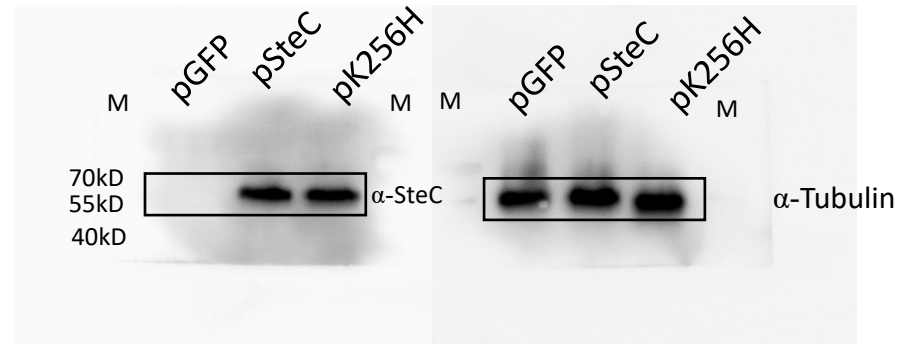

Supplement: Supplementary file 7 — Source data Fig. 3 [file 44318_2024_76_MOESM7_ESM.zip › Figure3/3A/readme.pdf]

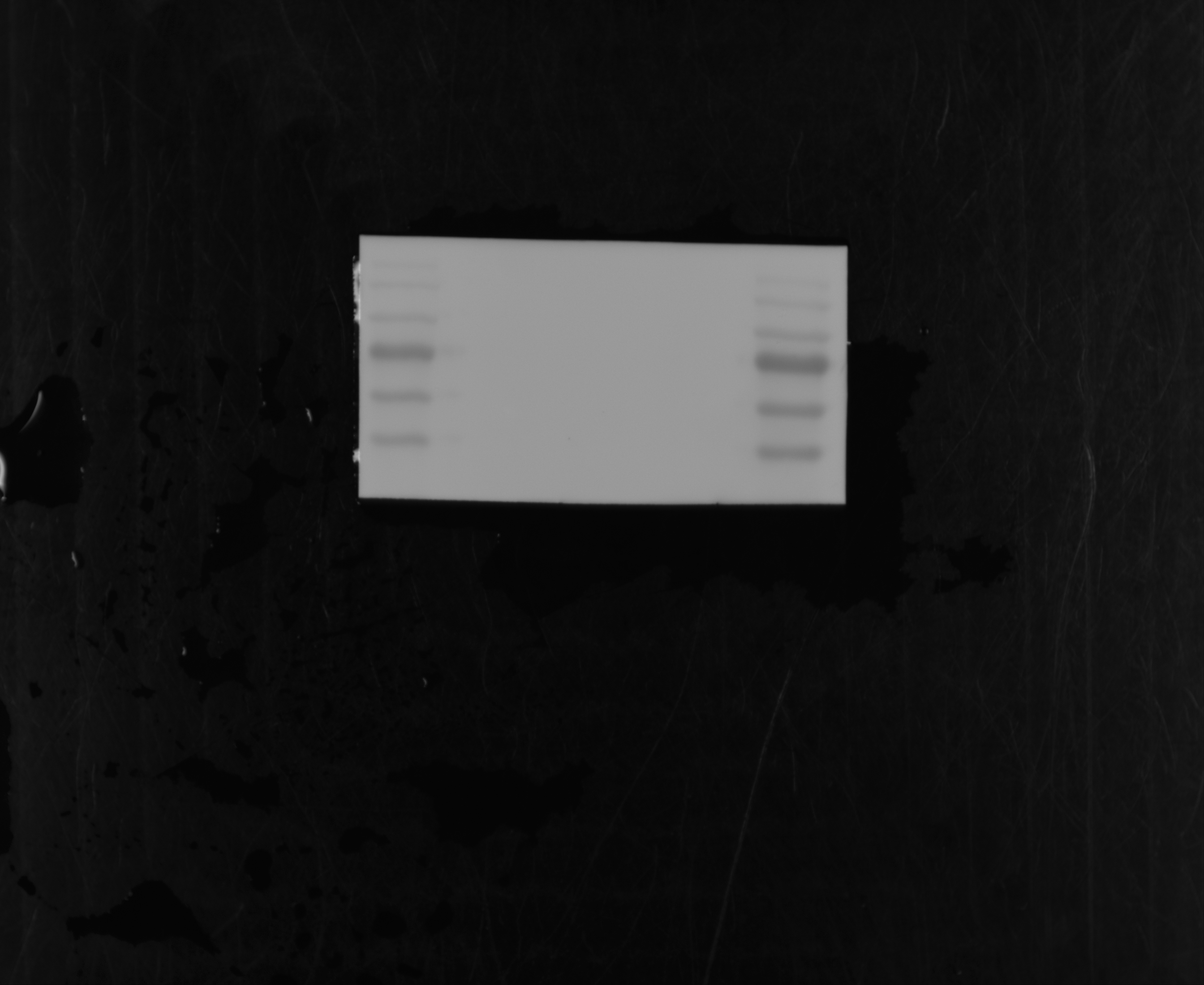

Supplement: Supplementary file 7 — Source data Fig. 3 [file 44318_2024_76_MOESM7_ESM.zip › Figure3/3A/stec 1.Tif]

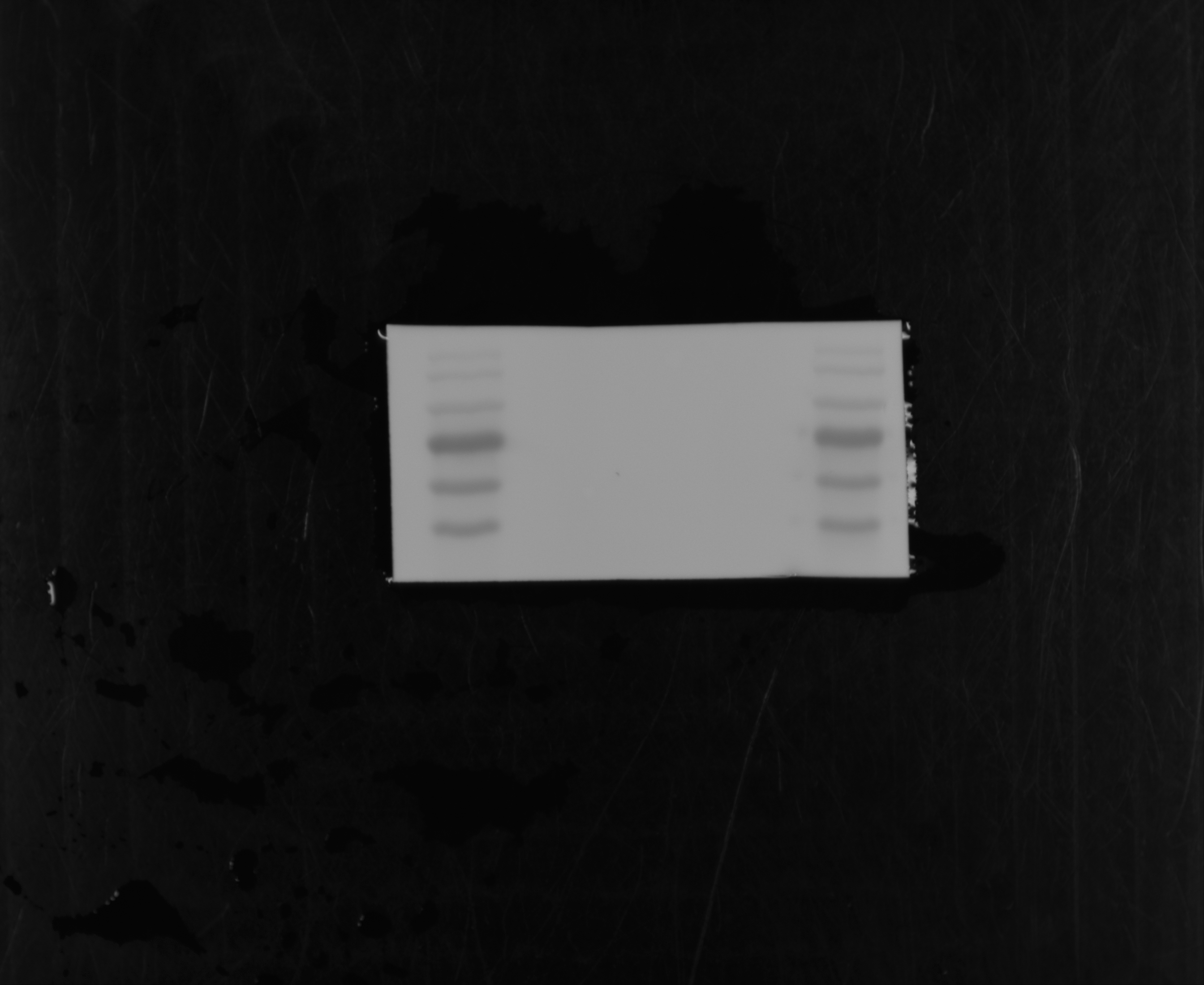

Supplement: Supplementary file 7 — Source data Fig. 3 [file 44318_2024_76_MOESM7_ESM.zip › Figure3/3A/tubulin 1.Tif]

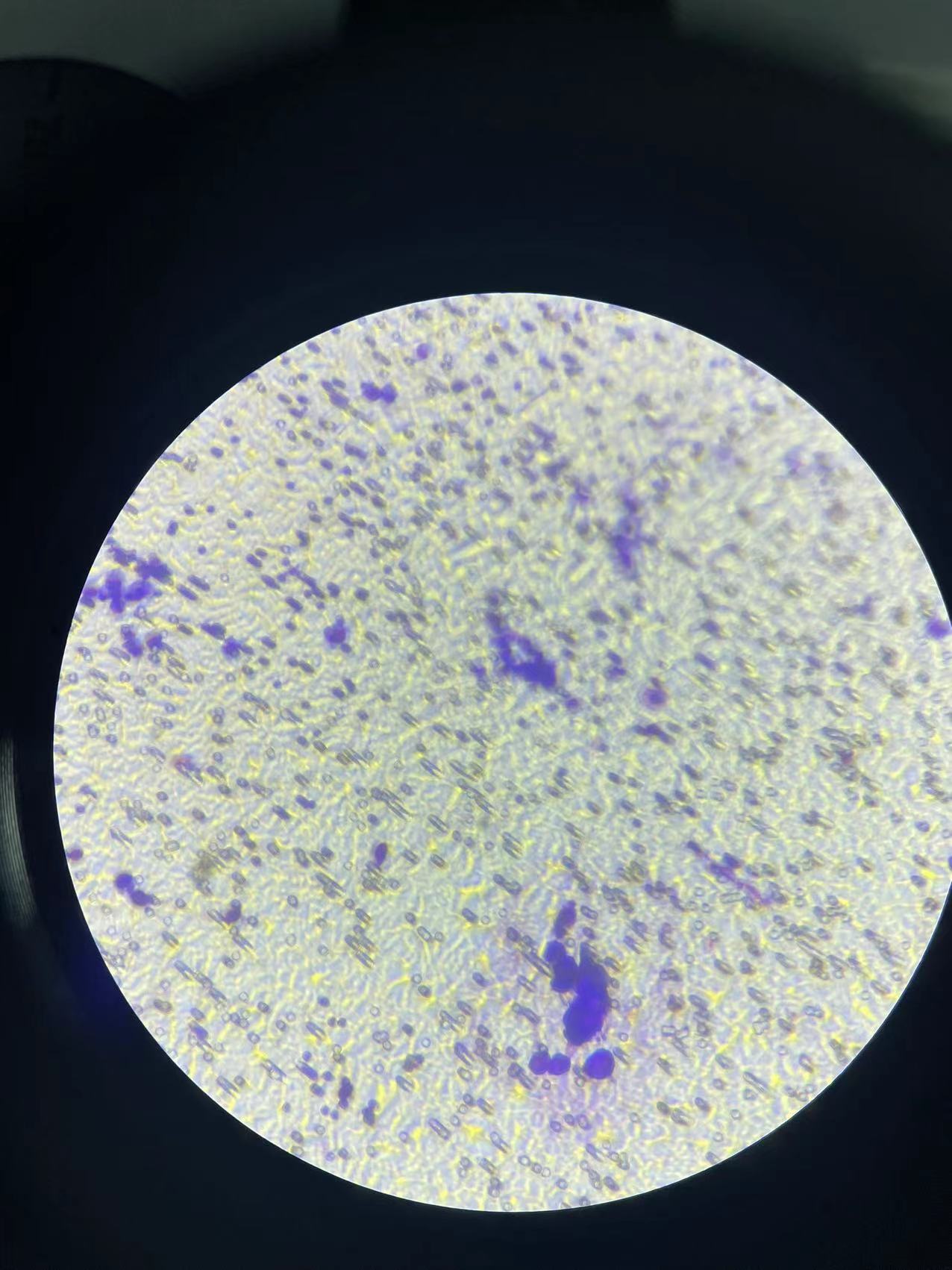

Supplement: Supplementary file 7 — Source data Fig. 3 [file 44318_2024_76_MOESM7_ESM.zip › Figure3/3B/GFP Invasion.jpg]

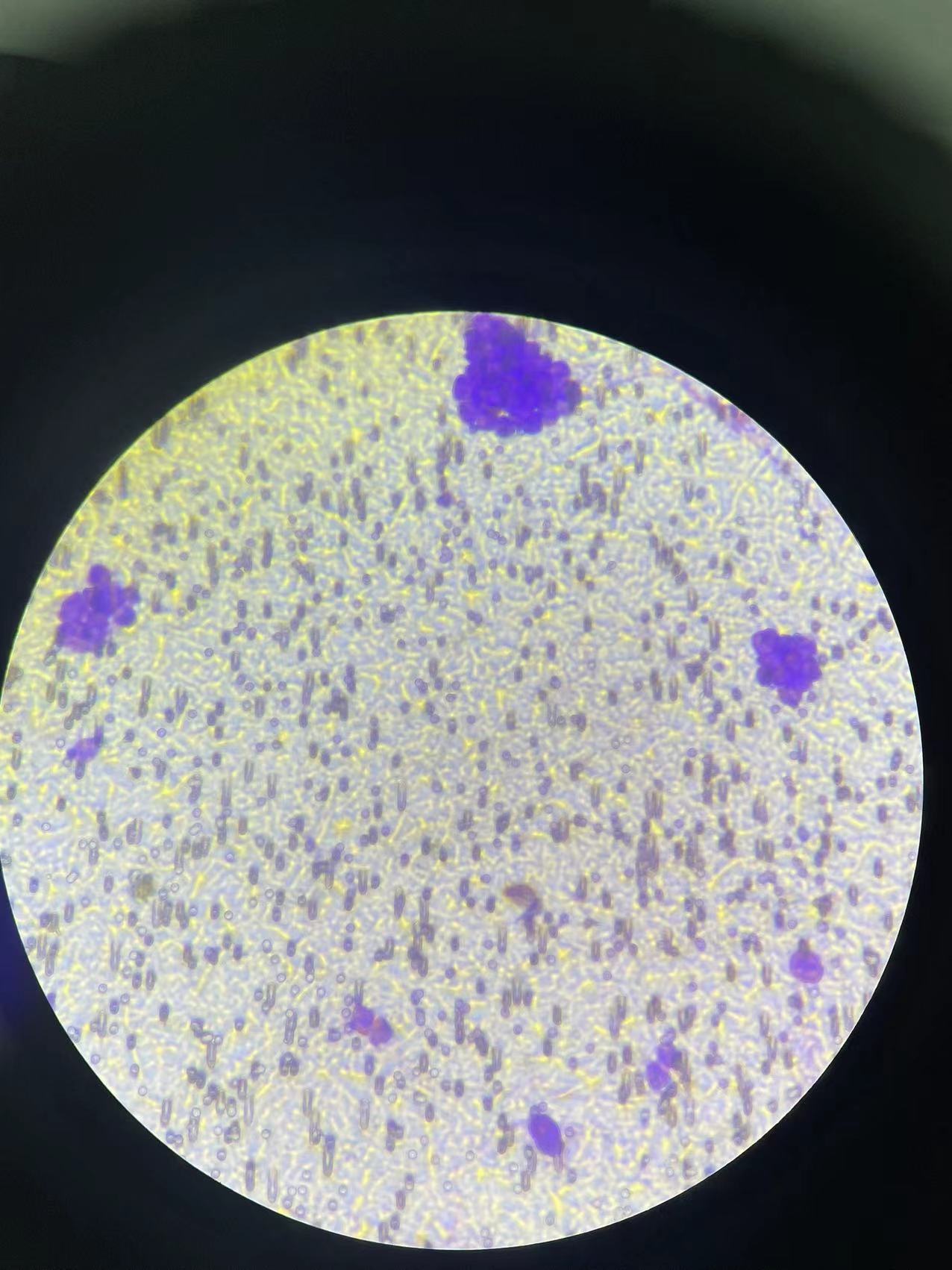

Supplement: Supplementary file 7 — Source data Fig. 3 [file 44318_2024_76_MOESM7_ESM.zip › Figure3/3B/GFP Migration.jpg]

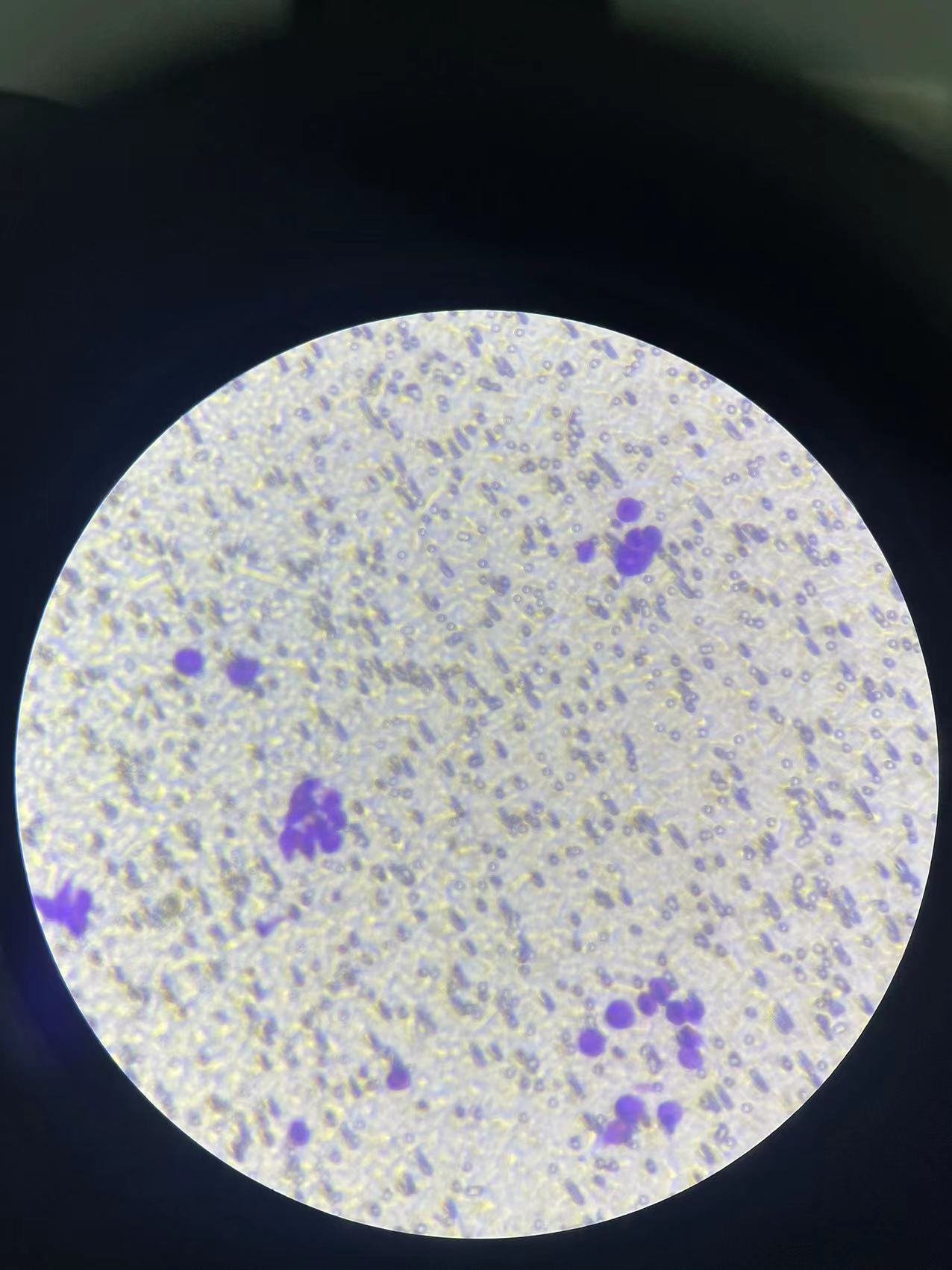

Supplement: Supplementary file 7 — Source data Fig. 3 [file 44318_2024_76_MOESM7_ESM.zip › Figure3/3B/K256H Invasion.jpg]

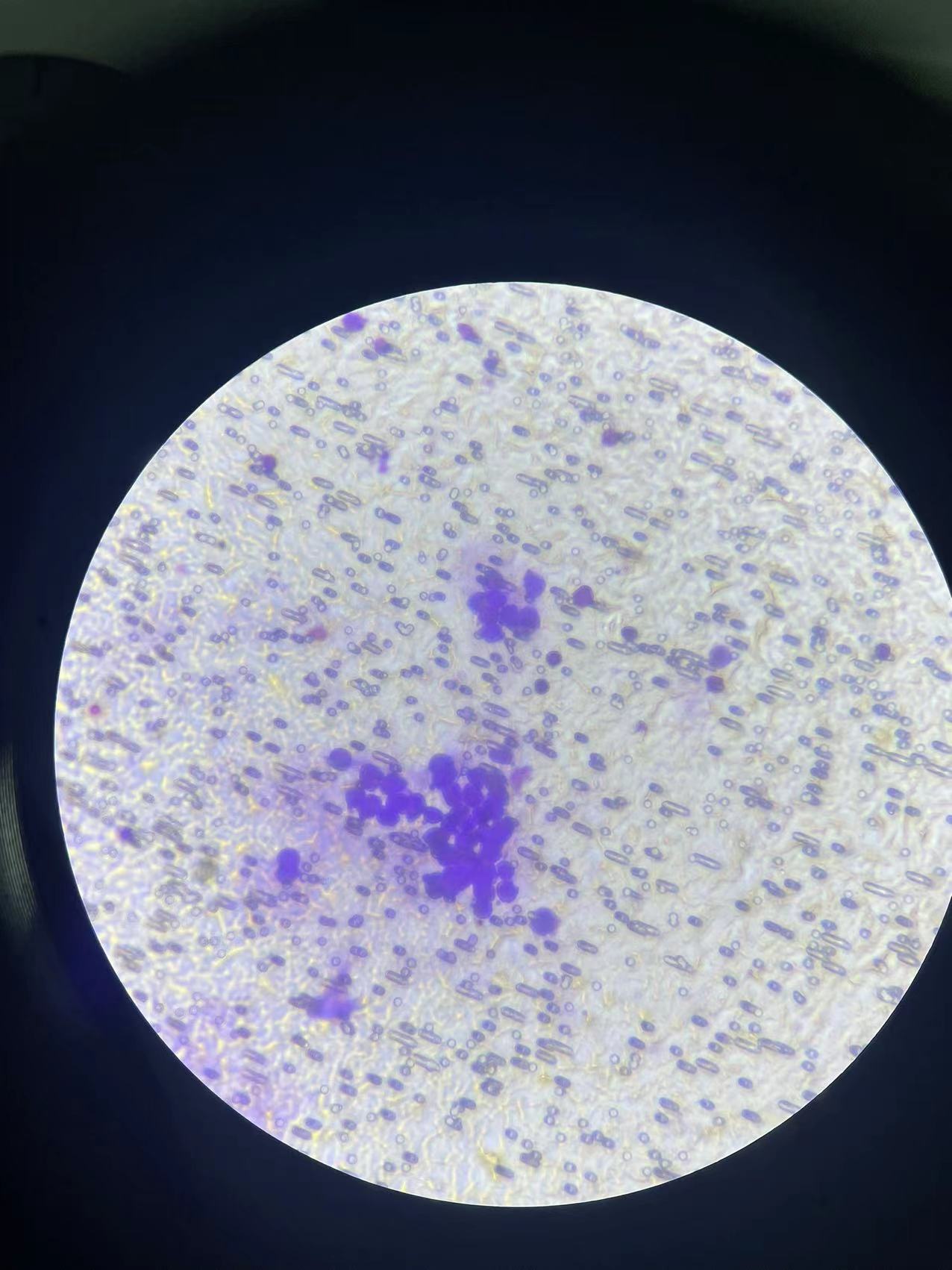

Supplement: Supplementary file 7 — Source data Fig. 3 [file 44318_2024_76_MOESM7_ESM.zip › Figure3/3B/K256H Migration.jpg]

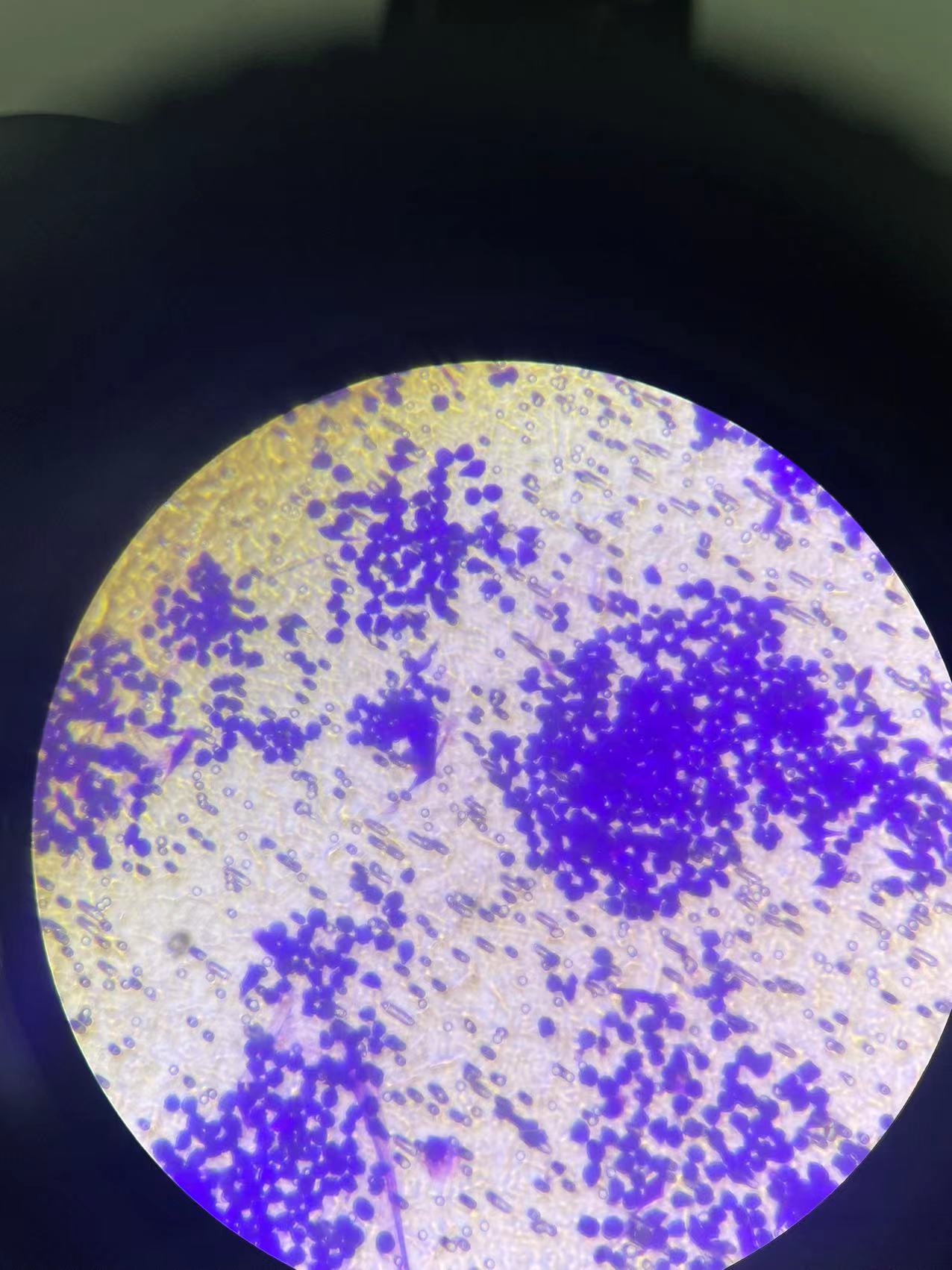

Supplement: Supplementary file 7 — Source data Fig. 3 [file 44318_2024_76_MOESM7_ESM.zip › Figure3/3B/SteC Invasion.jpg]

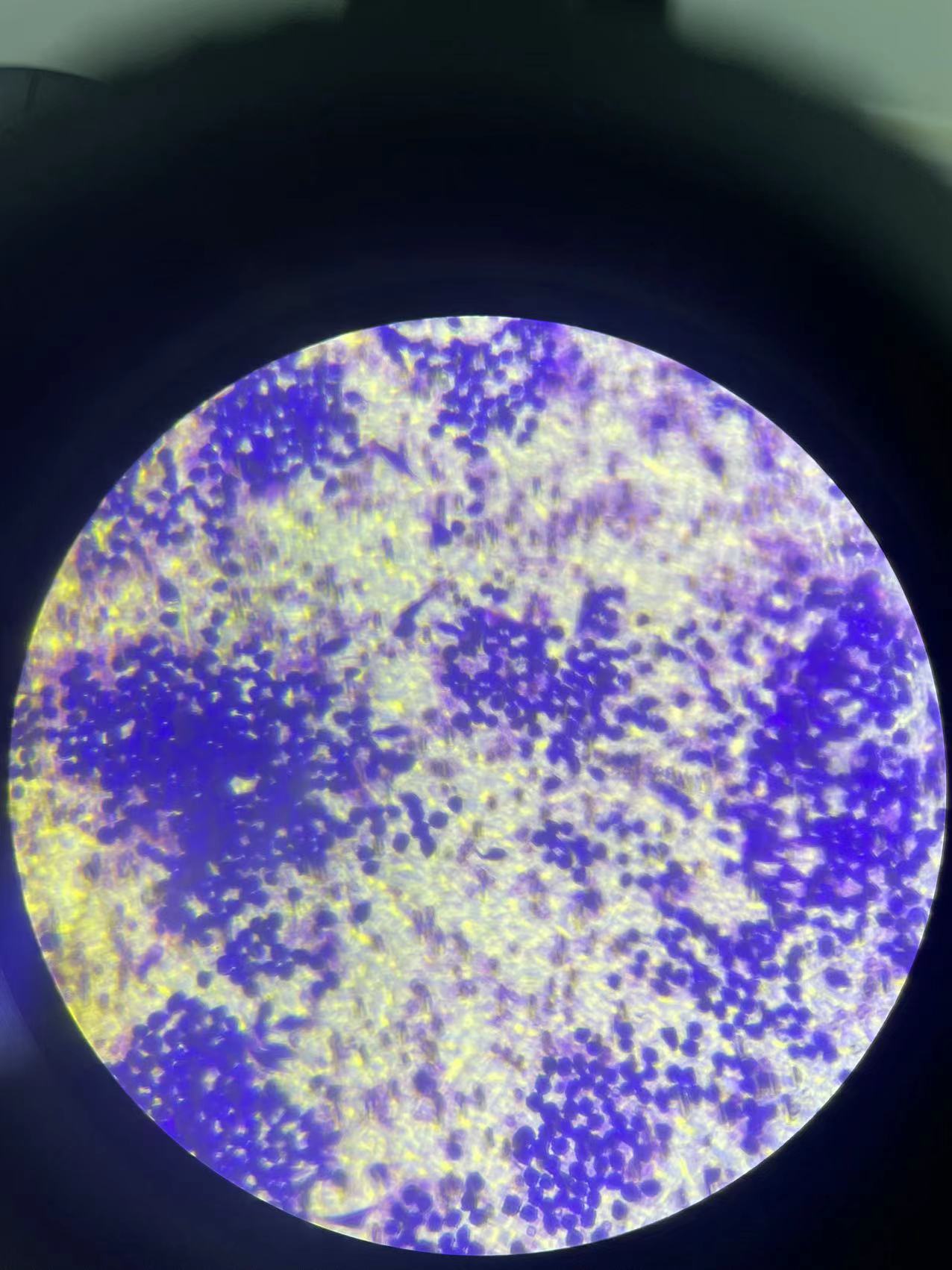

Supplement: Supplementary file 7 — Source data Fig. 3 [file 44318_2024_76_MOESM7_ESM.zip › Figure3/3B/SteC Migration.jpg]

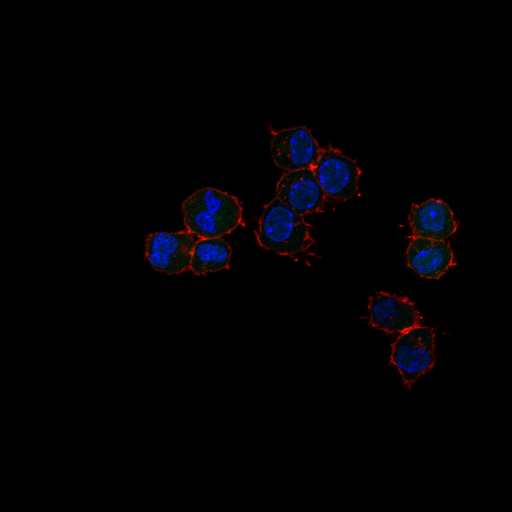

Supplement: Supplementary file 7 — Source data Fig. 3 [file 44318_2024_76_MOESM7_ESM.zip › Figure3/3E/pGFP.tif]

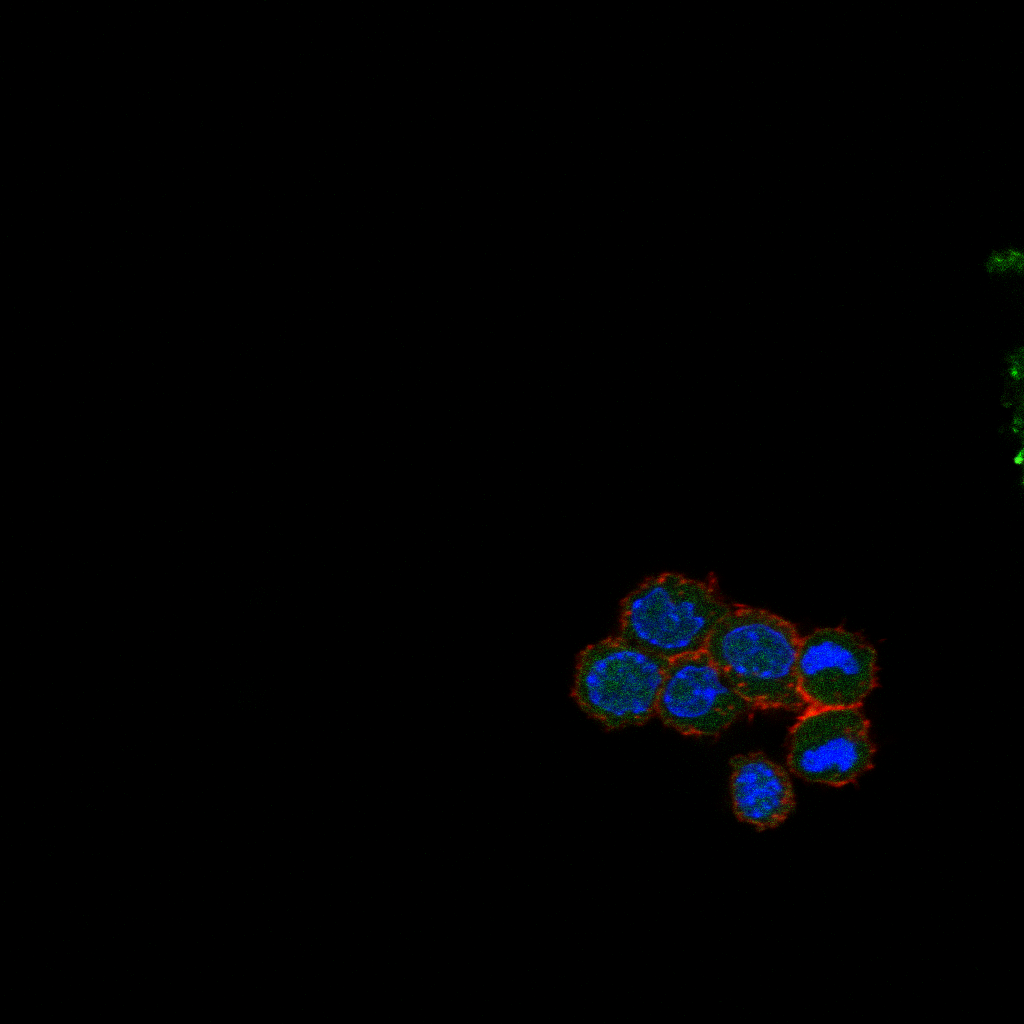

Supplement: Supplementary file 7 — Source data Fig. 3 [file 44318_2024_76_MOESM7_ESM.zip › Figure3/3E/pSteC K256H.tif]

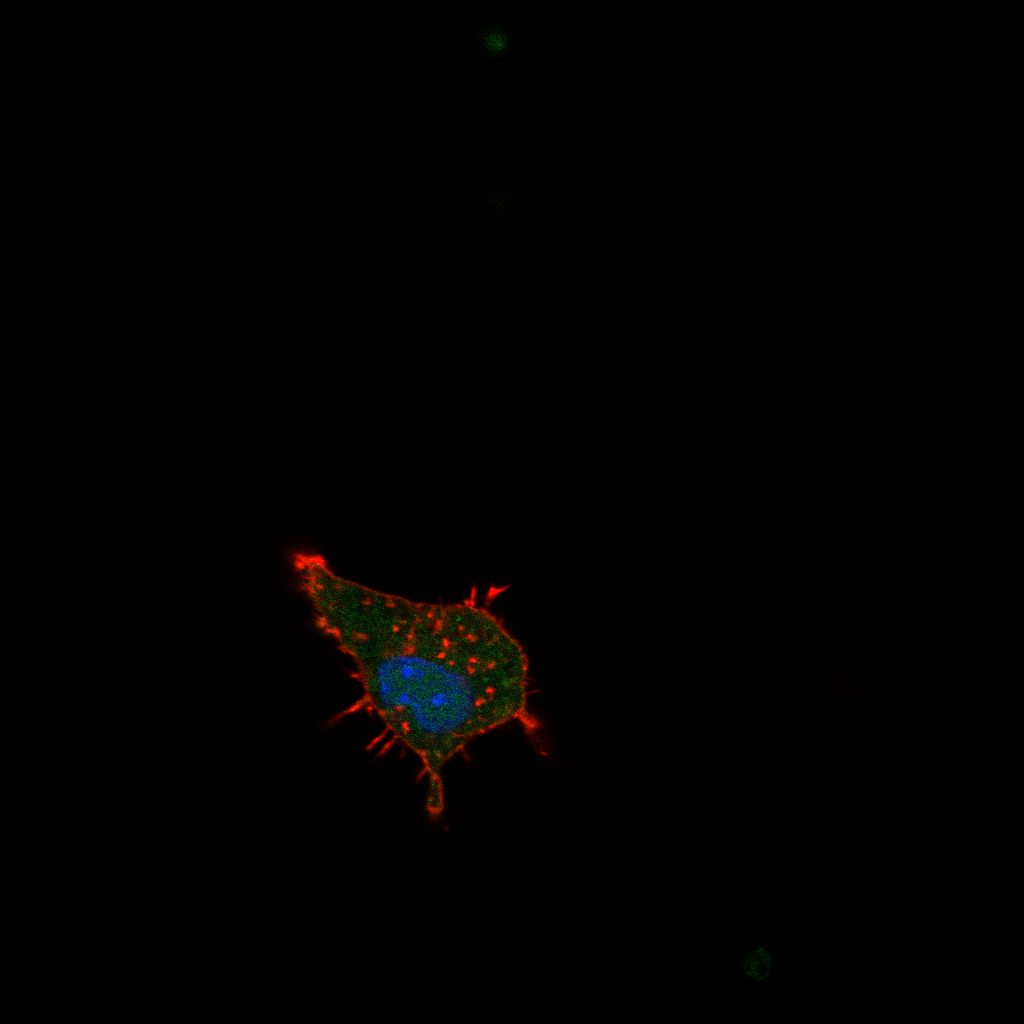

Supplement: Supplementary file 7 — Source data Fig. 3 [file 44318_2024_76_MOESM7_ESM.zip › Figure3/3E/pSteC.tif]

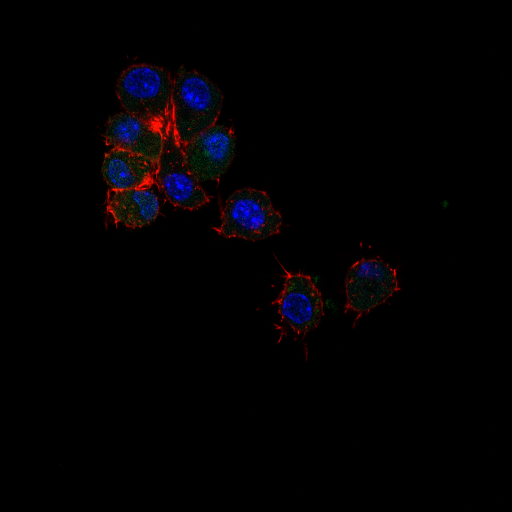

Supplement: Supplementary file 7 — Source data Fig. 3 [file 44318_2024_76_MOESM7_ESM.zip › Figure3/3I/Colchicine.tif]

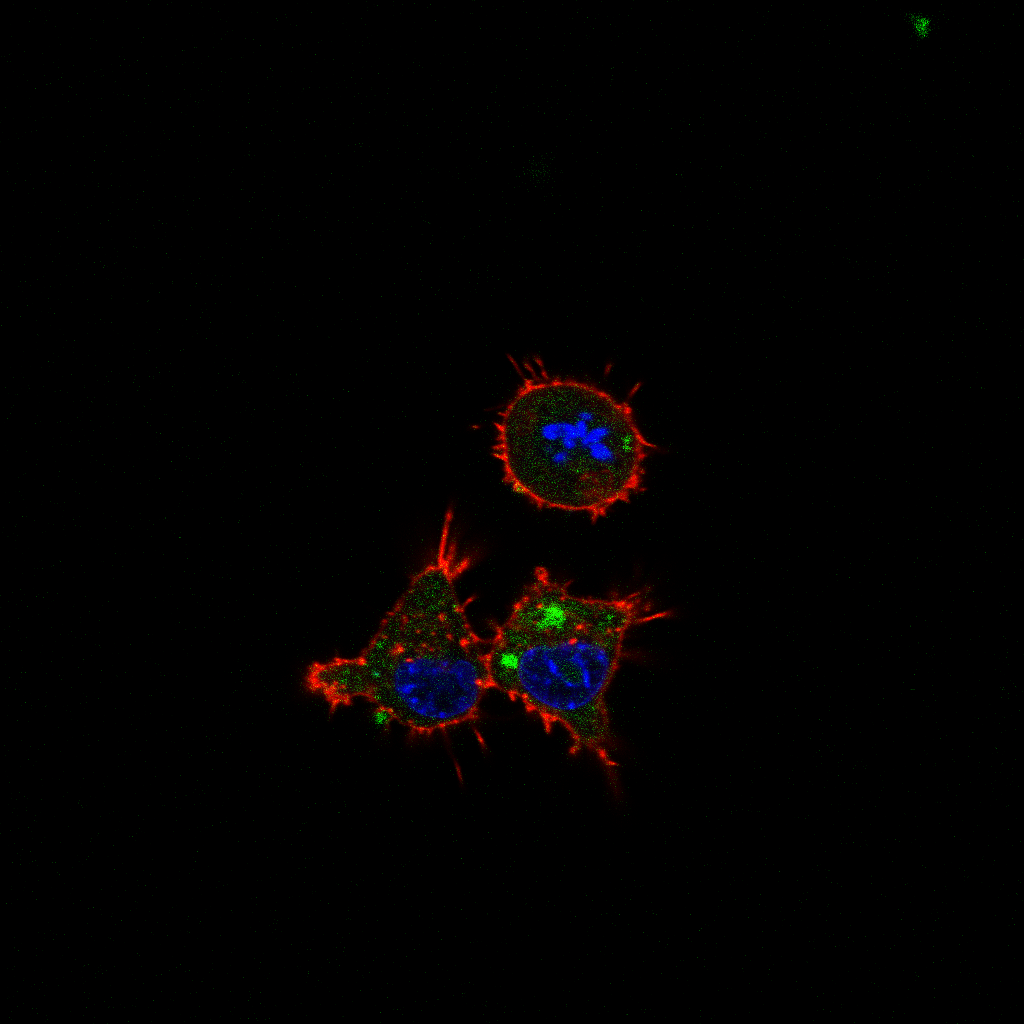

Supplement: Supplementary file 7 — Source data Fig. 3 [file 44318_2024_76_MOESM7_ESM.zip › Figure3/3I/DMSO.tif]

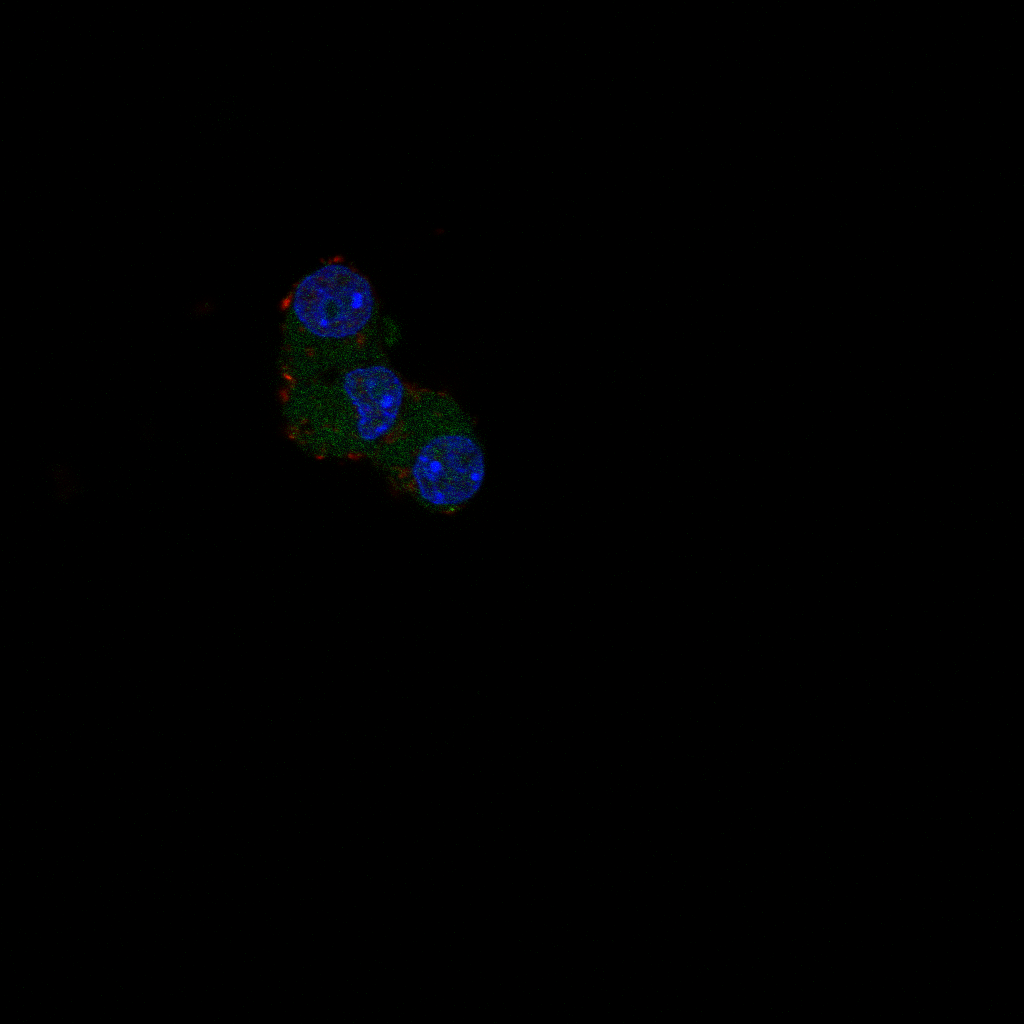

Supplement: Supplementary file 7 — Source data Fig. 3 [file 44318_2024_76_MOESM7_ESM.zip › Figure3/3I/Latrunculin B.tif]

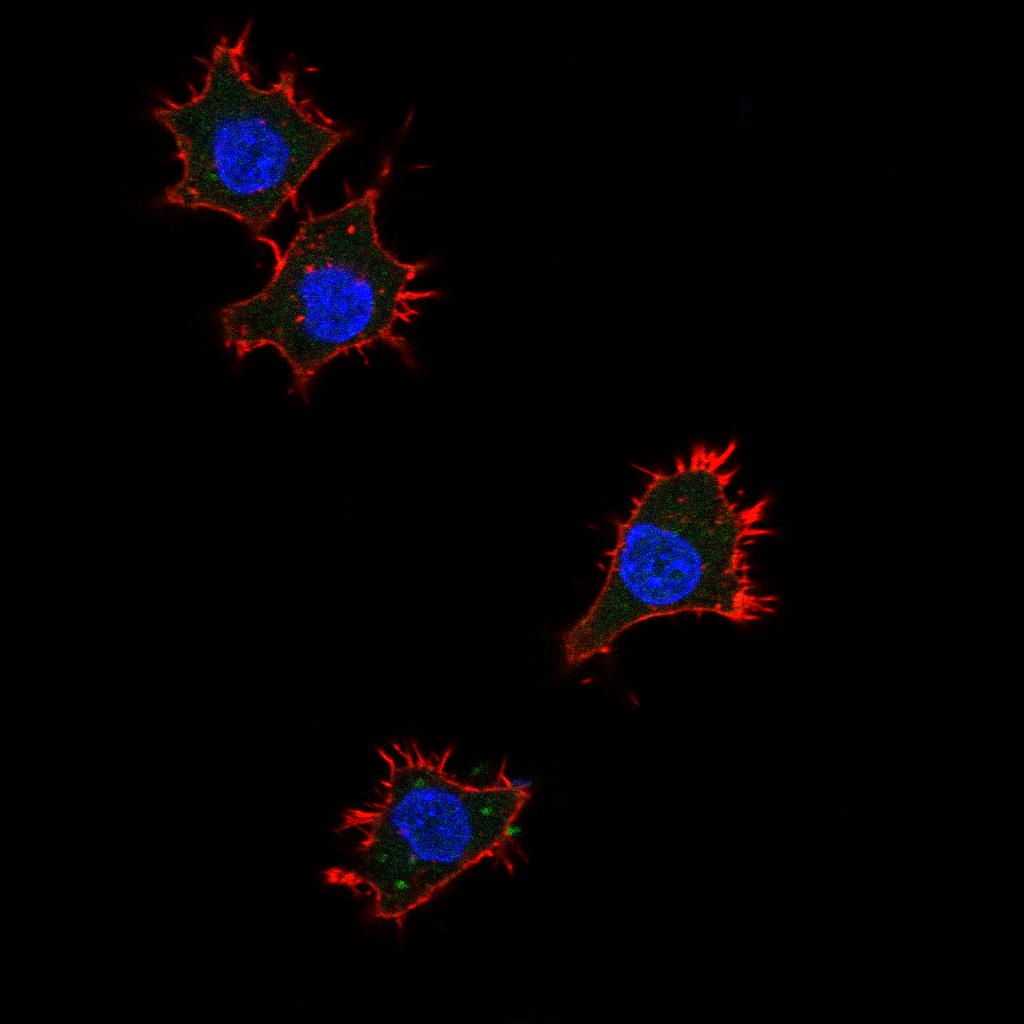

Supplement: Supplementary file 7 — Source data Fig. 3 [file 44318_2024_76_MOESM7_ESM.zip › Figure3/3I/ML-7.tif]

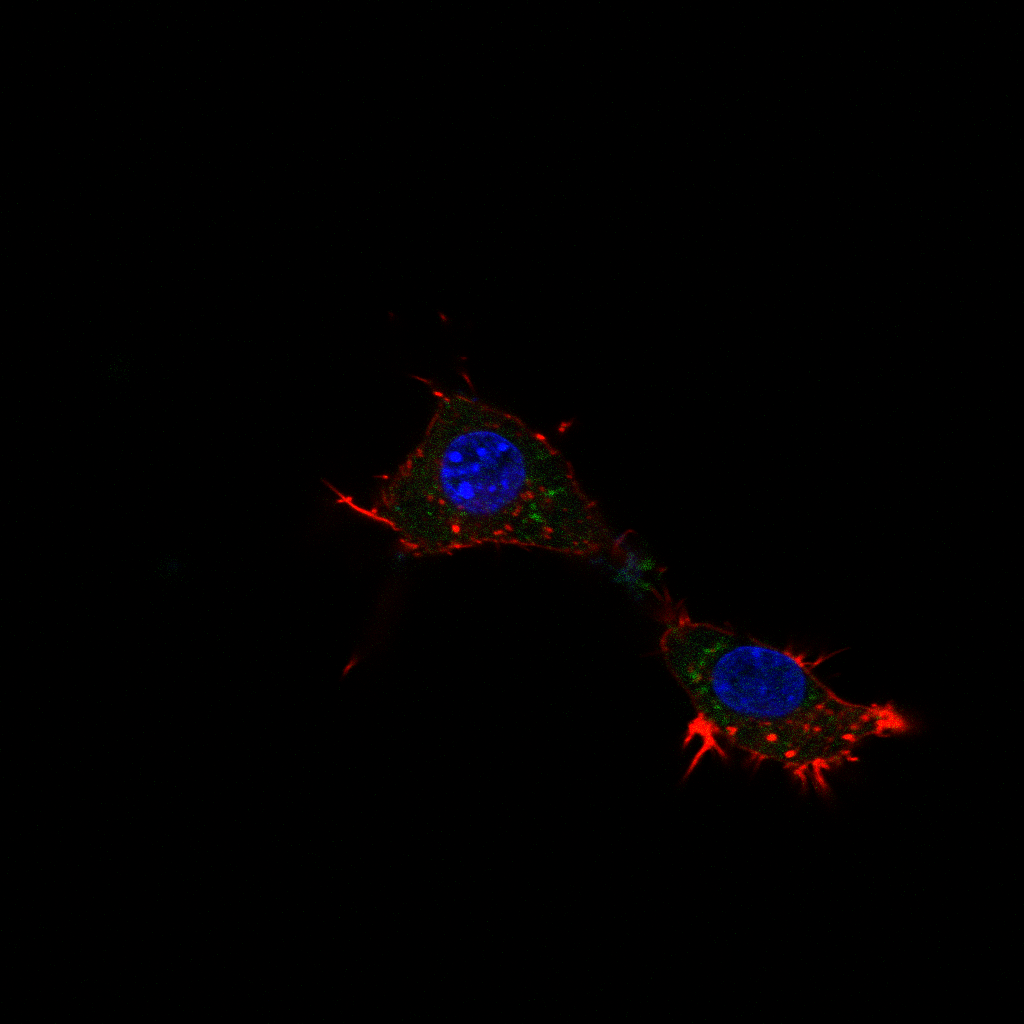

Supplement: Supplementary file 7 — Source data Fig. 3 [file 44318_2024_76_MOESM7_ESM.zip › Figure3/3I/PD-98059.tif]

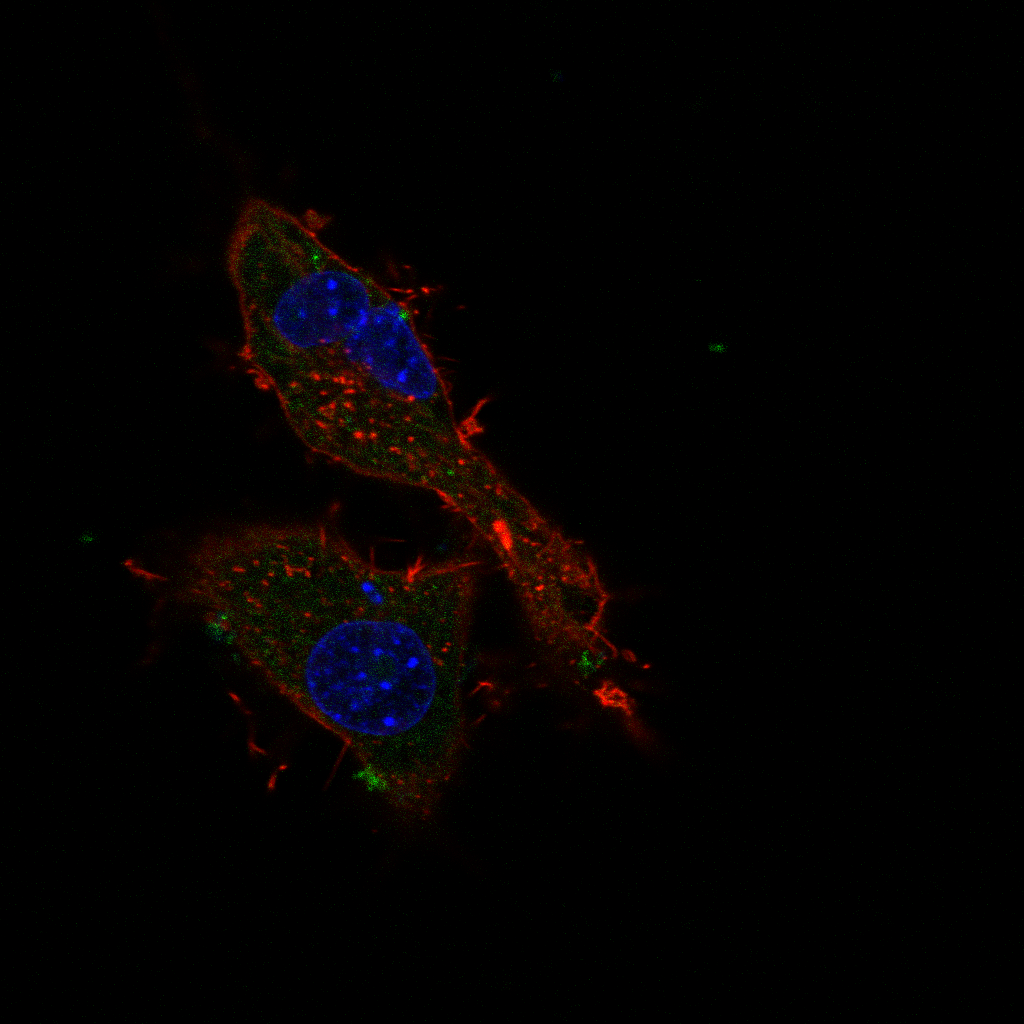

Supplement: Supplementary file 7 — Source data Fig. 3 [file 44318_2024_76_MOESM7_ESM.zip › Figure3/3I/Y-33075.tif]

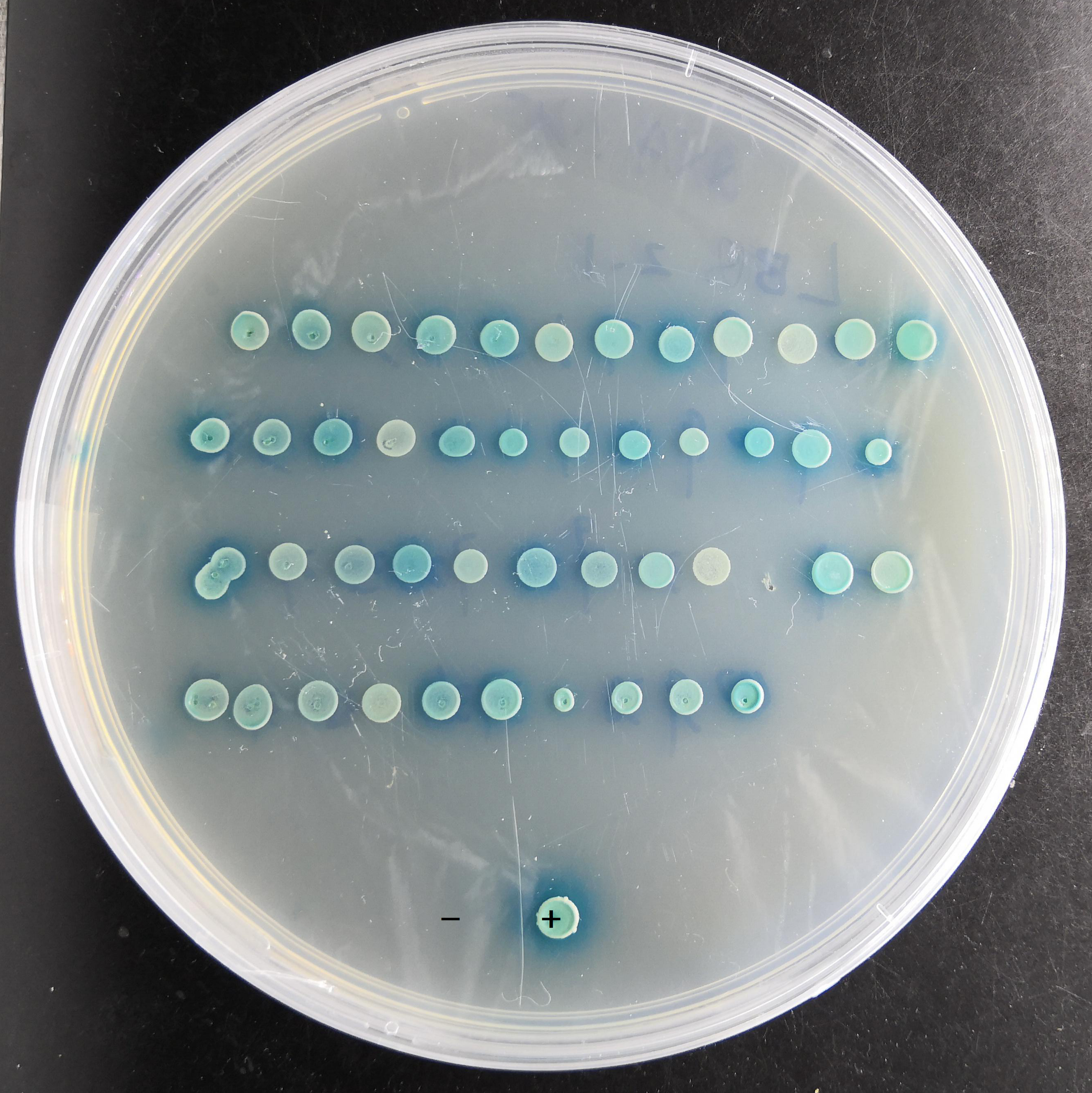

Supplement: Supplementary file 8 — Source data Fig. 4 [file 44318_2024_76_MOESM8_ESM.zip › Figure4/4A/4A.JPG]

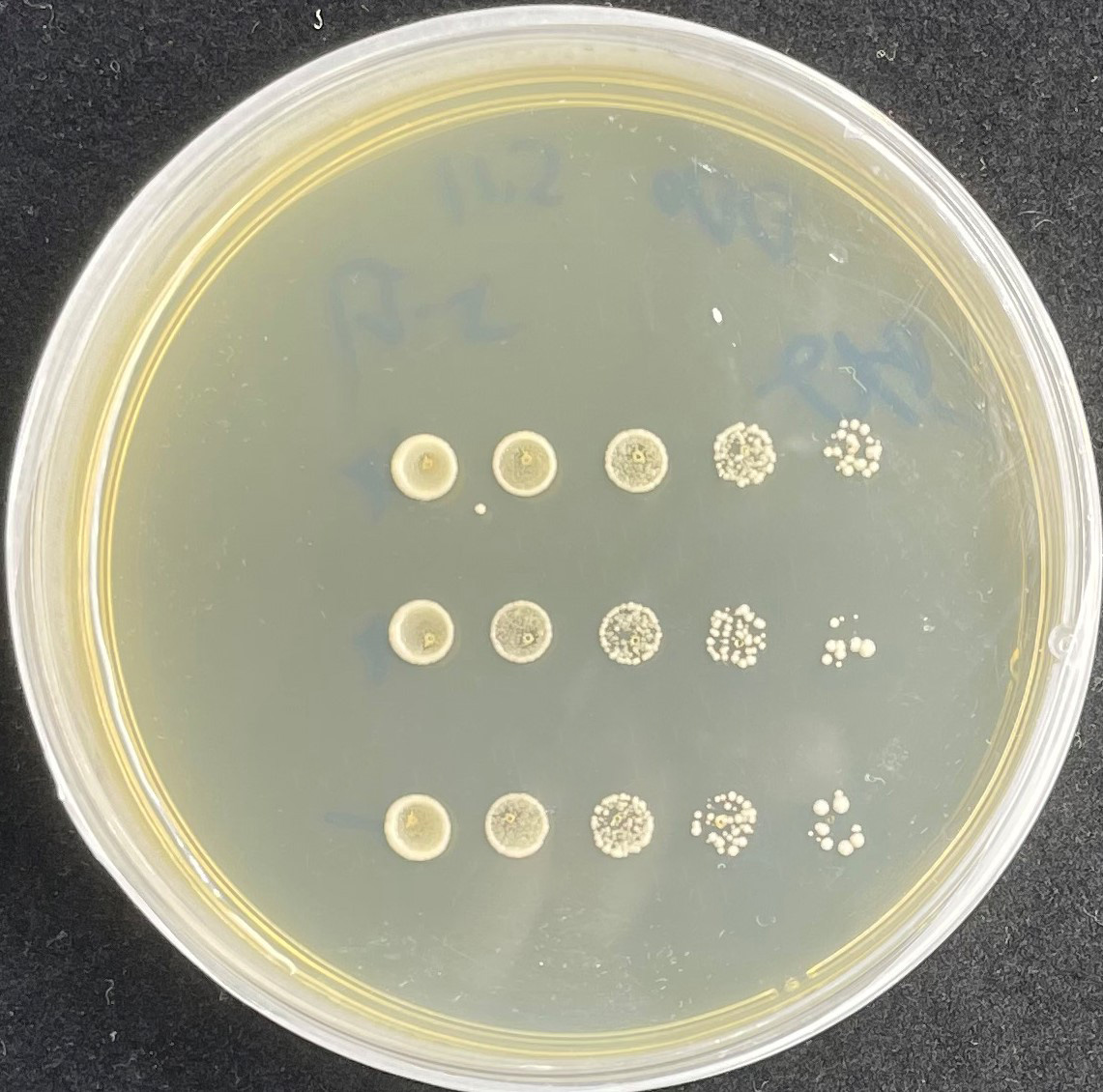

Supplement: Supplementary file 8 — Source data Fig. 4 [file 44318_2024_76_MOESM8_ESM.zip › Figure4/4B/4B-1.JPG]

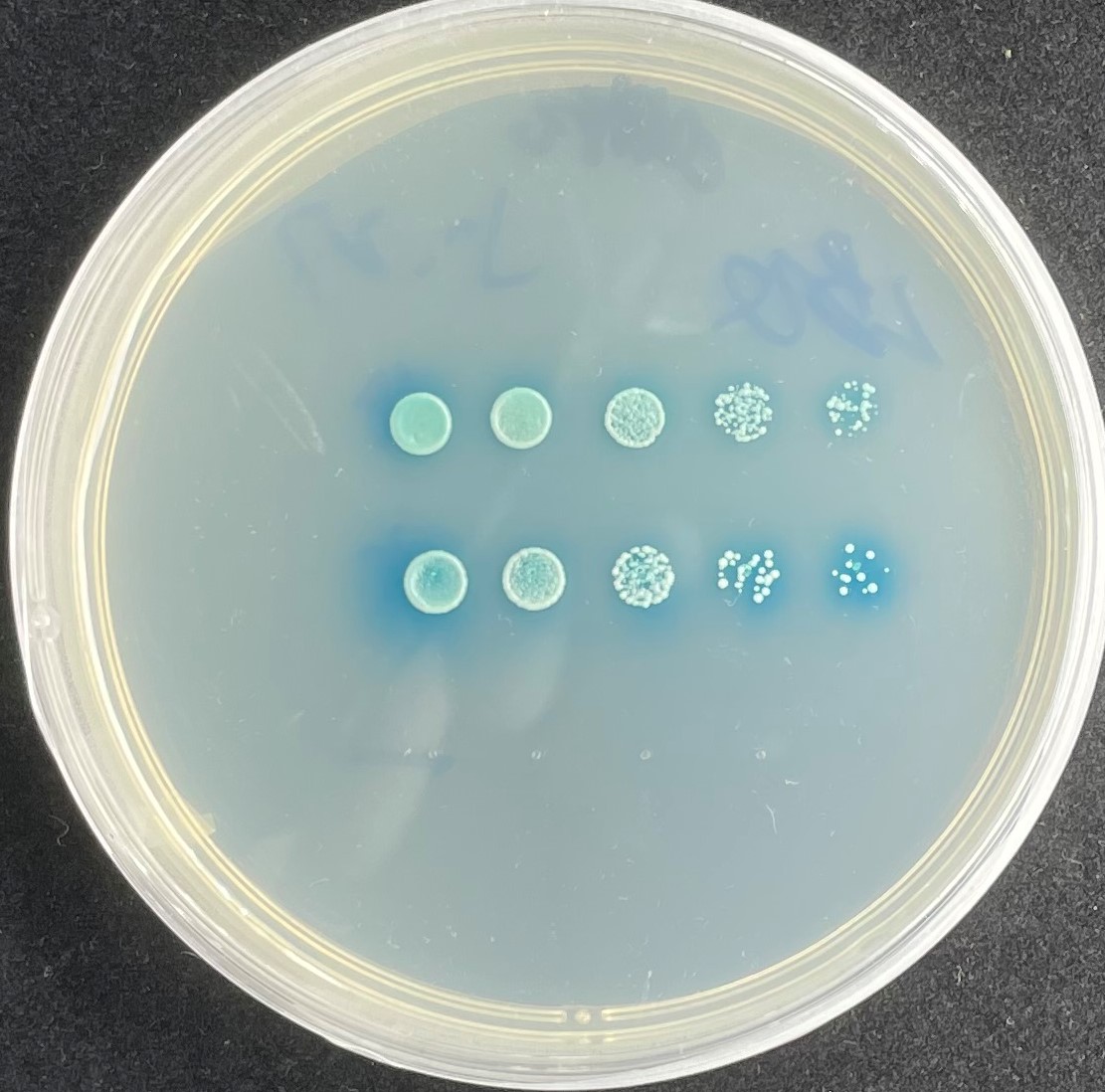

Supplement: Supplementary file 8 — Source data Fig. 4 [file 44318_2024_76_MOESM8_ESM.zip › Figure4/4B/4B-2.JPG]

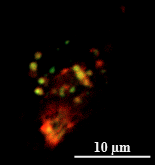

Supplement: Supplementary file 8 — Source data Fig. 4 [file 44318_2024_76_MOESM8_ESM.zip › Figure4/4C/31281-Untitled60_c1-2.tif]

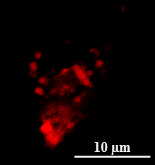

Supplement: Supplementary file 8 — Source data Fig. 4 [file 44318_2024_76_MOESM8_ESM.zip › Figure4/4C/31281-Untitled60_c1.tif]

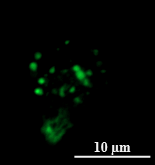

Supplement: Supplementary file 8 — Source data Fig. 4 [file 44318_2024_76_MOESM8_ESM.zip › Figure4/4C/31281-Untitled60_c2.tif]

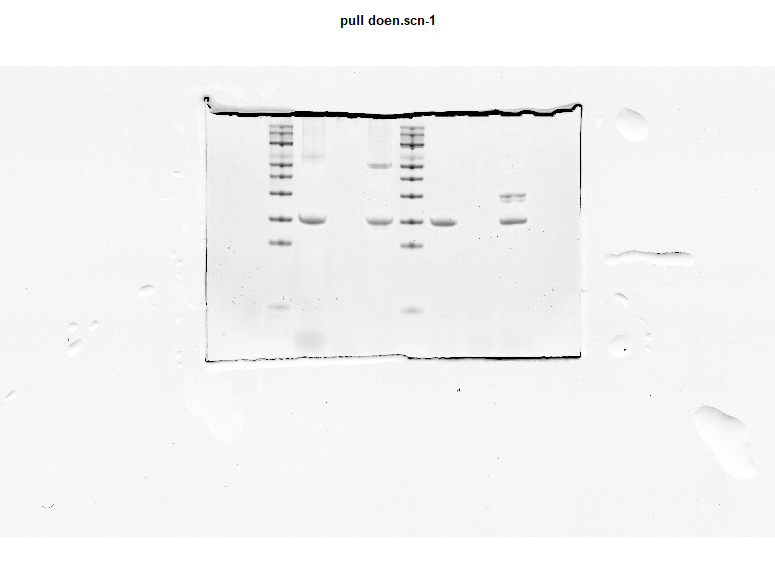

Supplement: Supplementary file 8 — Source data Fig. 4 [file 44318_2024_76_MOESM8_ESM.zip › Figure4/4F/4F.tif]

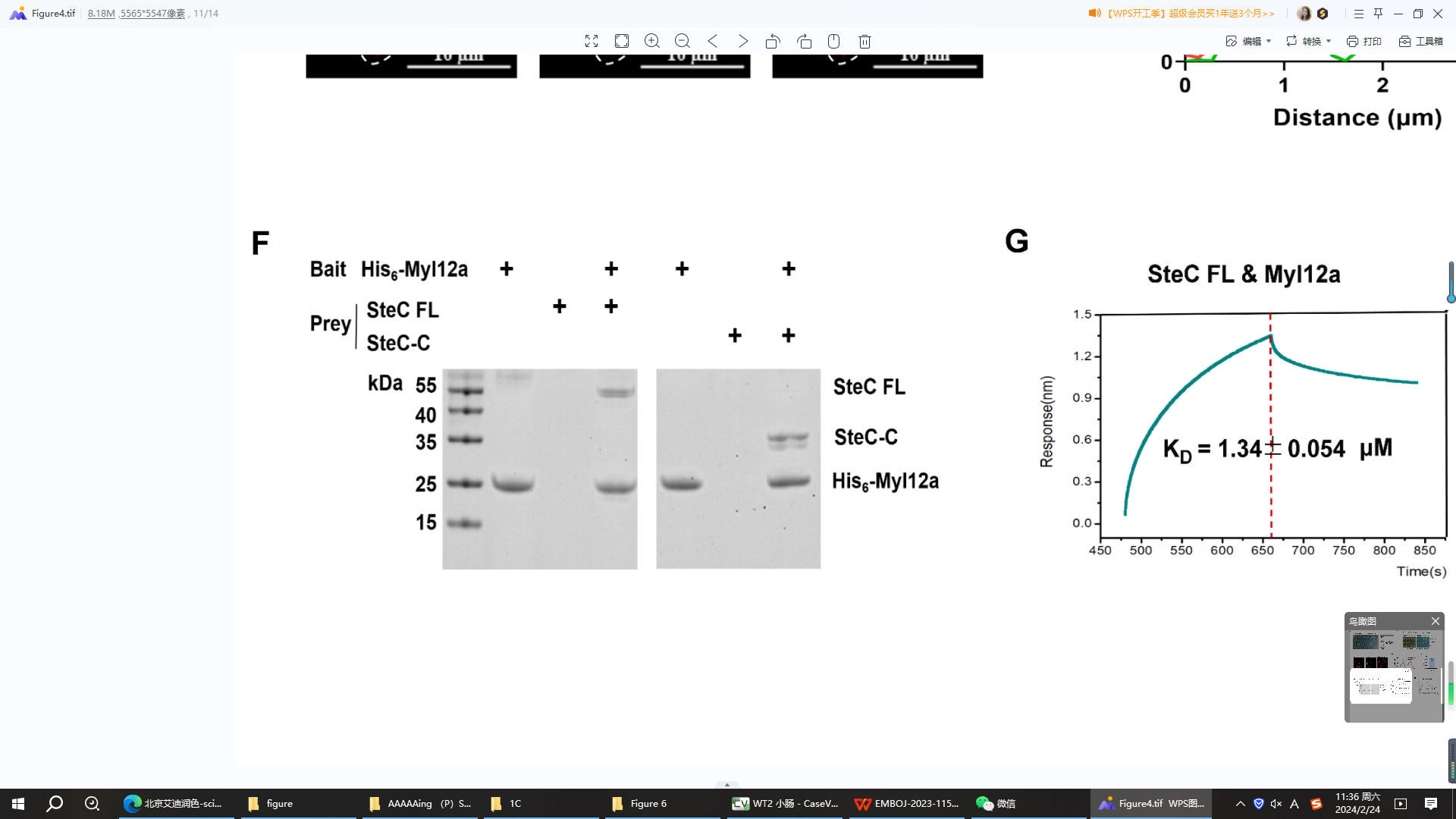


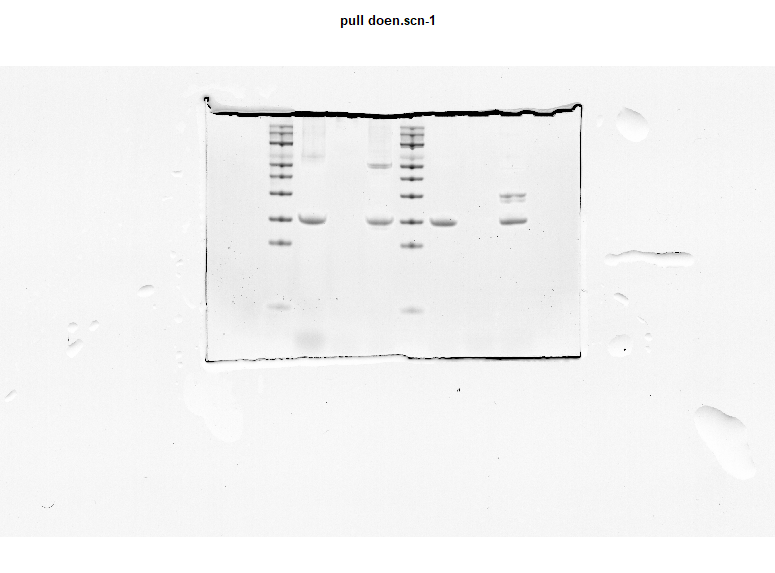

Supplement: Supplementary file 8 — Source data Fig. 4 [file 44318_2024_76_MOESM8_ESM.zip › Figure4/4F/READ ME.docx]

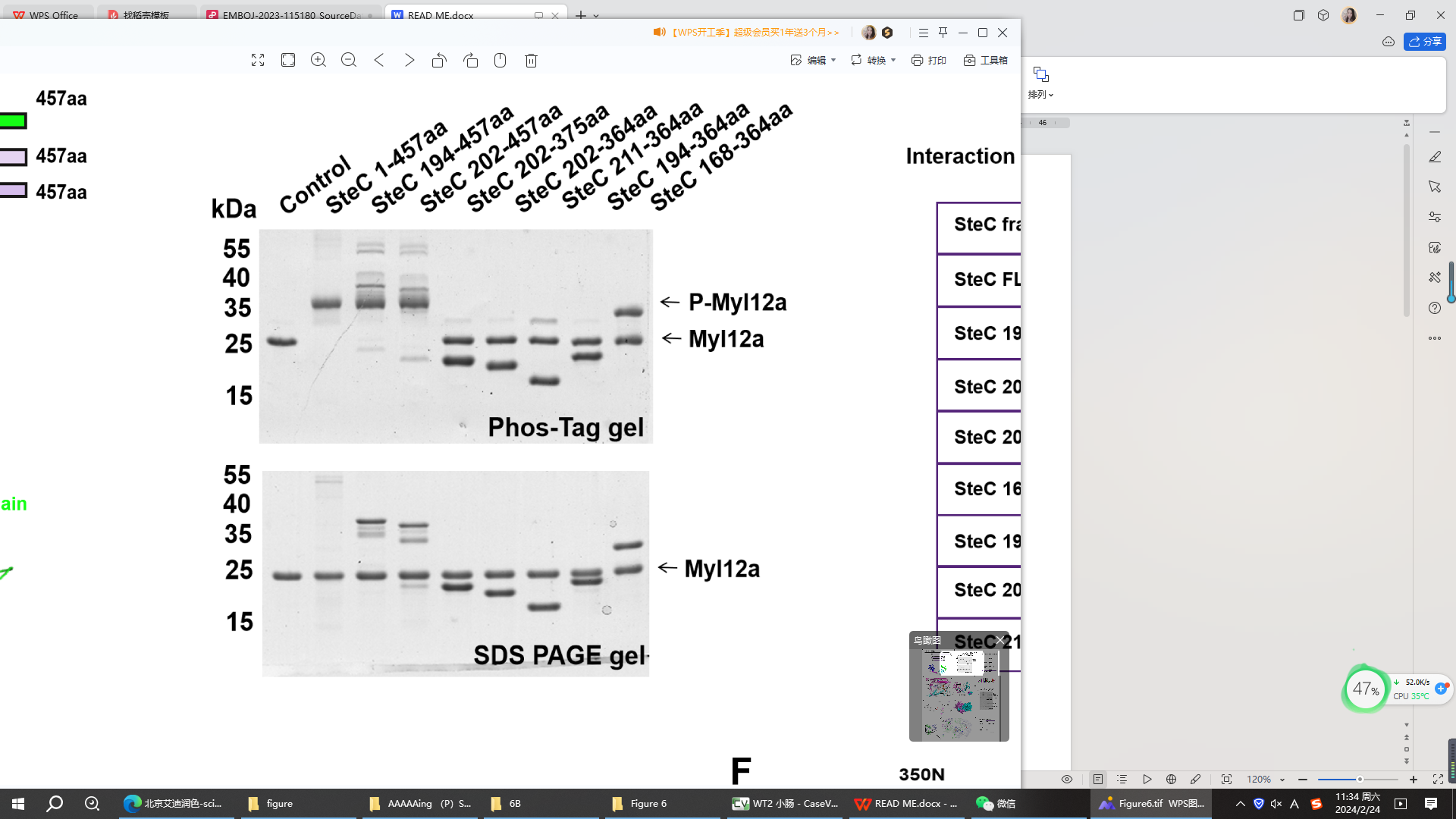


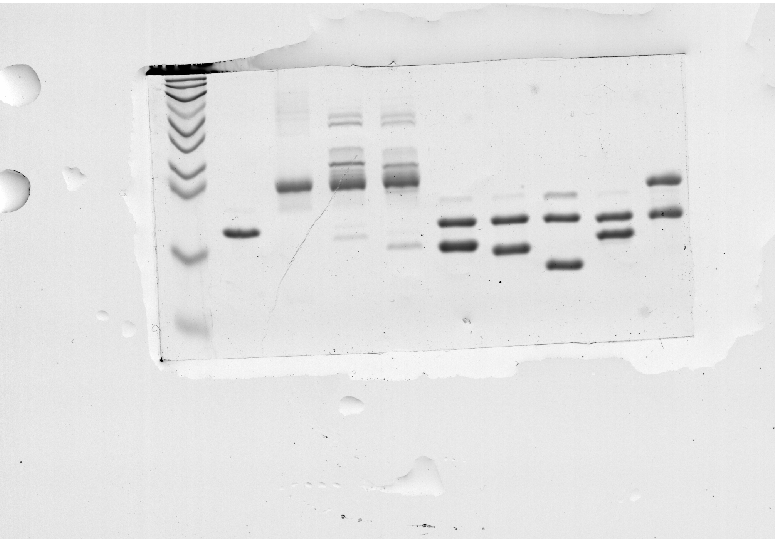


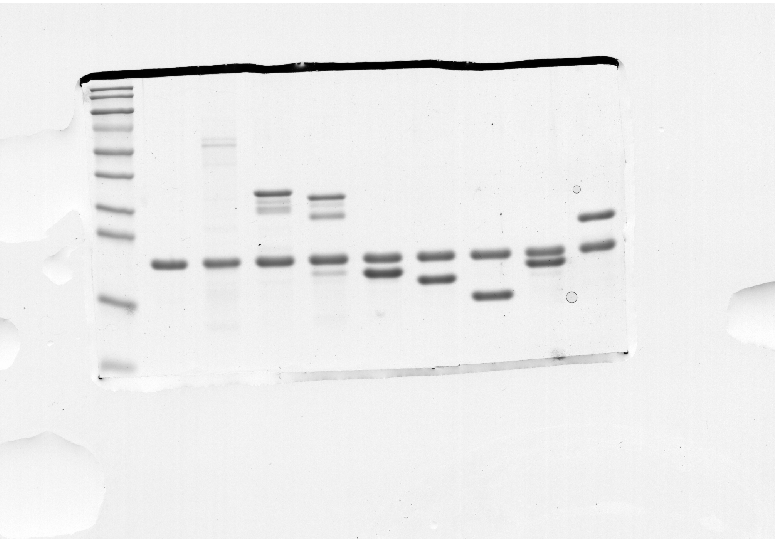

Supplement: Supplementary file 10 — Source data Fig. 6 [file 44318_2024_76_MOESM10_ESM.zip › Figure6/6B/READ ME.docx]

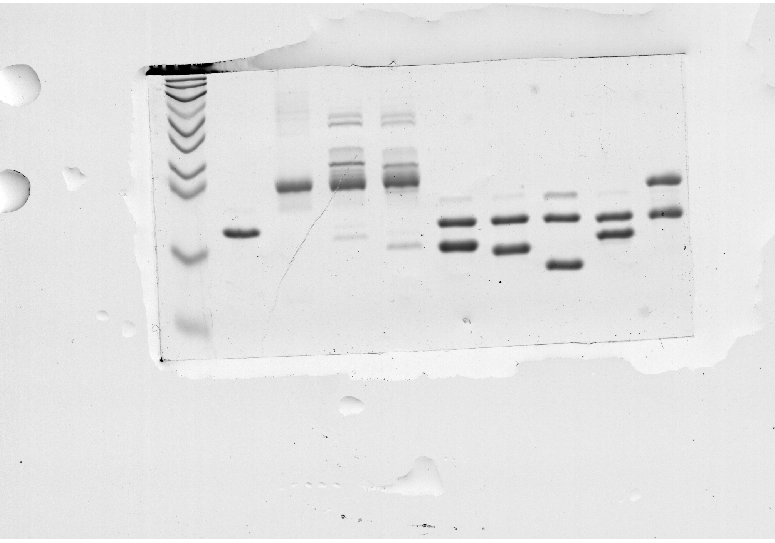

Supplement: Supplementary file 10 — Source data Fig. 6 [file 44318_2024_76_MOESM10_ESM.zip › Figure6/6B/phos tag gel.tif]

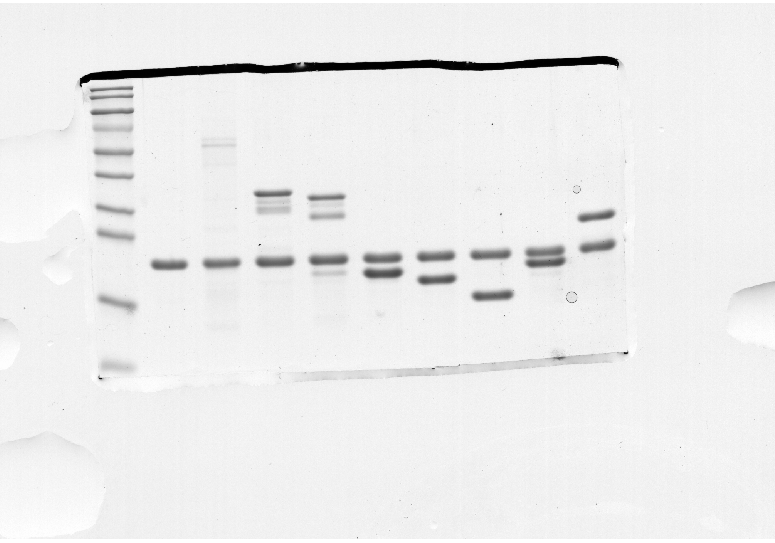

Supplement: Supplementary file 10 — Source data Fig. 6 [file 44318_2024_76_MOESM10_ESM.zip › Figure6/6B/sds page.tif]

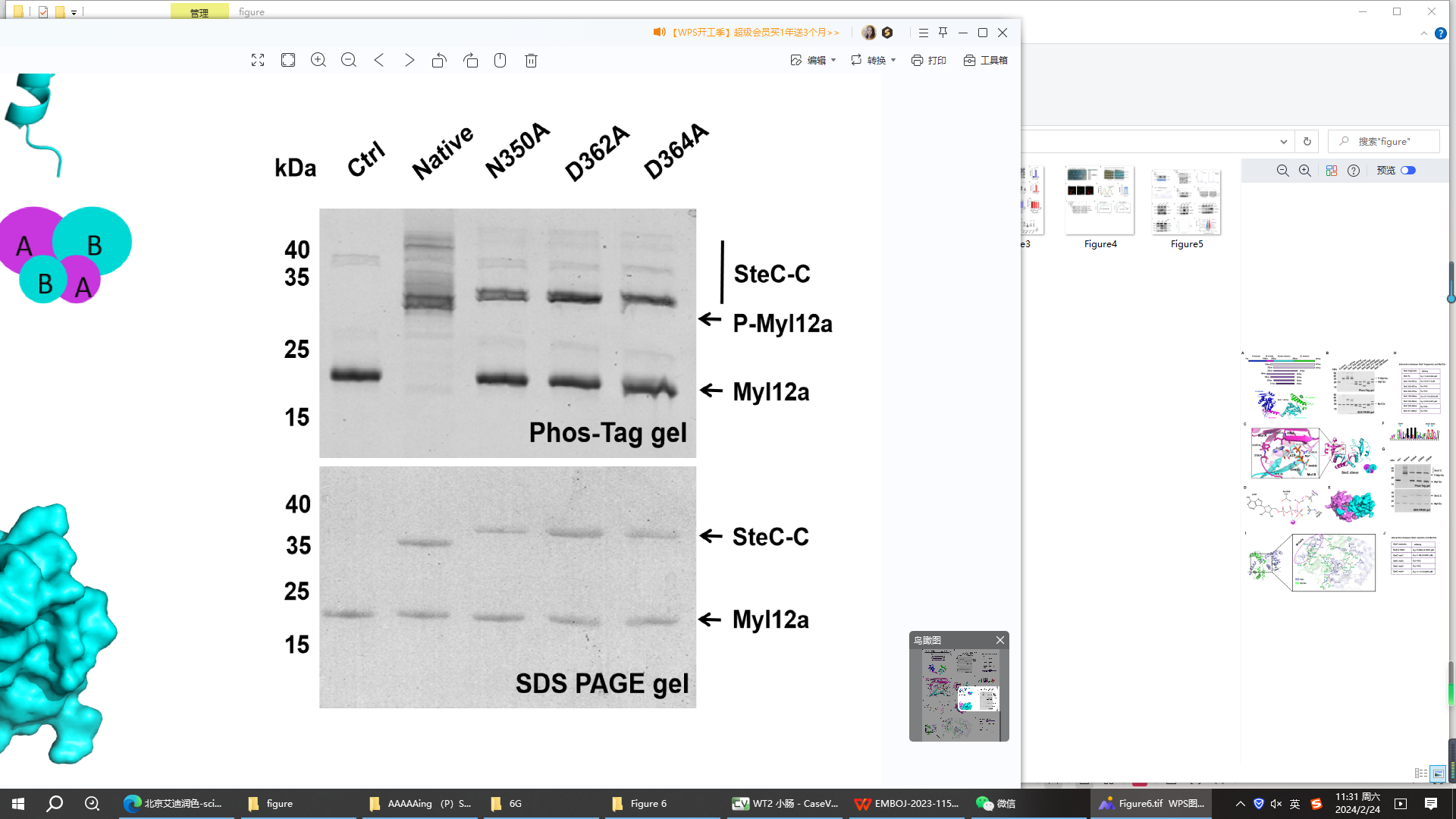


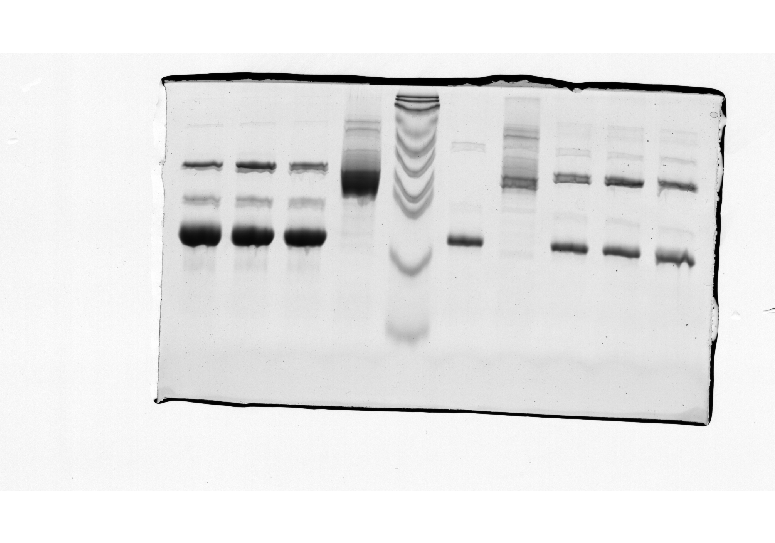


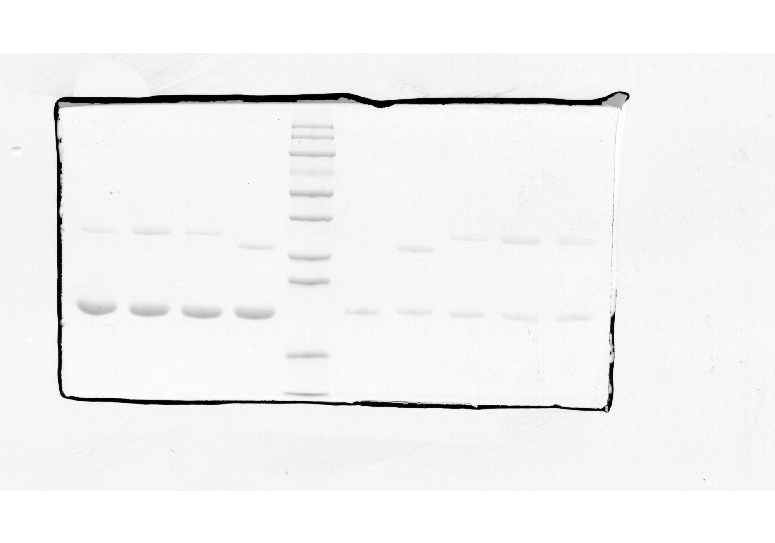

Supplement: Supplementary file 10 — Source data Fig. 6 [file 44318_2024_76_MOESM10_ESM.zip › Figure6/6G/READ ME.docx]

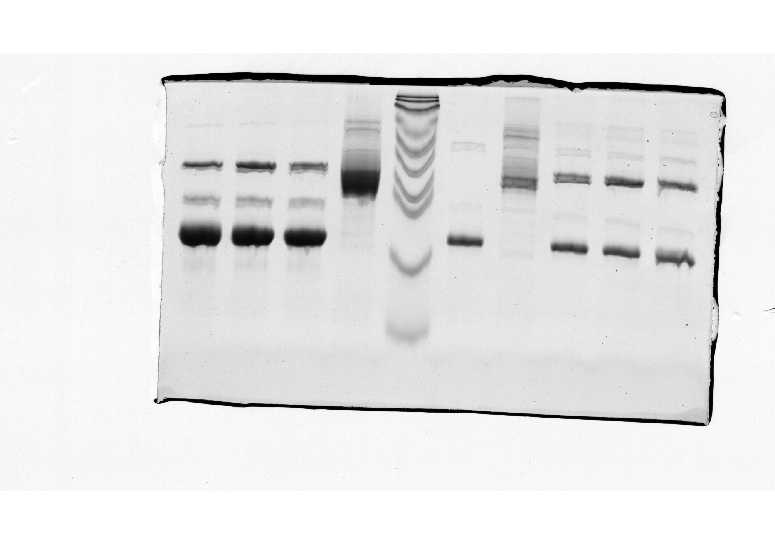

Supplement: Supplementary file 10 — Source data Fig. 6 [file 44318_2024_76_MOESM10_ESM.zip › Figure6/6G/phos tag.tif]

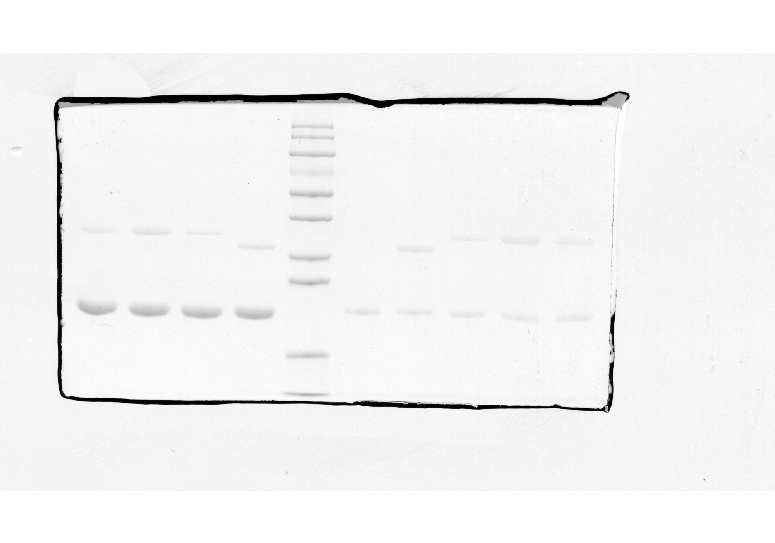

Supplement: Supplementary file 10 — Source data Fig. 6 [file 44318_2024_76_MOESM10_ESM.zip › Figure6/6G/sds page.tif]

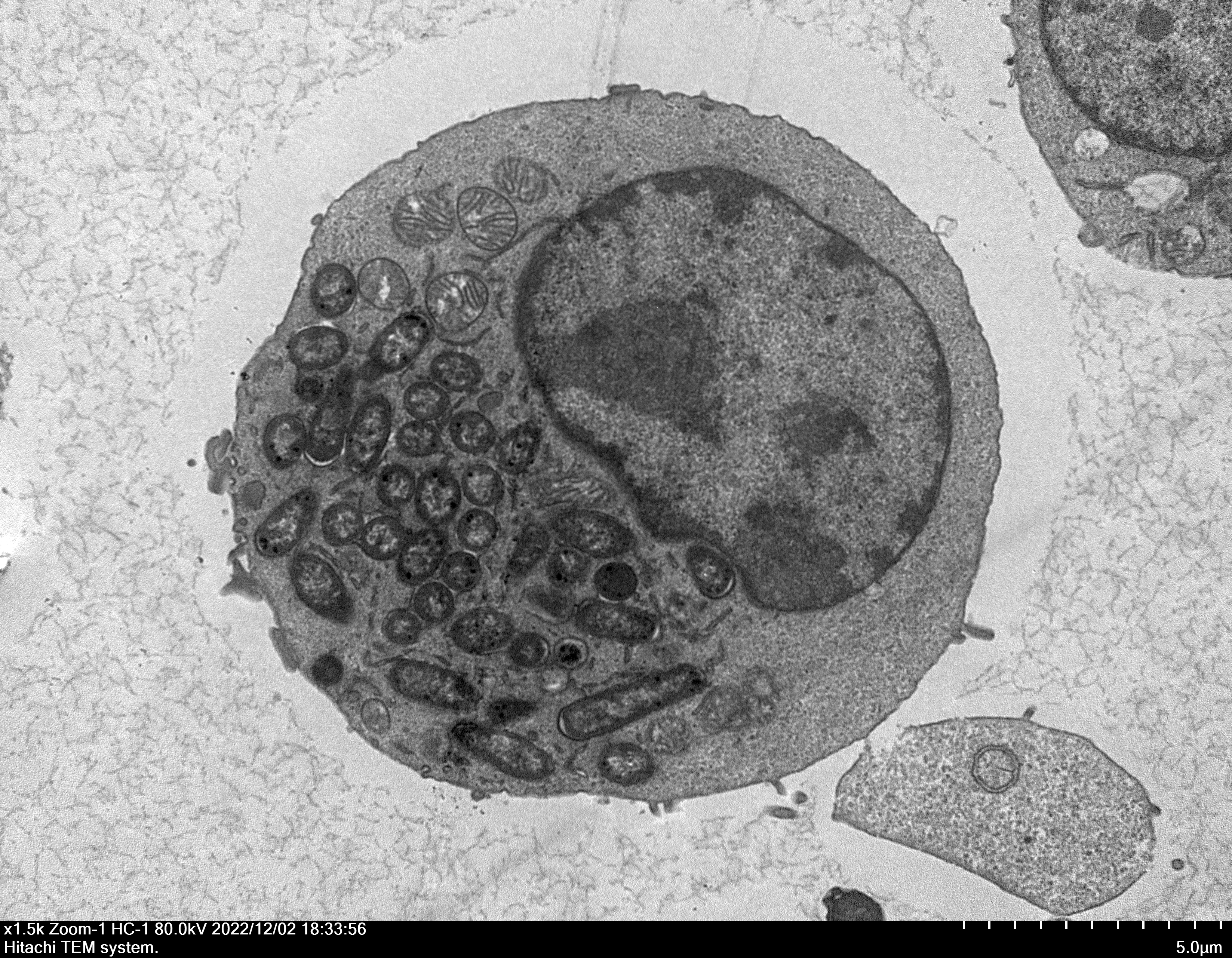

Supplement: Supplementary file 11 — Source data Fig. 7 [file 44318_2024_76_MOESM11_ESM.zip › Figure7/7B/STEC ko.tif]

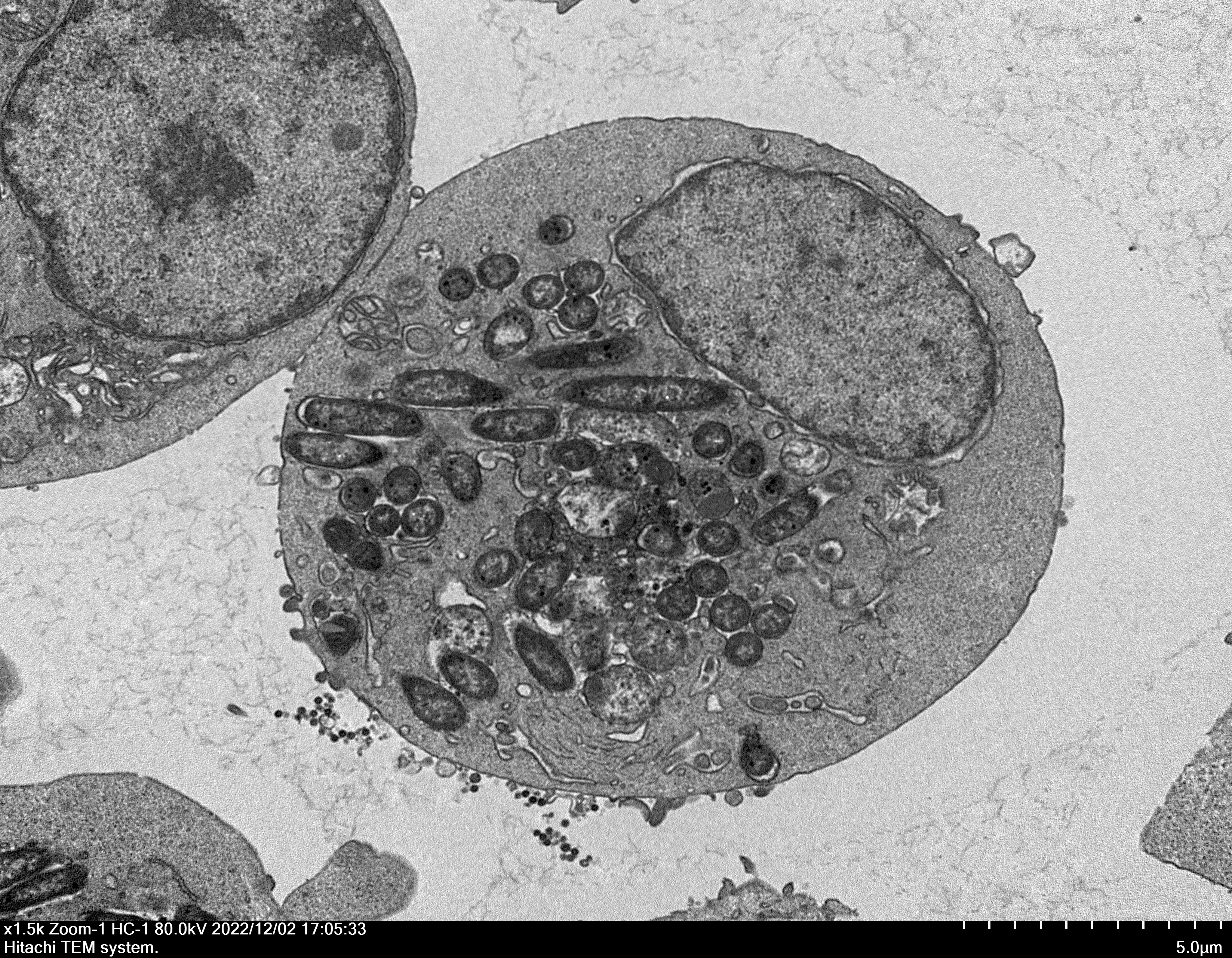

Supplement: Supplementary file 11 — Source data Fig. 7 [file 44318_2024_76_MOESM11_ESM.zip › Figure7/7B/WT.tif]

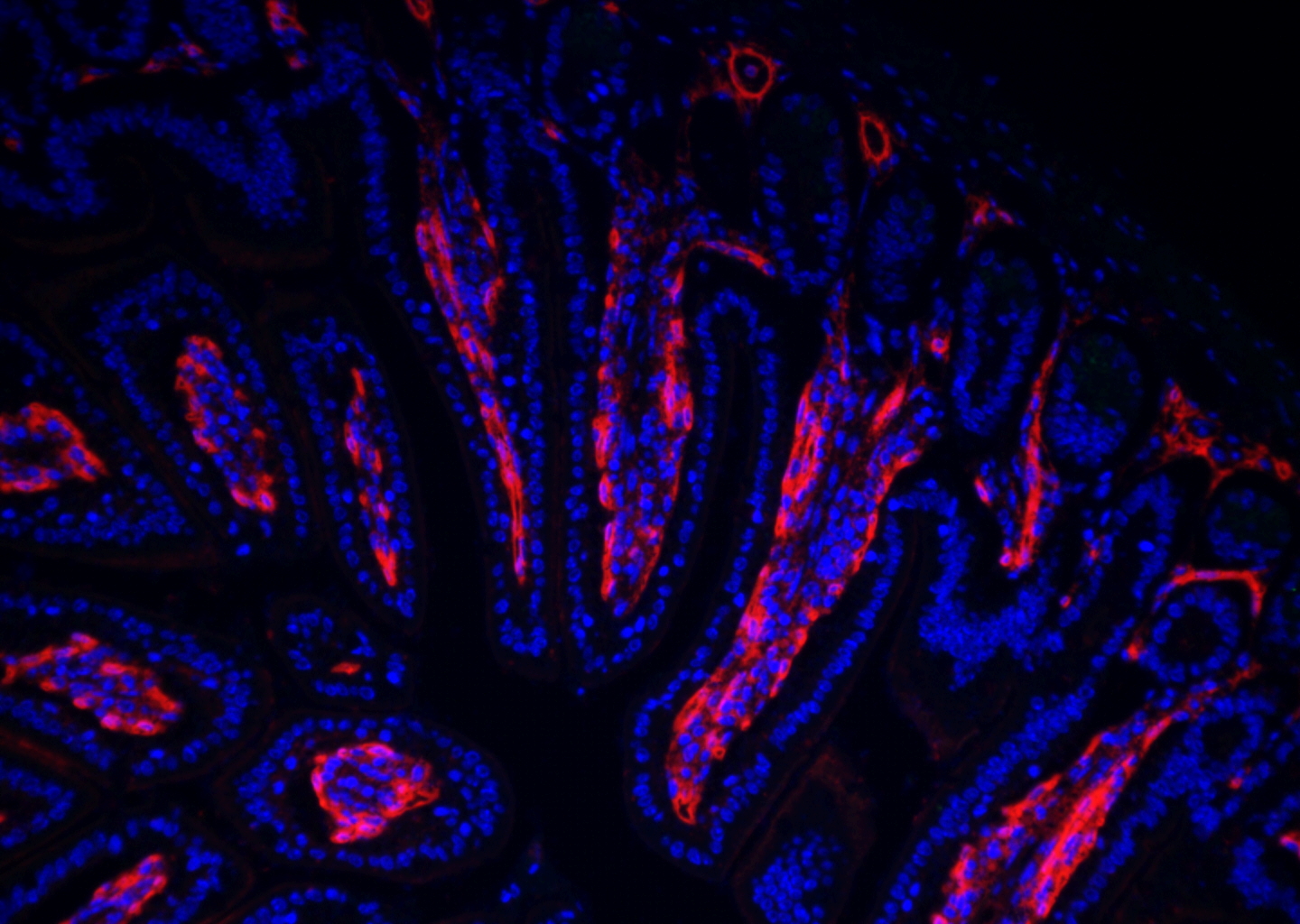

Supplement: Supplementary file 11 — Source data Fig. 7 [file 44318_2024_76_MOESM11_ESM.zip › Figure7/7F/WT20-1小肠2 PV1红+EGFP绿 200-4 5 6.jpg]

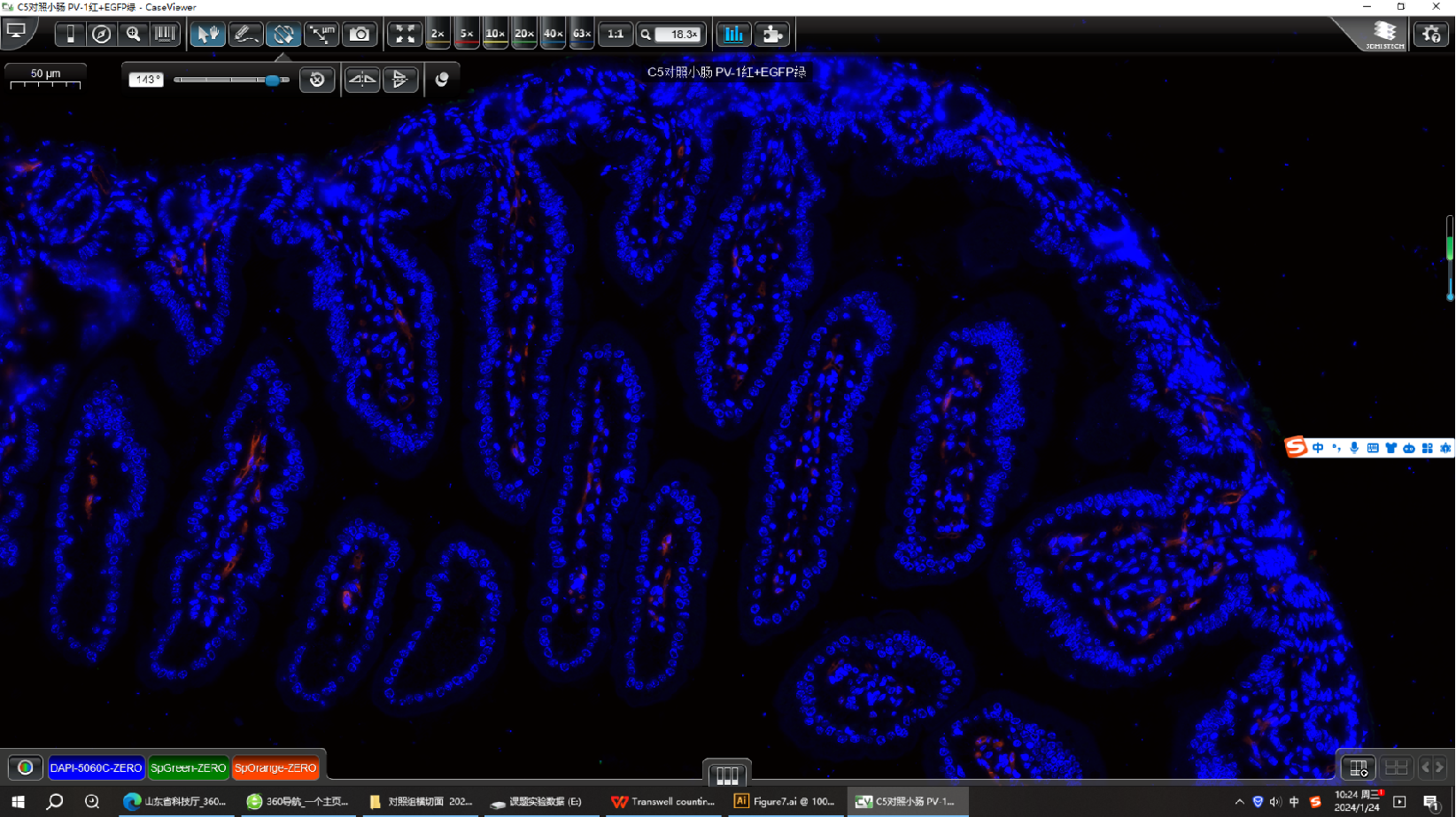

Supplement: Supplementary file 11 — Source data Fig. 7 [file 44318_2024_76_MOESM11_ESM.zip › Figure7/7F/uninfected.png]

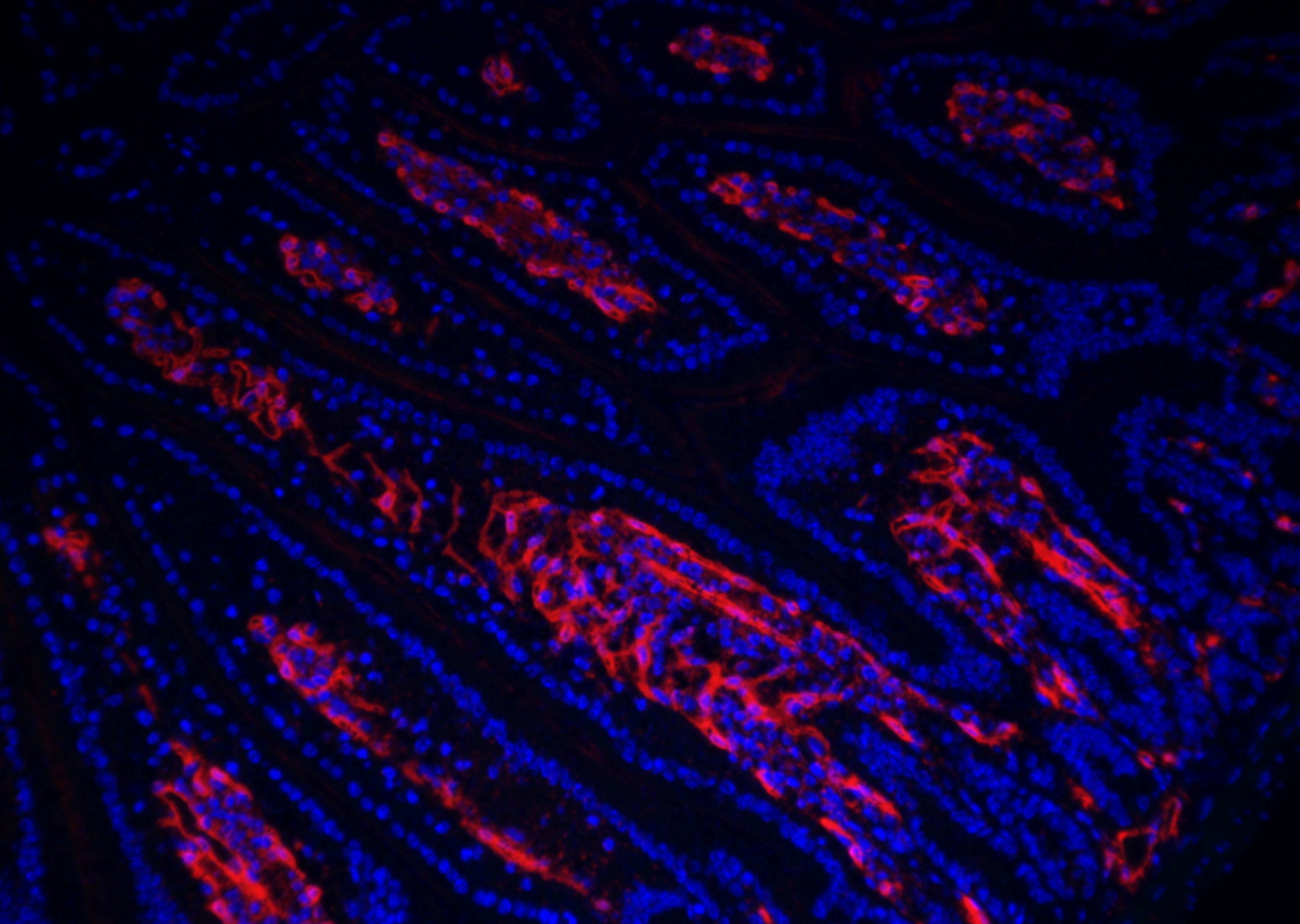

Supplement: Supplementary file 11 — Source data Fig. 7 [file 44318_2024_76_MOESM11_ESM.zip › Figure7/7F/△steC20-1小肠 PV1红+EGFP绿 200-1 2 3.jpg]

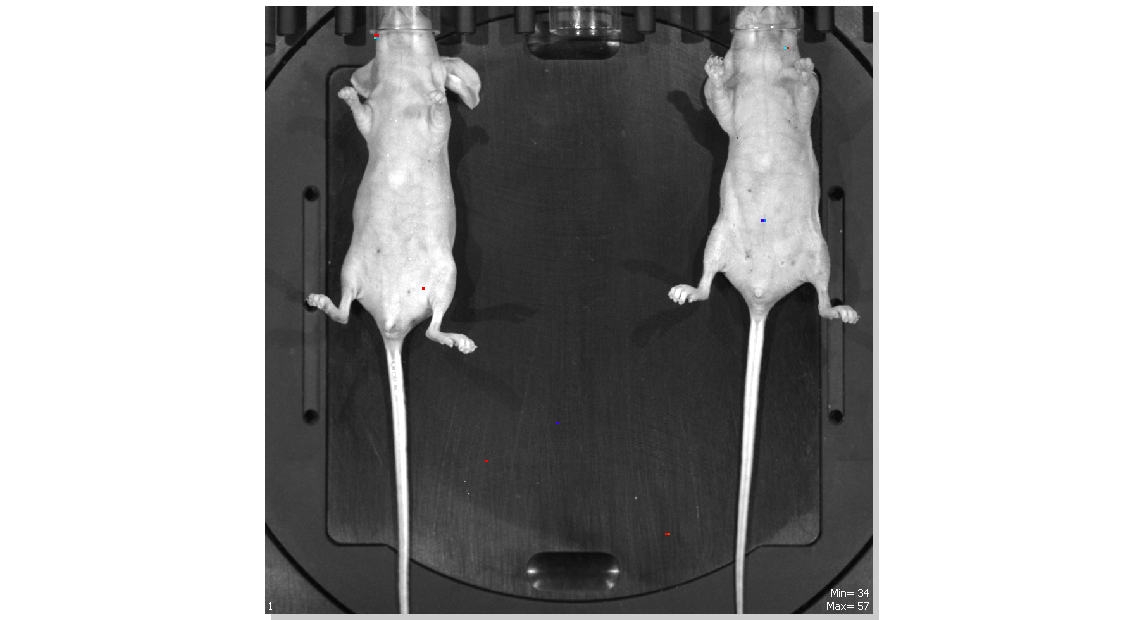

Supplement: Supplementary file 11 — Source data Fig. 7 [file 44318_2024_76_MOESM11_ESM.zip › Figure7/7G/14-1.PNG]

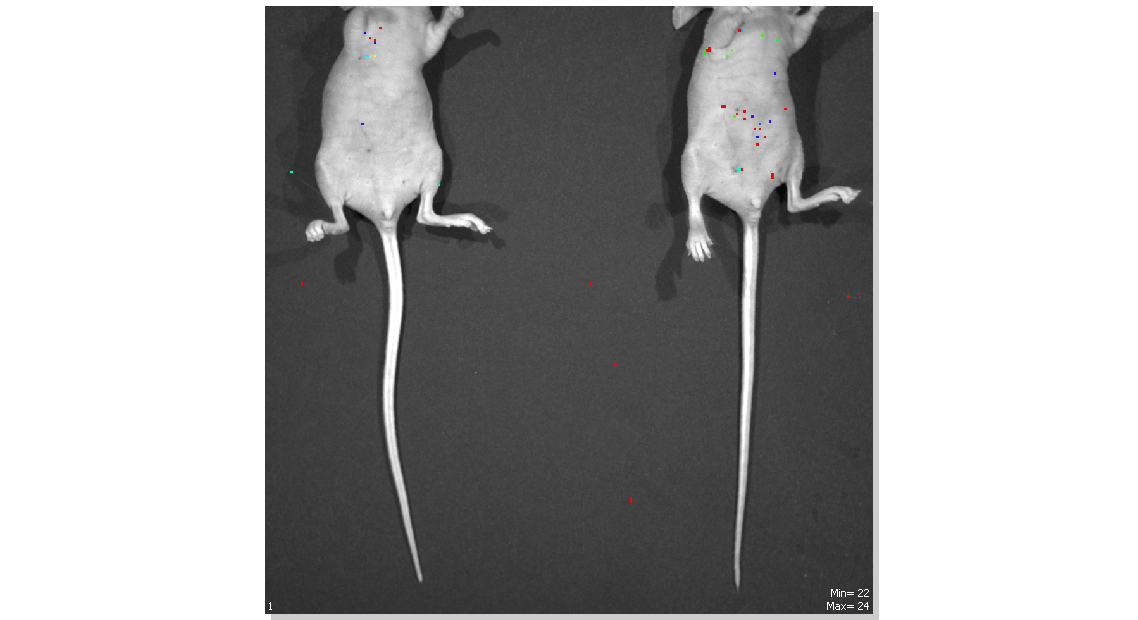

Supplement: Supplementary file 11 — Source data Fig. 7 [file 44318_2024_76_MOESM11_ESM.zip › Figure7/7G/14-2.PNG]

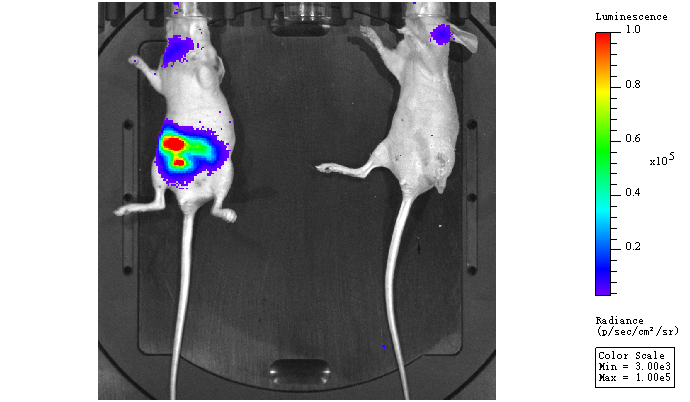

Supplement: Supplementary file 11 — Source data Fig. 7 [file 44318_2024_76_MOESM11_ESM.zip › Figure7/7G/16-1.png]

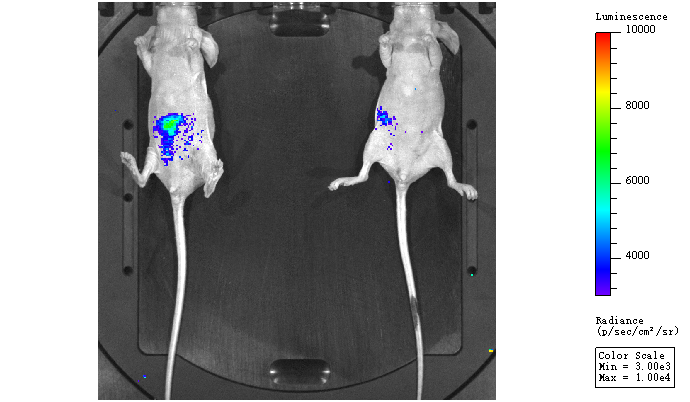

Supplement: Supplementary file 11 — Source data Fig. 7 [file 44318_2024_76_MOESM11_ESM.zip › Figure7/7G/16-2.png]

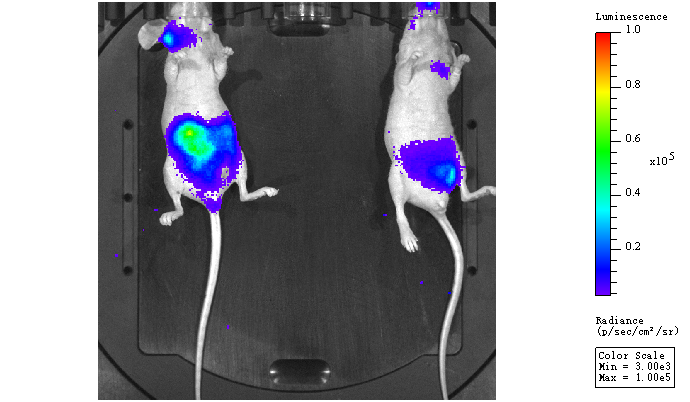

Supplement: Supplementary file 11 — Source data Fig. 7 [file 44318_2024_76_MOESM11_ESM.zip › Figure7/7G/18-1.png]

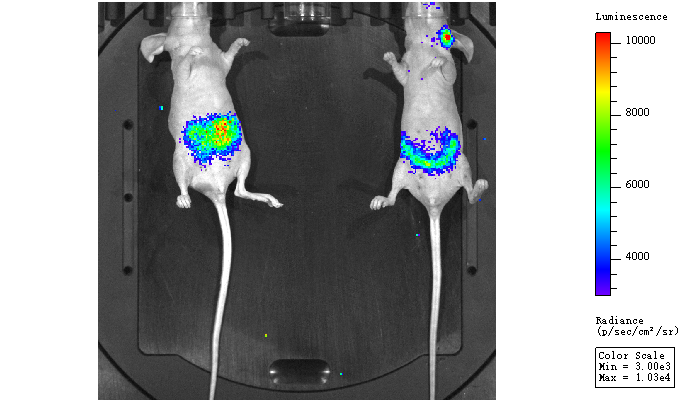

Supplement: Supplementary file 11 — Source data Fig. 7 [file 44318_2024_76_MOESM11_ESM.zip › Figure7/7G/18-2.png]

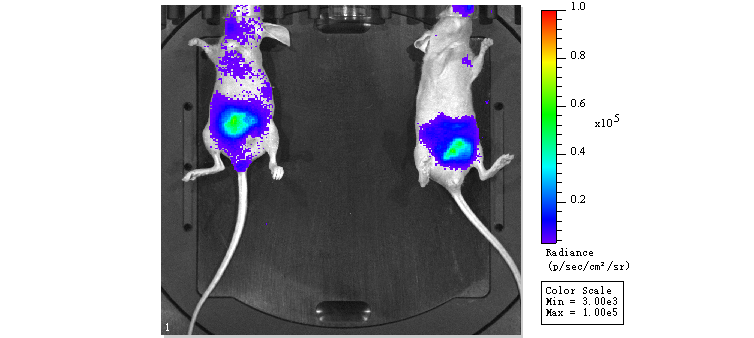

Supplement: Supplementary file 11 — Source data Fig. 7 [file 44318_2024_76_MOESM11_ESM.zip › Figure7/7G/20-1.png]

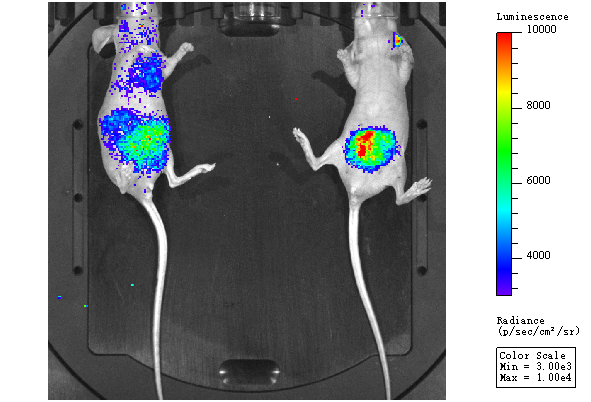

Supplement: Supplementary file 11 — Source data Fig. 7 [file 44318_2024_76_MOESM11_ESM.zip › Figure7/7G/20-2.png]

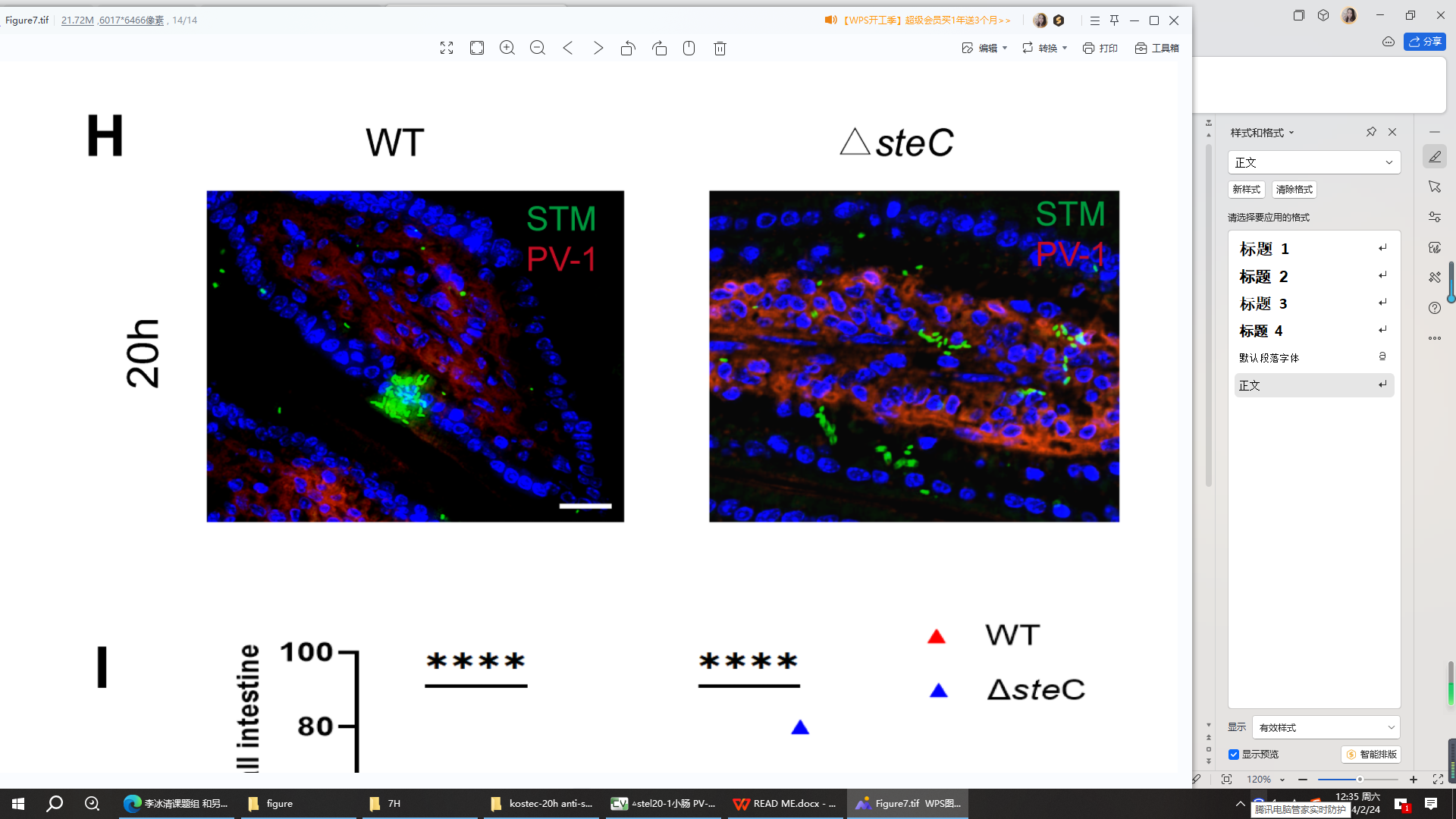


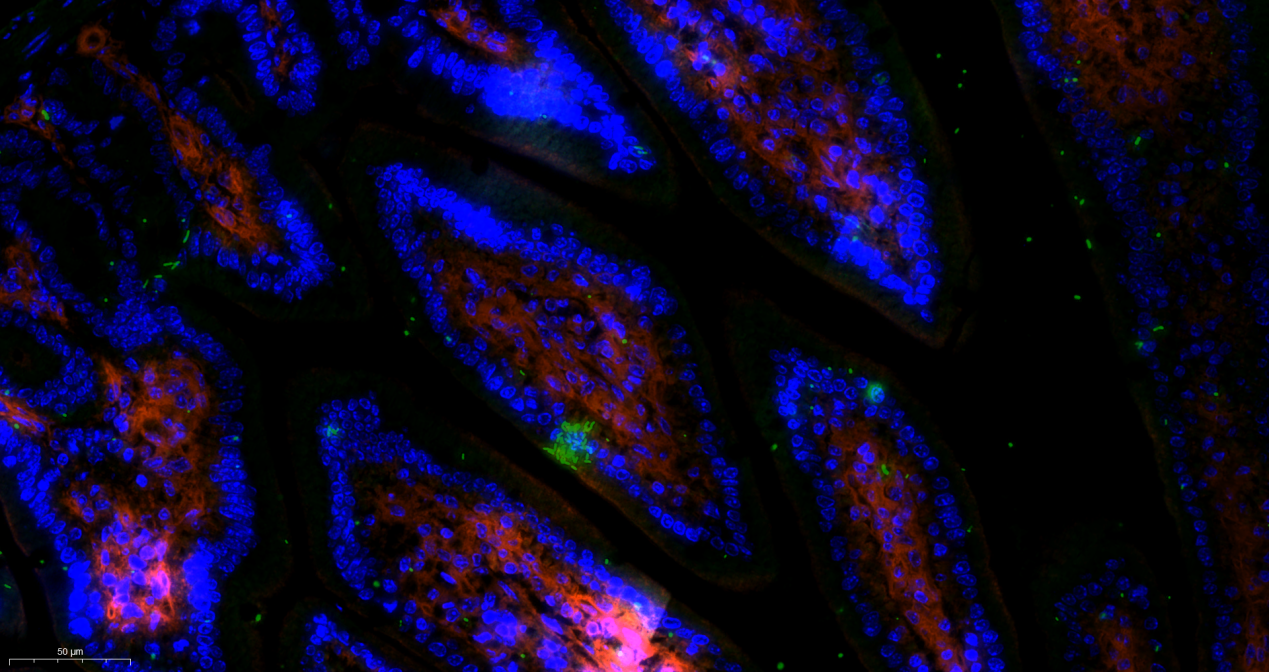


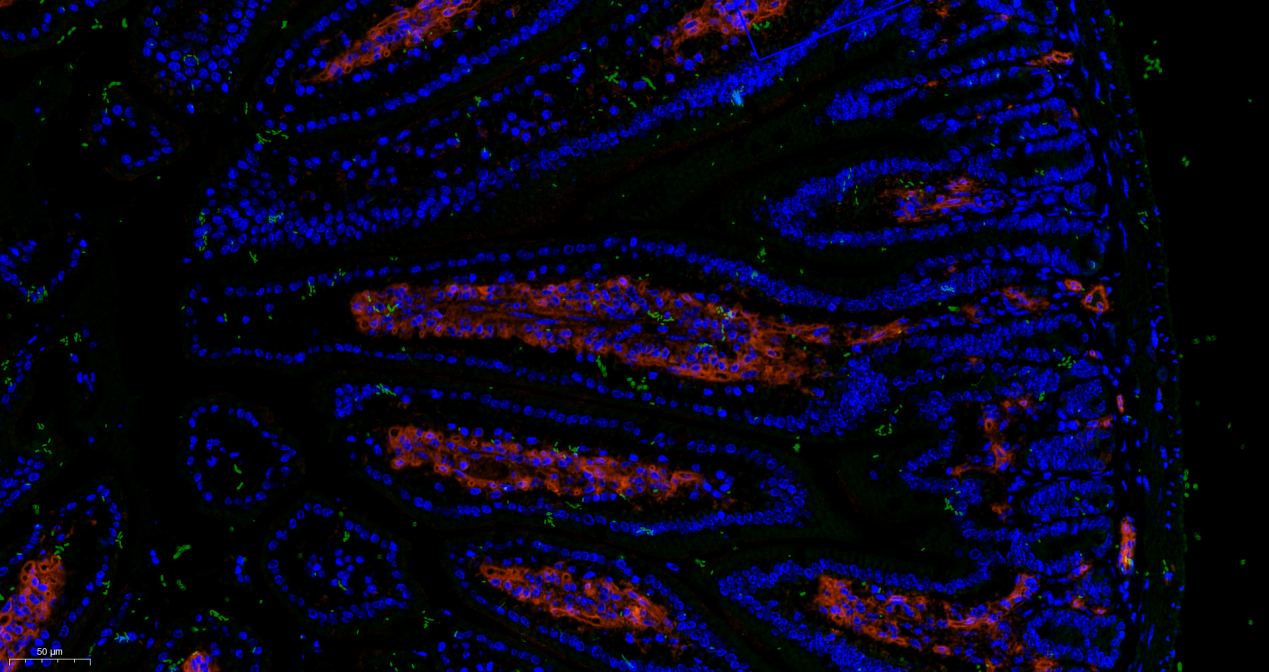

Supplement: Supplementary file 11 — Source data Fig. 7 [file 44318_2024_76_MOESM11_ESM.zip › Figure7/7H/READ ME.docx]

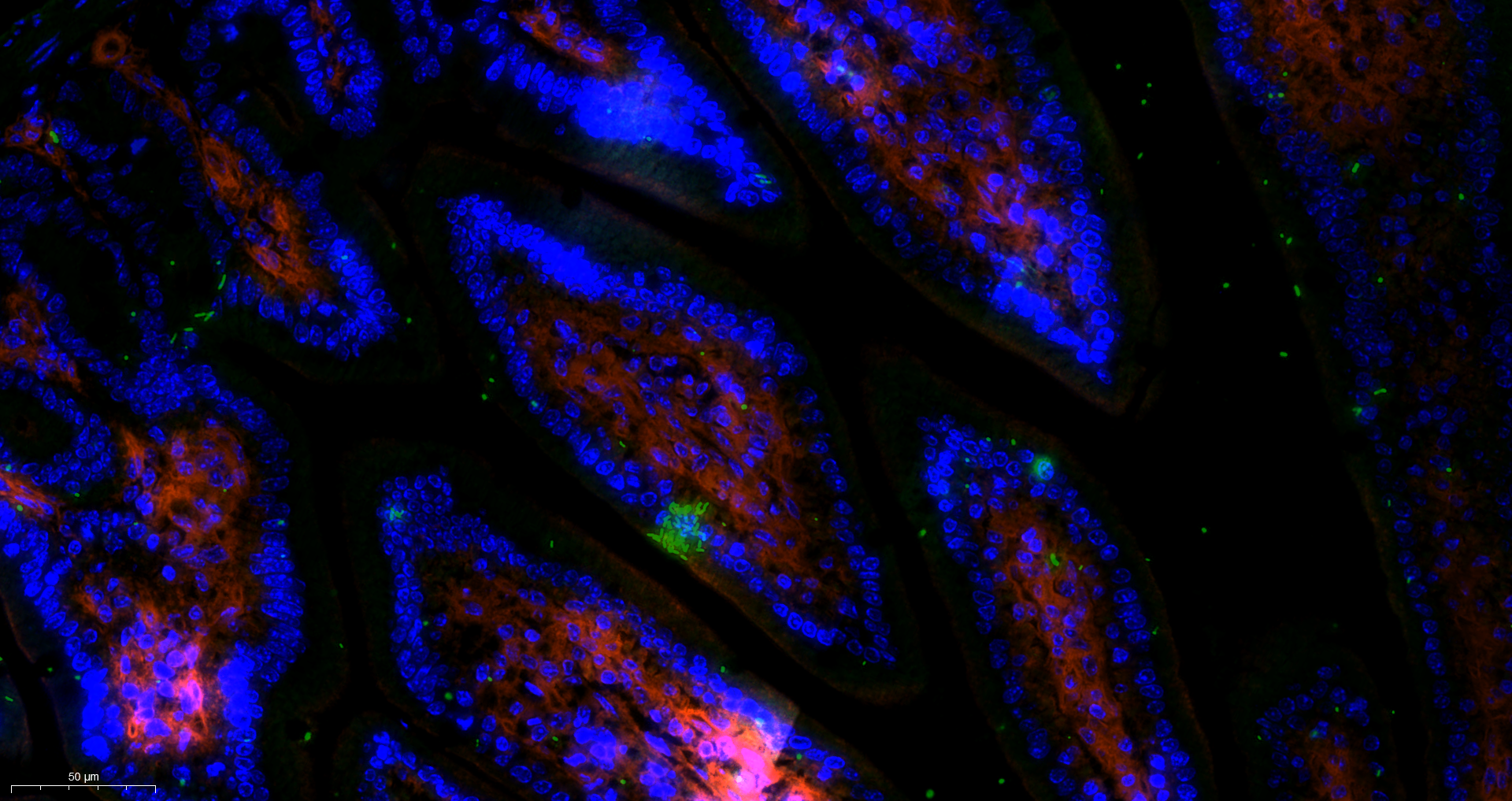

Supplement: Supplementary file 11 — Source data Fig. 7 [file 44318_2024_76_MOESM11_ESM.zip › Figure7/7H/WT20H-1 PV-1RED+Saleonella GREEN_36.5x.tif]

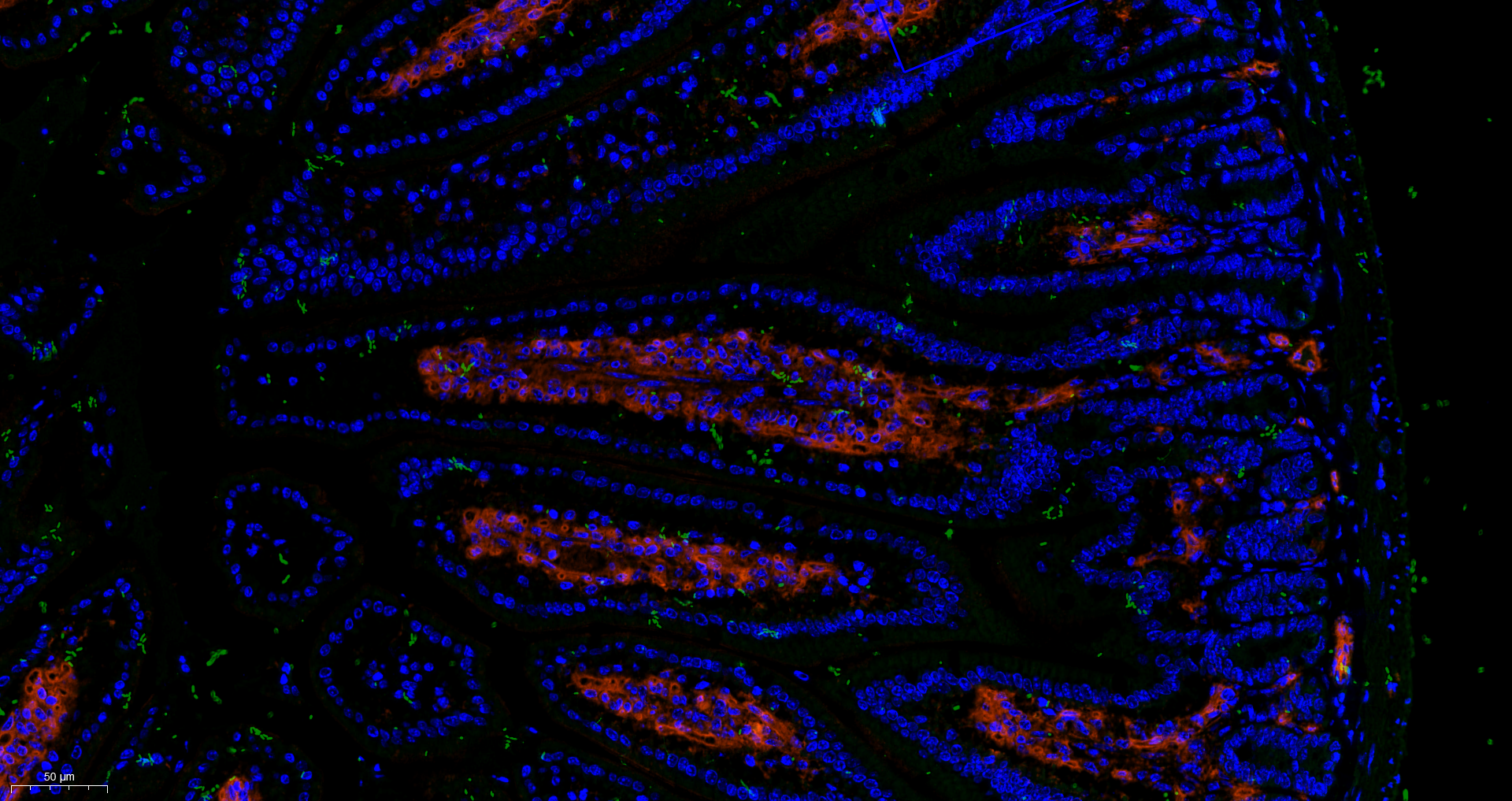

Supplement: Supplementary file 11 — Source data Fig. 7 [file 44318_2024_76_MOESM11_ESM.zip › Figure7/7H/△steC20H-1 PV-1RED+sdmonella GREEN_24.4x.tif]

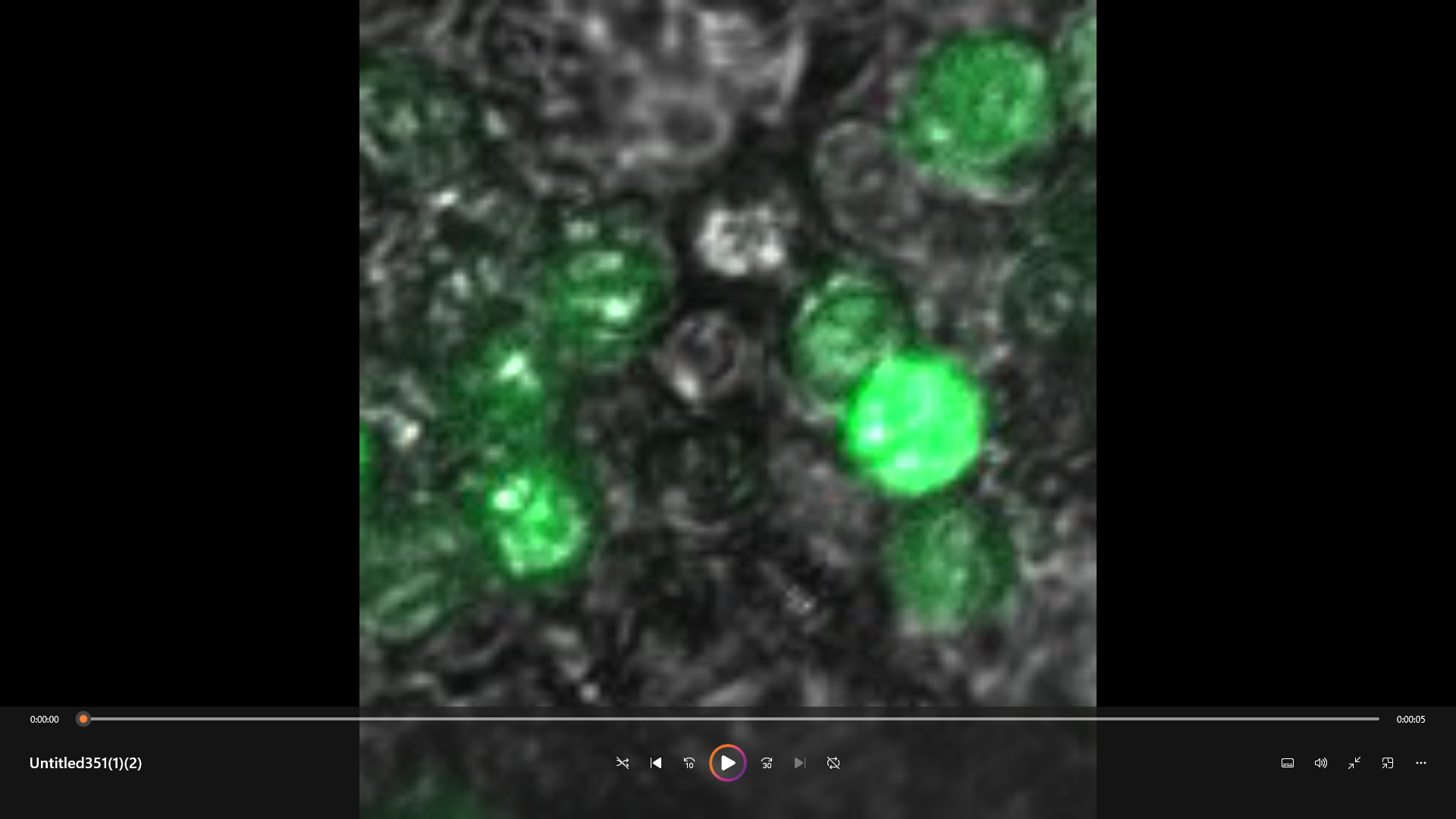

Supplement: Supplementary file 11 — Source data Fig. 7 [file 44318_2024_76_MOESM11_ESM.zip › Figure7/7L/1.tif]

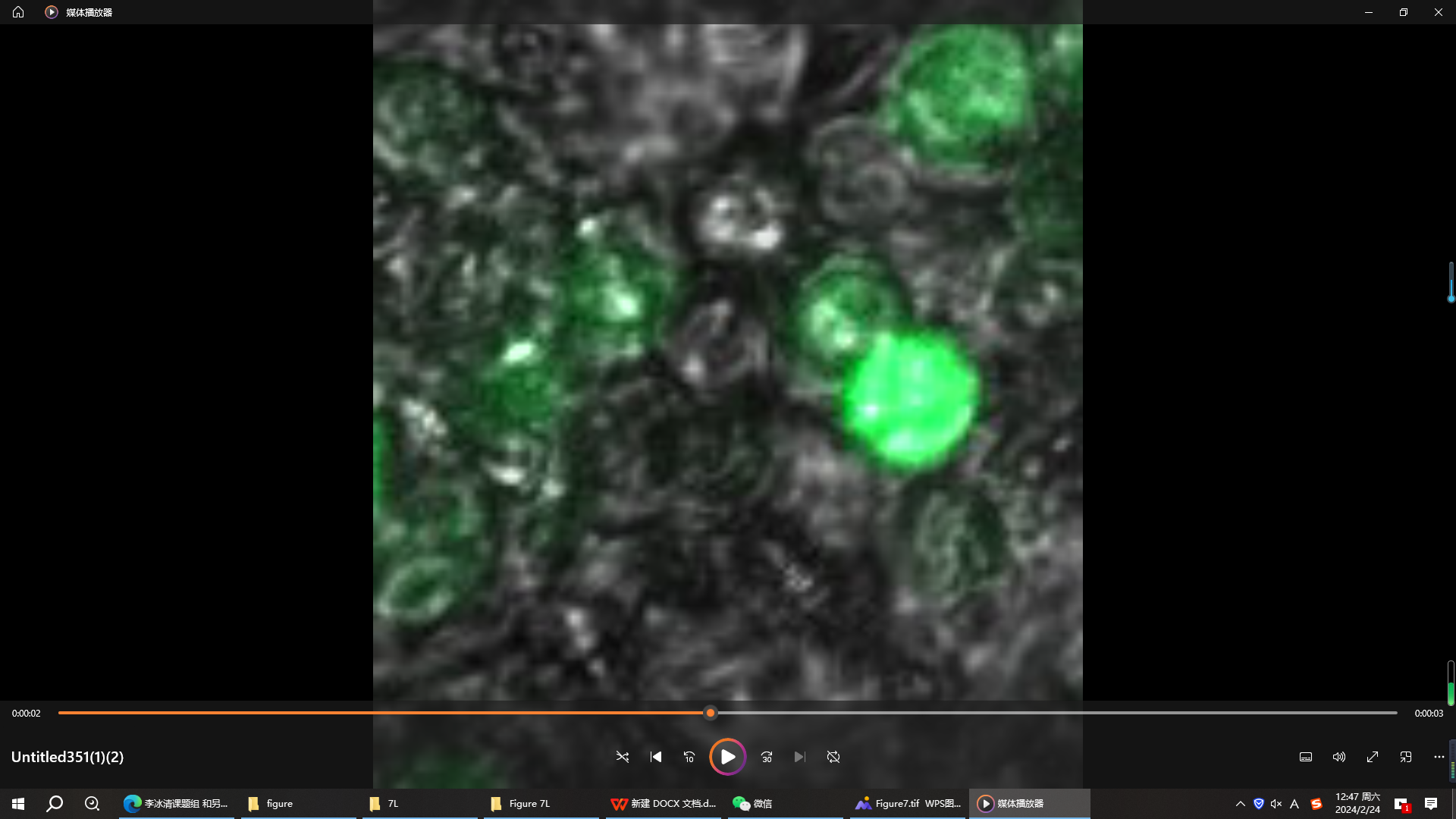

Supplement: Supplementary file 11 — Source data Fig. 7 [file 44318_2024_76_MOESM11_ESM.zip › Figure7/7L/2.tif]

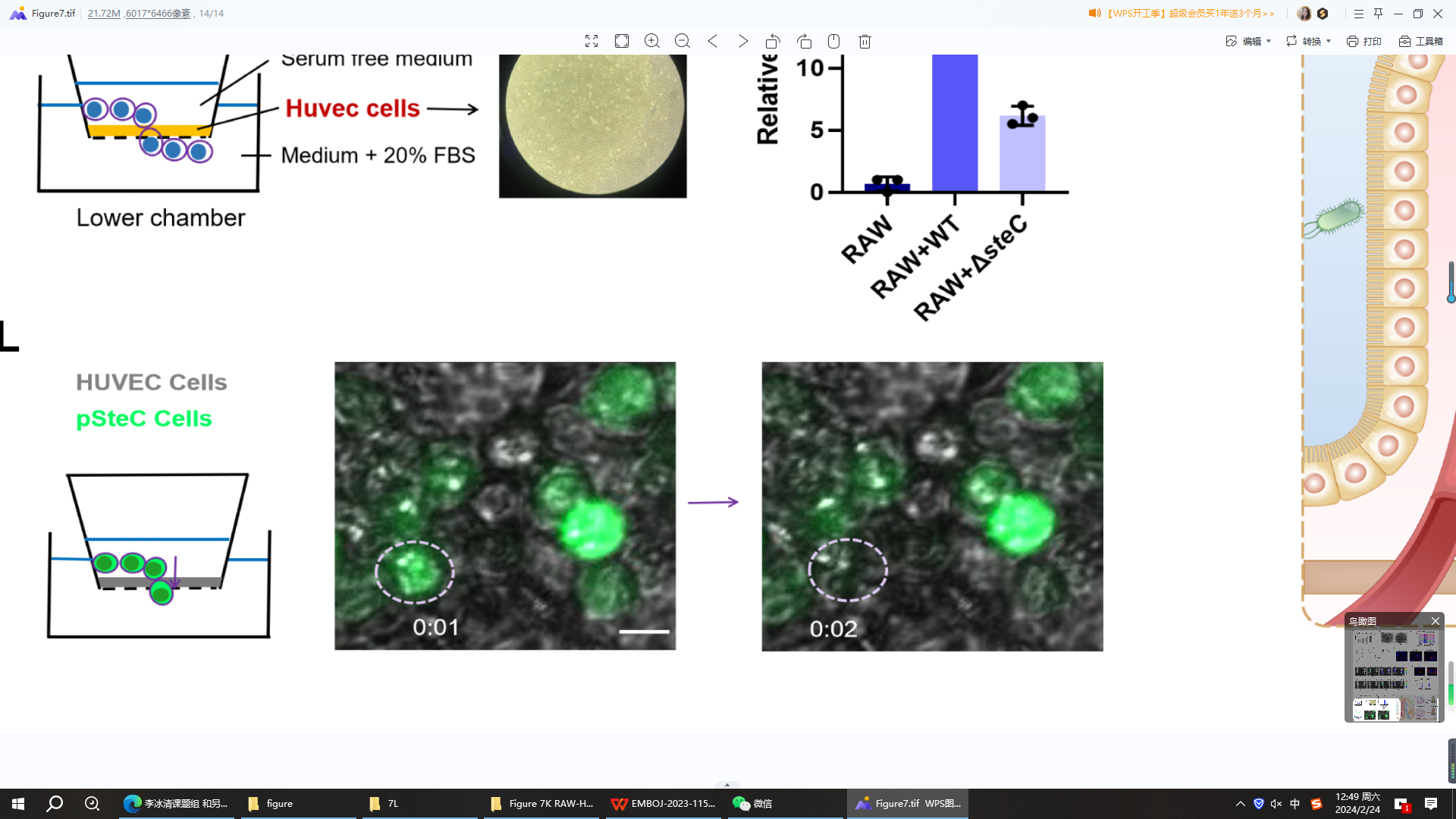


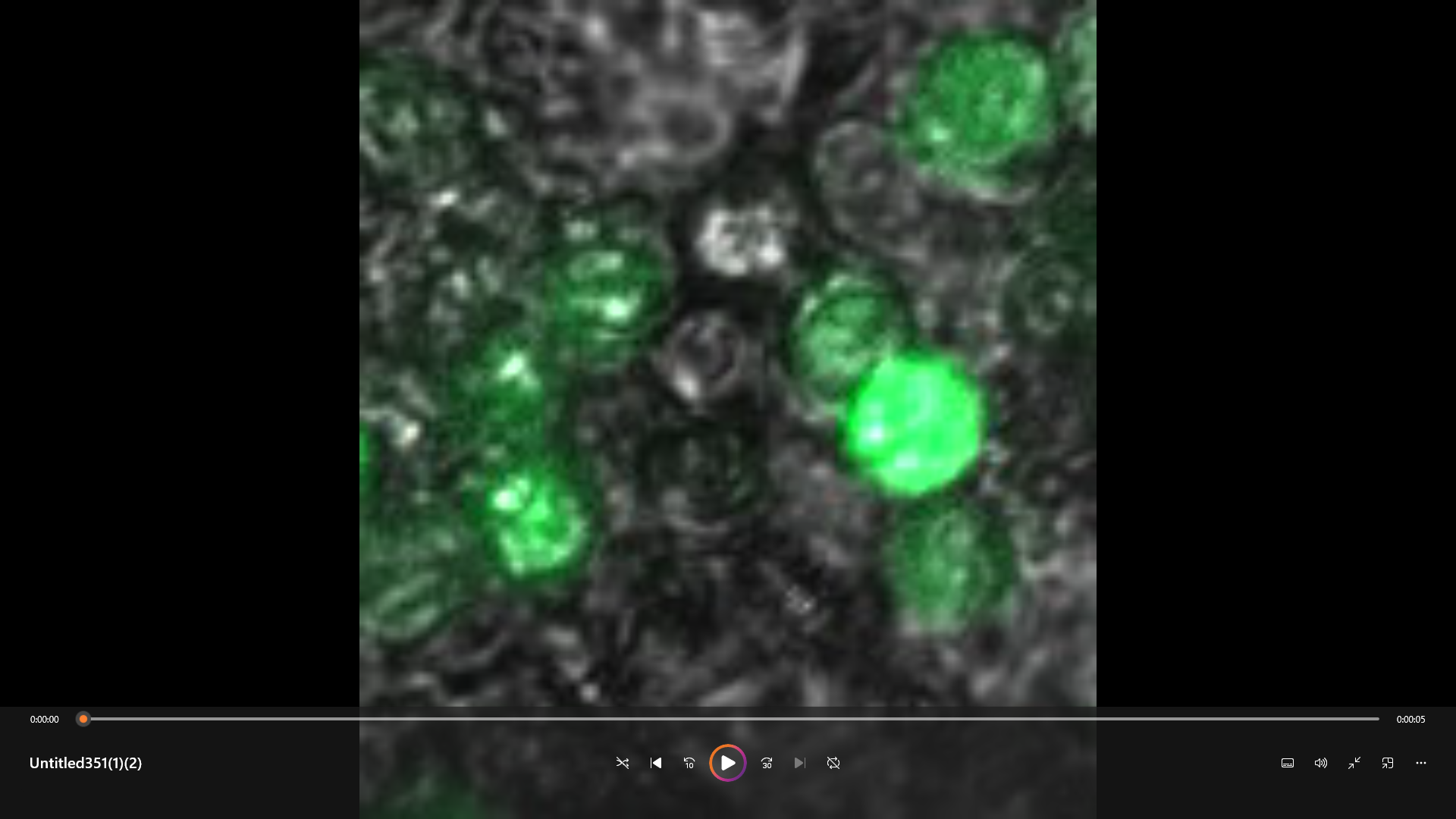


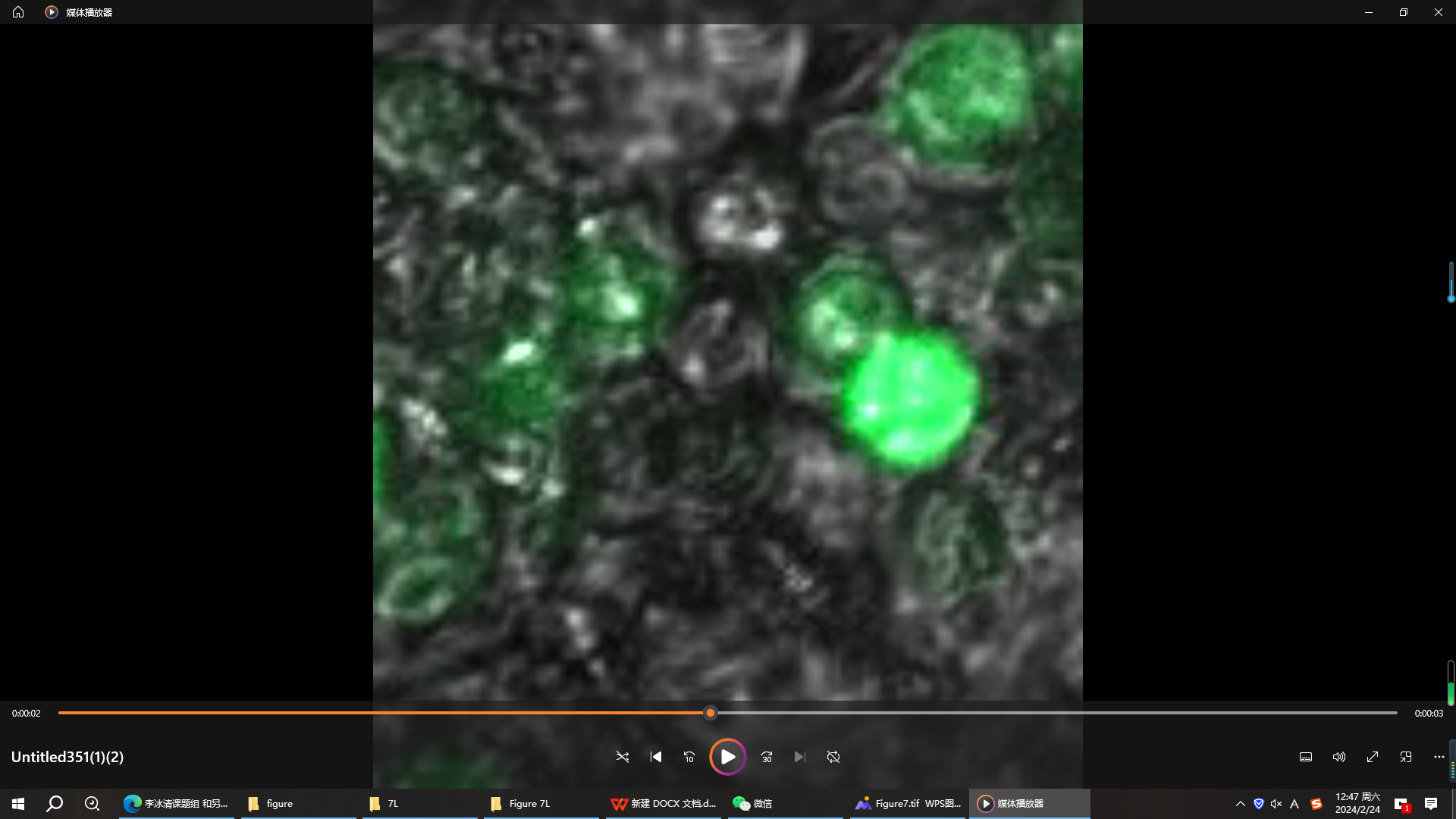

Supplement: Supplementary file 11 — Source data Fig. 7 [file 44318_2024_76_MOESM11_ESM.zip › Figure7/7L/readme.docx]

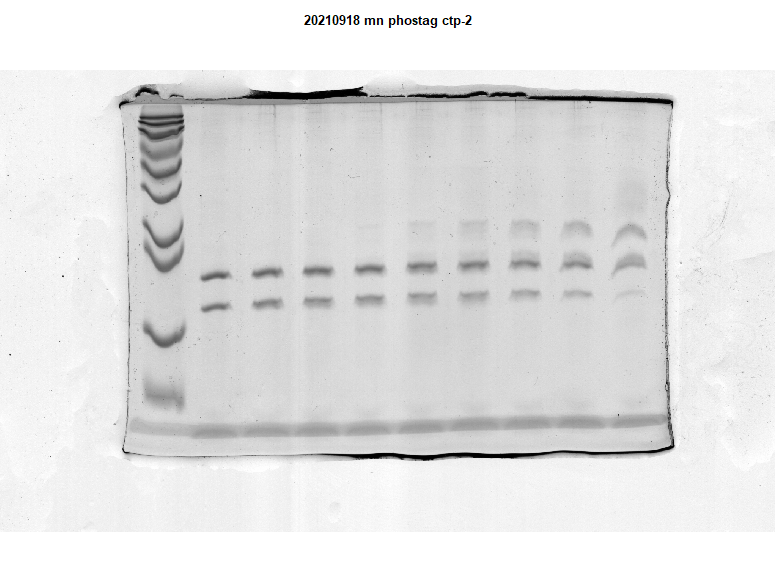

Supplement: Supplementary file 12 — Appendix figure Source Data [file 44318_2024_76_MOESM12_ESM.zip › Fig.S4C/20210918 mn CTP.tif]

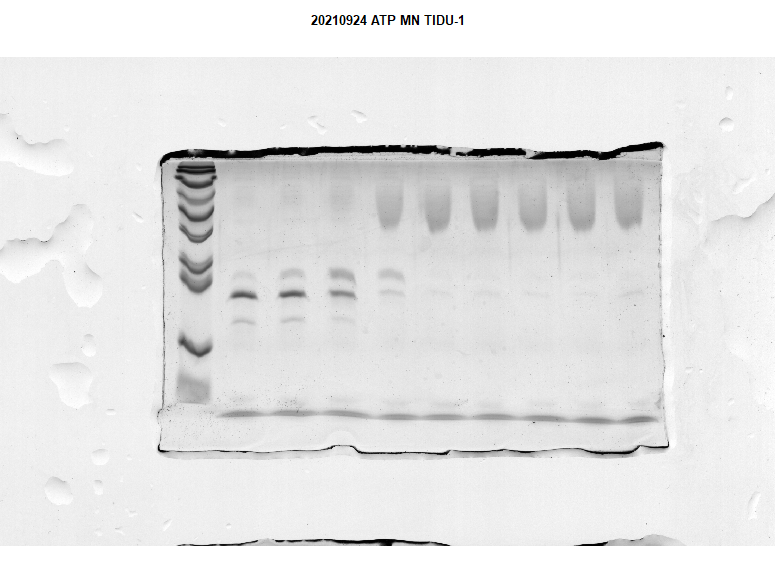

Supplement: Supplementary file 12 — Appendix figure Source Data [file 44318_2024_76_MOESM12_ESM.zip › Fig.S4C/20210924 mn ATP.tif]

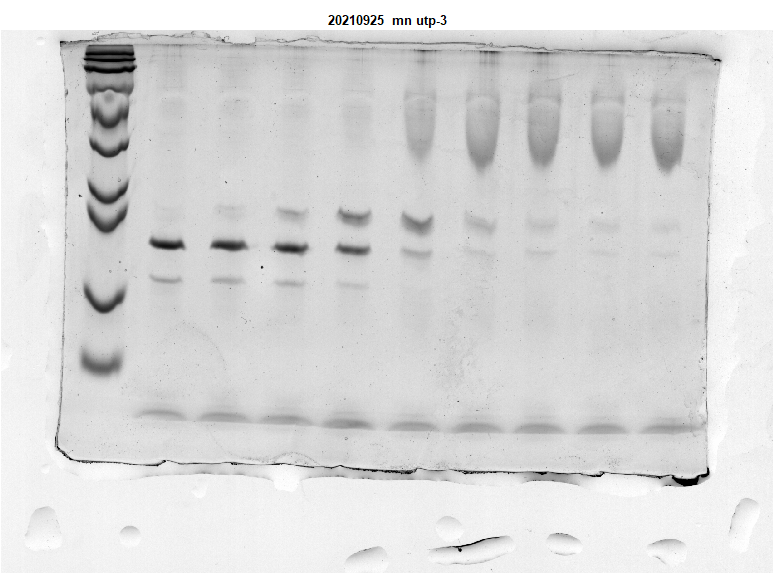

Supplement: Supplementary file 12 — Appendix figure Source Data [file 44318_2024_76_MOESM12_ESM.zip › Fig.S4C/20210925 mn UTP.tif]

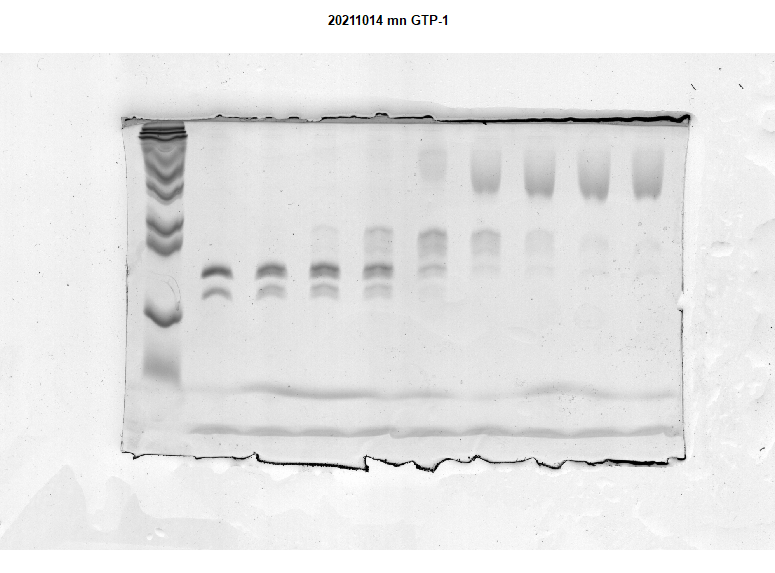

Supplement: Supplementary file 12 — Appendix figure Source Data [file 44318_2024_76_MOESM12_ESM.zip › Fig.S4C/20211014 mn GTP.tif]
