# Supplementary material for: Salmonella manipulates macrophage migration via SteC-mediated myosin light chain activation to penetrate the gut-vascular barrier
Source: EMBO J. 2024 Mar 25;43(8):1499–518. doi: 10.1038/s44318-024-00076-7 (PMC11021425; doi:10.1038/s44318-024-00076-7)
Supplement: Supplementary file 8 — Source data Fig. 4 [file 44318_2024_76_MOESM8_ESM.zip › Figure4/4G/READ ME.docx]

In this experiment, the BLI method was employed to detect SteC and Myl12a interaction using the OctetRED96e instrument. The software automatically calculates the Kd value and its corresponding error. Therefore, we provide the system-exported raw files for your review.
